# Prevalence of maternal substance use problems during pregnancy and the first 2 years of life: a whole-population birth cohort of 970 470 Australian children born 2008–2017

**DOI:** 10.1136/jech-2024-223439

**Published:** 2025-03-24

**Authors:** Madeleine Powell, Rhiannon Pilkington, Tasnia Ahmed, Mark Hanly, BJ Newton, John W Lynch, Timothy Dobbins, Jessica Stewart, Michelle Cretikos, Alys Havard, Kathleen Falster

**Affiliations:** 1 School of Population Health, National Drug and Alcohol Research Centre, University of New South Wales, Sydney, New South Wales, Australia; 2 School of Public Health, The University of Adelaide, Adelaide, South Australia, Australia; 3 School of Population Health, University of New South Wales, Sydney, New South Wales, Australia; 4 Centre for Big Data Research in Health, University of New South Wales, Sydney, New South Wales, Australia; 5 Social Policy Research Centre, University of New South Wales, Sydney, New South Wales, Australia; 6 Family and Community Services Insights, Analysis and Research, NSW Department of Communities and Justice, Sydney, New South Wales, Australia; 7 Centre for Epidemiology and Evidence, Ministry of Health, Sydney, New South Wales, Australia

**Keywords:** EPIDEMIOLOGY, CHILD HEALTH, PREVENTION, SUBSTANCE ABUSE, MATERNAL HEALTH

## Abstract

**Objectives:**

To estimate the prevalence of maternal substance use problems during the first 1000 days of children’s lives, to inform planning and resourcing of antenatal screening and substance use in pregnancy services, alongside antenatal and postnatal health, parenting and social support services for pregnant women/new mothers and their babies.

**Method:**

This whole-population cohort was assembled from birth registration, perinatal and hospital data for children born 2008–2017, and their mothers, using data linked for the New South Wales (NSW) Child E-Cohort Project. The primary outcome was maternal substance use conditions and treatment recorded in six health, death and child protection data sources from the child’s conception to age 2 years (the first 1000 days), including illicit substances, alcohol, opioid-agonist treatment and misuse of psychoactive medicines or substances.

**Results:**

Of 970 470 children born to 625 856 mothers, 3.4% (N=32 647) had ≥1 maternal substance use problem records in the first 1000 days, including alcohol use (N=13 637; 1.4%) and other drug use (N=23 485; 2.4%). Maternal substance use problems were recorded during the pregnancy period for 1.4% of children, and from 28 to 1000 days postbirth for 2.7% of children. Outcome ascertainment was highest from child protection records (N=26 045), followed by mother’s (N=10 793) then children’s hospital records (N=3827). Child protection records more than doubled the prevalence of health and death records alone (1.4%). Social and health disadvantage was more common among children with maternal substance use problems.

**Conclusions:**

During the first 1000 days of life, 3.4% of NSW children had ≥1 maternal substance use problem recorded in health, child protection and death data sources. Child protection data enhance public health intelligence on the burden of maternal substance use problems among whole child populations. Near universal health system contact during pregnancy and birth is an opportunity to initiate early support for maternal substance use and co-occurring health and social disadvantage, to promote child health and development.

**WHAT IS ALREADY KNOWN ON THIS TOPIC**

- The prevalence and profile of maternal substance use problems in pregnancy and early life are needed to inform substance use in pregnancy and related support service delivery that meets the needs of affected mothers and children.
- While the prevalence of any substance use reported by pregnant women in survey samples and prenatal screening ranges from 10% to 18%, estimates of more harmful maternal substance use during pregnancy lie between 0.2% and 3% of mothers based on records in (mostly) hospital and other health administrative datasets.

**WHAT THIS STUDY ADDS**

- This study shows that 3.4% of the 970 470 Australian children born 2008–2017 in New South Wales had maternal substance use problems recorded during their first 1000 days of life in ≥1 of the six linked administrative datasets for mothers and children; the addition of child protection data to health and death data sources more than doubled the prevalence estimates, from 1.4% to 3.4%.

**HOW THIS STUDY MIGHT AFFECT RESEARCH, PRACTICE OR POLICY**

- This study suggests at least 1 in 30 mothers and infants may benefit from early screening and support services for maternal substance use problems and co-occurring health and social disadvantage to promote child health and development.
- Near universal health system contact during pregnancy and birth is an opportunity to initiate early support for maternal substance use and co-occurring health and social disadvantage, to promote child health and development.

## Introduction

 Maternal substance use may harm child health and development, both through exposure during gestation^1^ and impacts on parenting, family functioning and the home environment during early life.^2^,^4^ Screening and support for pregnant women and new mothers using substances is important for the health of mothers and to promote children’s health and development from the earliest opportunity. To plan substance use in pregnancy services and associated prevention responses for mothers and children, contemporary data on the scale and type of maternal substance use problems at the population level are needed.

Until recently, general population cross-sectional surveys have been the main source of data on maternal substance use prevalence during pregnancy. For example, self-reported alcohol use among the subset of pregnant women in survey samples was 8.4% in the USA in 2023,^5^ 15% in Australia in 2023^6^ and 18% in Canada in 2019.^7^ These prevalence estimates capture maternal substance use across the risk spectrum, from infrequent, less harmful use to acute or chronic harmful use. As such, substance use in pregnancy and related support services are not relevant for all of these women and their infants.

Substance use conditions and treatment recorded in administrative healthcare data sources also offer public health intelligence to inform prevention responses to maternal substance problems. To date, most administrative data studies have reported the prevalence of single substance use during pregnancy/birth or neonatal abstinence syndrome (NAS). Some studies have used one data source, often the mother’s or child’s hospital records, to ascertain maternal substance use indicators with estimates such as 1.1% for prenatal illicit substance use^8^; and 0.1%–1% for NAS/in utero substance exposure.^9^,^15^ Higher prevalence estimates have been reported in studies using two to four data sources to ascertain maternal substance use from health service contacts, including hospital and mental health outpatient data (any substance use, 3.2%),^16^ or prenatal screening and primary care data (alcohol use, 3.0%–11.7%; illicit drug use, 3.6%–6.0%).^17^ Although pregnant women may be hesitant to self-report substance use in prenatal screening due to stigma and fear of child protection reporting, the prevalence estimates of any substance use from prenatal screening data are similar to those from general population surveys and higher than the more visible and potentially harmful substance use problems recorded in tertiary healthcare settings such as hospitals.^18^

In this study, we aimed to quantify the prevalence and type of maternal substance use problems recorded in health, death and child protection system data from conception to the child’s second birthday (first 1000 days of life) to inform planning and resourcing of screening and support services that may reduce maternal substance use associated harm or risk for children. We ascertained indicators of maternal substance use conditions and treatment from mother and child records in six health, death and child protection administrative data sources for all children born in New South Wales (NSW), the most populous state in Australia, from 2008 to 2017.

## Methods

This birth cohort study used population-level administrative data linked for the NSW Child E-Cohort Project. Reporting follows the REporting of Studies Conducted using Observational Routinely-collected health Data (RECORD) guidelines (online supplemental file 1).^19^

### Data sources and data linkage

Data sources included perinatal data (includes live births ≥20 weeks gestation or >400 g birth weight); births and deaths registrations; hospital inpatient; opioid treatment register; cause of death; emergency department presentations; publicly funded mental health outpatients; public housing and child protection data (online supplemental files 2 and 3). Data were linked by the NSW Centre for Health Record Linkage (false positive linkages, 0.4%–0.5%).^20^

### Study population

The study population included live and still births recorded in the perinatal, birth registration and/or hospital data in NSW from July 2001 to December 2019 (N=1 895 850) (online supplemental file 4). Mother–child dyads were linked using the perinatal and birth registration data. We restricted the study population to children born from January 2008 to December 2017 (N=990 631), so the availability of all data sources aligned with the gestation to second birthday period for all children (online supplementals file 3).

### Outcomes

The primary outcome was maternal substance use problems during the first 1000 days recorded in ≥1 of the six data sources with substance use information (online supplemental file 5). Substance use problems were categorised into alcohol use and other drug use problems, and further disaggregated by drug type where available (maternal data sources only). Maternal substance use problems included substance use-related contacts with tertiary level health services for the child (hospital) or mother (hospital, emergency department, mental health outpatient), mother or child substance use/in utero exposure-related deaths, mother receipt of opioid agonist treatment (OAT) and child protection reports/assessments of risk or actual harm related to carer drug and/or alcohol use. Substance use-related health system contacts and deaths were identified using the International Classification of Diseases and Related Health Problems, 10th Revision, Australian Modification (ICD-10-AM) or the Systemized Nomenclature of Medicine Clinical Terminology (SNOMED) system codes (onlinesupplemental files 6  6a). We selected ICD-10-AM and SNOMED codes from published literature, government reports, and a syndicated terminology server (Ontoserver) for SNOMED (online supplemental file 7), that related to the use of illicit substances or alcohol, misuse of prescription medicine, use of opioid-agonist treatment, and misuse of organic compounds, solvents or substances that produce psychoactive effects. We ascertained maternal OAT records from the opioid treatment registry.

Measures from child protection data comprised child concern reports and field assessments of suspected or actual risk of harm due to drug and/or alcohol use, that were reported by health professionals, police, educators and others in the community. This data source does not differentiate between maternal and other carers; however, there is evidence the majority of reports relate to mothers.^21 22^ Henceforth, we include this in the maternal substance use problems estimate indicators from all six data sources, while also stratifying results by child protection records and maternal and child health and death data sources.

#### Outcome ascertainment periods

We ascertained outcomes during the first 1000 days of children’s lives, defined as the conception date (child’s birth date minus gestational age, plus 14 days)^23^ until the child’s second birthday. We also ascertained outcomes for (1) the prenatal period, commencing from the conception date until <28 days of age, as diagnoses at birth (eg, NAS) indicate substance use during pregnancy and (2) the early-life period, commencing 28 days of age until the child’s second birthday (online supplemental file 8).

### Sociodemographic and health characteristics of mothers and children at birth

We describe the sociodemographic and health characteristics of children and mothers (at birth) who did and did not have substance use recorded in any data source, any health and death data source, or child protection records alone (table 1, online supplemental file 9).

**Table 1:** Maternal, pregnancy and child characteristics at birth of children with and without substance use problems recorded during the first 1000 days

	Total	No maternal substance use (any data source)	Maternal substance use in ≥1 data sources		
			Child protection data*	Health +/or death data	Any data source
Total, n (%) (denominator for columns)	970 470 (100)	937 823 (100)	26 045 (100)	13 975 (100)	32 647 (100)
Child characteristics at birth					
Female sex, n (%)	472 128 (48.6)	456 167 (48.6)	12 787 (49.1)	6772 (48.5)	15 961 (48.9)
Aboriginal and/or Torres Strait Islander, n (%)†	67 186 (6.9)	54 930 (5.9)	10 355 (39.8)	4959 (35.5)	12 256 (37.5)
Birth weight (kg), mean (SD)	3.4 (0.6)	3.4 (0.6)	3.1 (0.6)	2.9 (0.7)	3.1 (6.7)
Median (IQR)	3.4 (3.0–3.7)	3.4 (3.0–3.7)	3.1 (2.7–3.5)	3.0 (2.6–3.4)	3.1 (2.7–3.5)
Low birth weight (<2.5 kg), n (%)	61 030 (6.3)	55 734 (5.9)	4027 (15.5)	2947 (21.1)	5296 (16.2)
Gestational age (weeks), mean (SD)	38.8 (2.2)	38.8 (2.1)	38.2 (2.4)	37.8 (3)	38.2 (2.6)
Median (IQR)	39 (38–40)	39 (38–40)	39 (37–40)	38 (37–40)	39 (37–40)
Preterm (<37 weeks), n (%)	72 901 (7.5)	67 790 (7.2)	3909 (15.0)	2753 (19.7)	5111 (15.7)
Small for gestational age, n (%)	96 420 (9.9)	90 125 (9.6)	4974 (19.1)	3091 (22.1)	6295 (19.3)
Admitted to neonatal ICU or special care nursery,‡ n (%)	113 168 (14.6)	104 803 (14.0)	6567 (30.4)	4827 (42.4)	8365 (31.1)
Mother characteristics at birth					
Mother’s age (years) at birth of cohort child,§ mean (SD)	30.4 (5.6)	30.6 (5.5)	26.5 (6.6)	27.8 (6.4)	26.8 (6.6)
Median (IQR)	31 (27–34)	31 (27–34)	26 (21–31)	27 (23–33)	26 (21–32)
Mothers age at delivery (years)					
<20	27 512 (2.8)	22 726 (2.4)	4150 (15.9)	1382 (9.9)	4786 (14.7)
20–24	120 840 (12.5)	112 028 (11.9)	7136 (27.4)	3401 (24.3)	8812 (27.0)
25–29	260 485 (26.8)	252 548 (26.9)	6255 (24.0)	3672 (26.3)	7937 (24.3)
30–34	328 250 (33.8)	321 960 (34.3)	4833 (18.6)	3121 (22.3)	6290 (19.2)
35+	233 370 (24.0)	228 548 (24.4)	3671 (14.1)	2399 (17.2)	4822 (14.8)
Mother’s country/region of birth, n (%)					
Australia	634 493 (65.4)	604 849 (64.5)	23 798 (91.4)	12 671 (90.7)	29 644 (90.8)
New Zealand/Pacific	34 884 (3.6)	33 727 (3.6)	911 (3.5)	460 (3.3)	1157 (3.5)
Europe	51 520 (5.3)	51 020 (5.4)	359 (1.4)	243 (1.7)	500 (1.5)
Africa and the Middle East	55 734 (5.7)	55 366 (5.9)	255 (1.0)	164 (1.2)	368 (1.1)
South-East Asia	54 175 (5.6)	53 680 (5.7)	377 (1.4)	227 (1.6)	495 (1.5)
North-East Asia	56 943 (5.9)	56 783 (6.1)	114 (0.4)	64 (0.5)	160 (0.5)
Southern and Central Asia	62 139 (6.4)	62 003 (6.6)	92 (0.4)	51 (0.4)	136 (0.4)
Americas	17 387 (1.8)	17 224 (1.8)	119 (0.5)	80 (0.6)	163 (0.5)
Aboriginal and/or Torres Strait Islander, n (%)	51 003 (5.3)	41 914 (4.5)	7796 (29.9)	3674 (26.3)	9089 (27.8)
Mother married/de facto,¶ n (%)	808 753 (83.3)	795 798 (84.9)	9845 (37.8)	5538 (39.6)	12 955 (39.7)
Private health insurance/patient,** n (%)	205 866 (21.2)	205 262 (21.9)	300 (1.2)	343 (2.5)	604 (1.9)
Area of residence,†† n (%)					
Lived in major city	751 815 (77.5)	732 726 (78.1)	15 059 (57.8)	8719 (62.4)	19 089 (58.5)
Lived in inner regional area	150 122 (15.5)	140 645 (15.0)	7678 (29.5)	3698 (26.5)	9477 (29.0)
Lived in outer regional area	47 059 (4.8)	43 823 (4.7)	2630 (10.1)	1188 (8.5)	3236 (9.9)
Lived in remote/very remote area	4759 (0.5)	4315 (0.5)	385 (1.5)	143 (1.0)	444 (1.4)
Area-level disadvantage,‡‡ n (%)					
Quintile 1 (most disadvantaged)	213 028 (22.0)	200 803 (21.4)	9893 (38.0)	5102 (36.5)	12 225 (37.5)
Quintile 2	177 648 (18.3)	169 072 (18.0)	6982 (26.8)	3417 (24.5)	8576 (26.3)
Quintile 3	211 875 (21.8)	205 204 (21.9)	5383 (20.7)	2785 (19.9)	6671 (20.4)
Quintile 4	148 809 (15.3)	145 901 (15.6)	2235 (8.6)	1415 (10.1)	2908 (8.9)
Quintile 5 (least disadvantaged)	218 740 (22.5)	216 486 (23.1)	1545 (5.9)	1245 (8.9)	2254 (6.9)
History of contact with public housing system,§§ n (%)	45 315 (4.7)	34 151 (3.6)	9531 (36.6)	4810 (34.4)	11 164 (34.2)
Pregnancy characteristics relating to child’s birth					
Number of previous pregnancies, n (%)					
Mother had no prior births	420 220 (43.3)	409 737 (43.7)	7975 (30.6)	4511 (32.3)	10 483 (32.1)
Mother had one prior birth	326 902 (33.7)	318 947 (34.0)	6201 (23.8)	3434 (24.6)	7955 (24.4)
Mother had≥2 prior births	222 591 (22.9)	208 421 (22.2)	11 842 (45.5)	6005 (43.0)	14 170 (43.4)
Number of previous pregnancies, mean (SD)	0.9 (1.2)	1.7 (1.8)	1.8 (1.9)	1.7 (1.8)	1.7 (1.8)
Median (IQR)	1 (0–1)	1 (0–1)	1 (0–3)	1 (0–3)	1 (0–3)
Mother did not receive antenatal care in first 20 weeks of pregnancy	102 125 (10.5)	94 581 (10.1)	6256 (24.0)	3365 (24.1)	7544 (23.1)
Mother smoked during pregnancy,¶¶ n (%)	34 615 (10.2)	27 500 (8.4)	6053 (65.2)	3096 (68.9)	7115 (63.2)

Number of missing or not recorded values: sex missing 16; Child Aboriginal or Torres Strait Islander missing 18; low birth weight missing 770; small for gestation age missing 902; Admitted to neonatal ICU missing 554; Mother’s Aboriginal and/or Torres Strait Islander missing 129, not recorded 21 722; Mother’s country/region of birth missing 3195; Married or in de facto partnership missing 23 997; private health insurance/patient not recorded 58; Mother’s remoteness area of residence missing <5, not recorded 16 713; area-level disadvantage not recorded 370; number of previous pregnancies missing 757; antenatal care in the first 20 weeks of pregnancy missing 11 675; Smoking during pregnancy missing 327.

* The child protection data source includes maternal and other carer substance use.

† Defined as child or parent identified as Aboriginal on any of the birth records (ie, perinatal data collection, birth registration or hospital record).

‡ Numbers and percents calculated for children born from 2008 to 2015 (N=776 273) when this variable was collected in the Perinatal data collection, see online supplemental file 99 for more details.

§ The month and year of birth for the mother was obtained from the birth registration, or the Perinatal Data Collection if the birth registration was unavailable, and used to calculate maternal age at childbirth, in years.

¶ As recorded on the hospital birth record.

** As recorded on the hospital birth record for the mother or child.

†† Based on the Accessibility/Remoteness Index of Australia for the Statistical Local Area of residence at the child’s birth.

‡‡ Based on the Index of Relative Socio-economic Advantage and Disadvantage for the Statistical Local Area of residence at the child’s birth.

§§ Mother is living in public housing or is on the tenancy waitlist at any time 2 years prior to the child’s birth.

¶¶ Variable derived from variables recorded in Perinatal Data Collection, indicates if the mother smoked at any time point during pregnancy.

ICU, intensive care unit.

### Analysis methods

**Figure 1:**
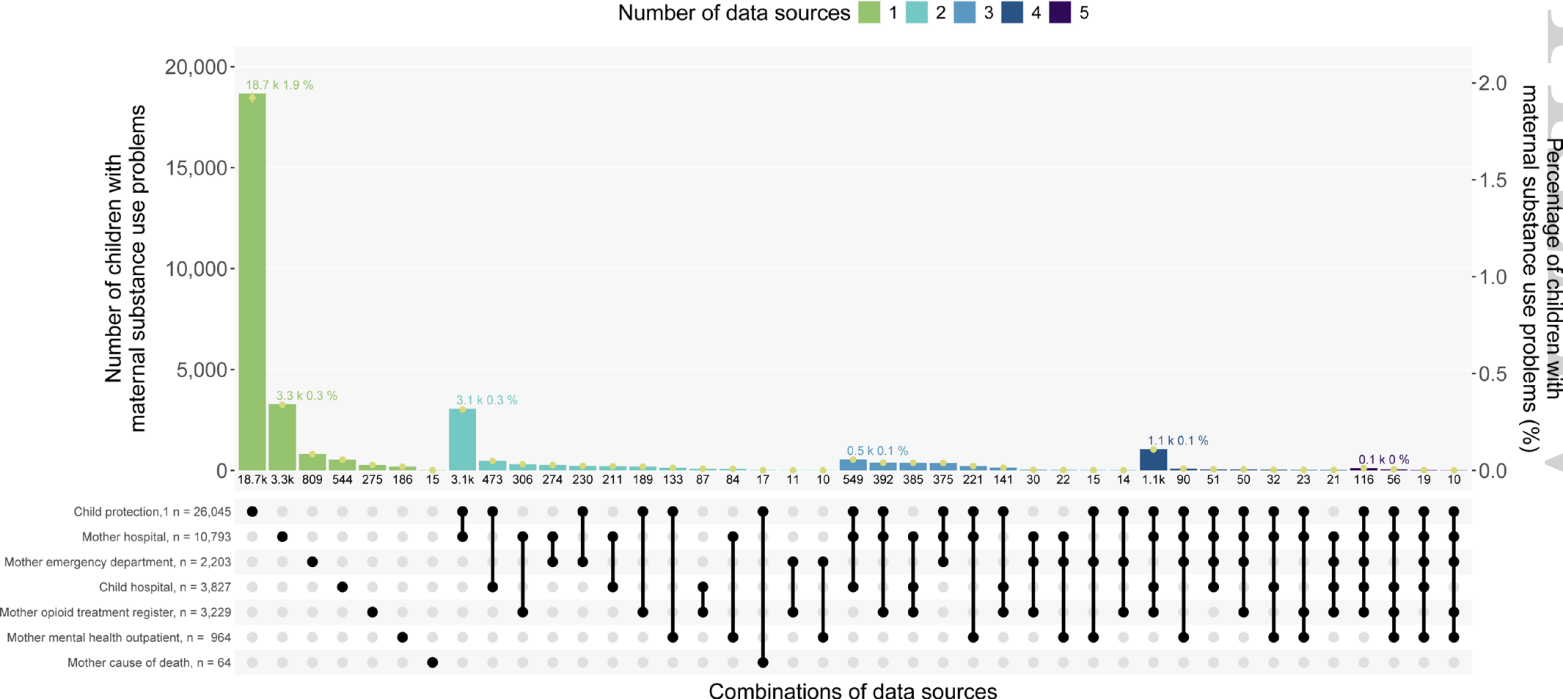
Most common combinations of data sources with maternal substance use problems recorded, for children born in NSW from 2008 to 2017 (n=970 470). 122 children were not represented on the upset plot: 93 children were not included as we excluded combinations of data sources with less than 10 children, and 19 children were not included as they had a record of maternal substance use in 6 data sources. All values for numerators, denominators and 95% CIs provided in online supplemental file 9. ^1^The child protection data source includes maternal and other carer substance use. NSW, New South Wales.

We calculated the number and percent of children born 2008–2017 with outcomes recorded in: each data source; any data source; any health/death data source (tables1  2); and the 34 most common data source combinations (figure 1). We calculated the number and percent of children with (1) records of different substance use types (where recorded) and (2) maternal substance use problems among birth-year cohorts.

**Table 2:** Number and percentage of children with maternal substance use problems in the first 1000 days, ascertained from six data sources, for children born in NSW from 2008 to 2017 (N=970 470)

Data source	Single data sources						Combined data sources	
	Cause of death*	Mental health outpatients	Opioid treatment register	Emergency department	Hospital	Child protection†	Health +/or cause of death‡	Any data source‡
Substance type	n (%)§	n (%)§	n (%)§	n (%)§	n (%)§	n (%)§	n (%)§	n (%)§
Maternal data sources (5 data sources)								
Any substance¶	<64 (<0.1)	964 (0.1)	3229 (0.3)	2206 (0.2)	10 793 (1.1)	–	–	12 956 (1.3)
Alcohol	–	511 (<0.1)	–	1431 (0.1)	3046 (0.3)	–	–	4246 (0.4)
Other drug**	–	473 (<0.1)	3229 (0.3)	817 (0.1)	8985 (0.9)	–	–	10 126 (1.0)
Cannabis	–	107 (<0.1)	–	43 (<0.1)	4435 (0.5)	–	–	4505 (0.5)
Opioid or opioid treatment	–	30 (<0.1)	3229 (0.3)	144 (<0.1)	3063 (0.3)	–	–	4011 (0.4)
Stimulant	–	102 (<0.1)	–	56 (<0.1)	2117 (0.2)	–	–	2182 (0.2)
Sedatives or other††	–	6 (<0.1)	–	10 (<0.1)	549 (0.1)	–	–	572 (0.1)
Unspecified or multiple‡‡	–	342 (<0.1)	–	618 (0.1)	1858 (0.2)	–	–	2576 (0.3)
Child data sources (3 data sources)								
Any substance¶	<5 (<0.1)	–	–	–	3827 (0.4)	26 045 (2.7)	–	27 327 (2.8)
Alcohol	–	–	–	–	62 (<0.1)	10 702 (1.1)	–	10 726 (1.1)
Other drug**	–	–	–	–	3787 (0.4)	18 152 (1.9)	–	19 559 (2.0)
Child and maternal data sources combined (6 data sources)‡								
Any substance¶	64 (<0.1)	964 (0.1)	3229 (0.3)	2203 (0.2)	12 067 (1.2)	26 045 (2.7)	13 975 (1.4)	32 647 (3.4)
Alcohol	–	511 (<0.1)	–	1431 (0.1)	3074 (0.3)	10 702 (1.1)	11 175 (1.2)	13 637 (1.4)
Other drug**	–	473 (<0.1)	3229 (0.3)	814 (0.1)	10 282 (1.1)	18 152 (1.9)	4272 (0.4)	23 485 (2.4)

* For cause of death data, reporting is limited to the outcome ‘any substance use’ due to low numbers in specific substance types.

† The child protection data source includes maternal and other carer substance use.

‡ The outcomes are not mutually exclusive across data sources or substance types; therefore, numbers in rows/columns do not sum to numbers reported in ‘All records and data sources’ (rows) or the ‘Any substance use’ outcome (columns).

§ 95% CIs are provided in online supplemental file 10.

¶ Any substance use includes the use of alcohol and/or other drug use.

** Other drug use includes the use of drugs other than alcohol and tobacco, including illicit drugs, misuse of prescription drugs and use of opioid agonist treatment.

†† 'Sedatives or other’ includes the use of sedatives, hallucinogens, sedatives, solvents or organic compounds, which were aggregated due to small cell sizes in individual substance categories.

‡‡ 'Unspecified or multiple substance use’ includes ICD10AM codes that indicate multiple drug use or do not specify the type of drug or psychoactive substance.

ICD10AM, International Classification of Diseases and Related Health Problems, 10th Revision, Australian Modification; NSW, New South Wales.

#### Sensitivity analyses

We conducted sensitivity analyses where the maternal substance use problems outcome: (1) included additional ICD-10-AM and SNOMED codes that may indicate either the misuse of, or accidental overdose or poisoning from prescription medicines (online supplemental files 6 and 6a) or (2) excluded the receipt of OAT. While OAT is a harm minimisation strategy protective for pregnant women and the developing fetus/infant, exposure to OAT in utero may impact the child’s health (eg, NAS).

## Results

There were 990 631 children born in NSW between 1 January 2008 and 31 December 2017. After excluding 20 150 children who were <20 weeks gestation or <400 g birth weight, and 21 children with a death record prior to birth, 970 470 children and 625 856 mothers were included (online supplemental file 4). The number of children born each year was relatively constant, with 96 288 in 2008 and 95 725 in 2017 (mean: 97 000 per year). In total, 48.6% of children were female, 6.9% were Aboriginal and/or Torres Strait Islander, 77.5% were born to mothers residing in major cities and the mean maternal age at birth was 30.4 years (SD 5.6 years) (table 1).

## Prevalence of maternal substance use problems ascertained from mother/child records in ≥1 data sources

We found 32 647 (3.4%) children had ≥1 mother and/or child records of maternal substance use problems during the first 1000 days in ≥1 of the six data sources, and no substance use records for 937 823 children (96.6%) (table 2, online supplemental file 10). Figure 1 shows that 23 784 children (2.4%) had maternal substance use problems recorded in only one data source and 8863 children (0.9%) in ≥2 data sources (figure 1, online supplemental file 11). There were child protection records of maternal and other carer substance use for 26 045 children (2.7%), including 18 672 (1.9%) children with substance use only recorded in the child protection data. 13 975 children (1.4%) had maternal substance use problems ascertained from child and/or mother hospital and death records (table 2), including 3283 children with substance use only in maternal hospital records and 544 only in child hospital records (figure 1). An additional 1310 children had maternal substance use problems recorded in data sources other than hospital or child protection (online supplemental file 11).

### Prevalence of maternal substance use problems by drug type

Using child and/or mother data sources, there were records indicating maternal alcohol use for 13 637 children (1.4%) and other drug use for 23 485 children (2.4%) (table 2). Maternal data sources provided additional details on drug use types. From mother health/death records, there were records indicating maternal alcohol use for 4246 children (0.4%) and other drug use for 10 126 children (1.0%). Cannabis was the most common type of other drug use ascertained (4505 children, 0.5%), followed by opioids and OAT (4011 children, 0.4%). ‘Unspecified or multiple drug use’ was recorded in mothers’ health records for 2576 (0.3%) children. Single substance use was recorded more frequently than multiple use: 9574 children had 1 and 3401 children ≥2 substance/s recorded in their mother’s records (further details on the dual/multiple substance use are reported in online supplemental files 12 and 13).

### Annual prevalence of maternal substance use problems, 2008–2017

**Figure 2:**
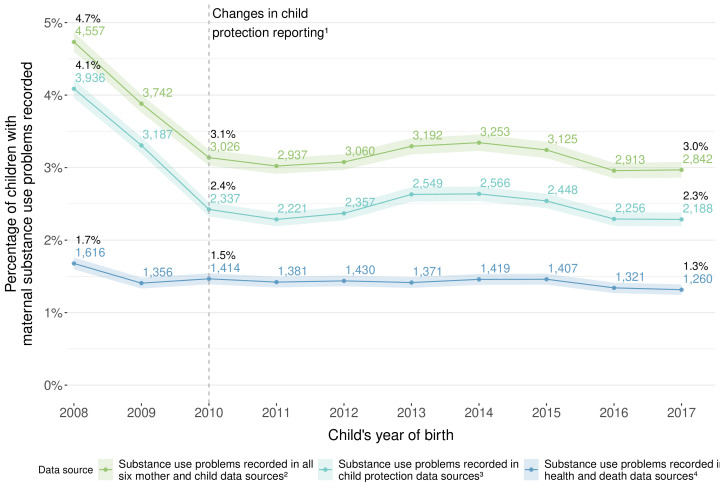
Children with maternal substance use problems in the first 1000 days of life, ascertained from different combinations of administrative data sources, by child’s year of birth. Total denominator for each birth year ranged from 95 725 to 99 458, mean 97 047 (SD 1134), all denominators, numerators, percentages and 95% CIs provided in online supplemental file 14. ^1^The Keep Them Safe Initiative and related changes to mandatory reporting guidelines in 2010 in NSW reduced the numbers of child protection reports across all age groups from 2010. ^2^Substance use ascertained from maternal hospital, cause of death, mental health outpatients, opioid treatment registry and emergency department records and child hospital, cause of death and child protection records; ^3^The child protection data source includes maternal and other carer substance use; ^4^Substance use ascertained from maternal hospital, cause of death, mental health outpatients, opioid treatment registry, and emergency department records, and child hospital and cause of death records.

The prevalence of maternal substance use problems ascertained from ≥1 health/death data sources in the first 1000 days was relatively stable, ranging from 1.7% in 2008 to 1.3% in 2017 (figure 2, online supplemental file 14). Consistent with the substantial decrease in all child protection reports following system-wide changes to reporting and screening in 2010,^24^ there was a corresponding decrease in reports related to maternal and other carer substance use from 4.7% in 2008 to 3.1% in 2010 and 3.0% in 2017. On average, each year during the study period, approximately 3400 children had ≥1 record of maternal substance use problems, 1400 alcohol use and 2400 other drug use.

### Prevalence of maternal substance use problems ascertained during the prenatal and early-life periods

**Figure 3:**
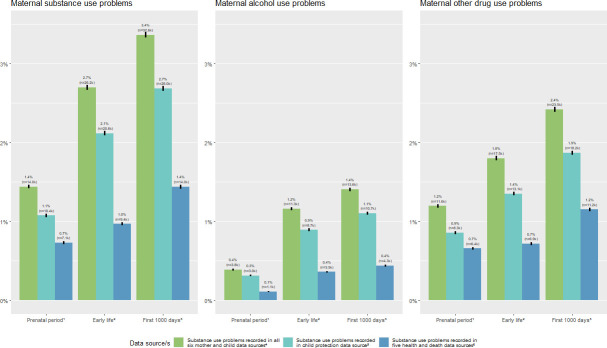
Maternal substance use problems during the prenatal period, early life and first 1000 days, among 970 470 children born in New South Wales, from 2008 to 2017. ^1^Prenatal period is defined as conception until 27 days postbirth; ^2^Early life is defined as 28 days postbirth until 2 years postbirth; ^3^Frist 1000 days are defined as conception until 2 years postbirth; ^4^Substance use ascertained from maternal hospital, cause of death, mental health outpatients, opioid treatment registry and emergency department records, and child hospital, cause of death and child protection records; ^5^The child protection data source includes maternal and other carer substance use; ^6^Substance use ascertained from maternal hospital, cause of death, mental health outpatients, opioid treatment registry, and emergency department records, and child hospital and cause of death records.

In the prenatal period, 13 987 (1.4%) children had records of maternal substance use problems, including 3776 (0.4%) with maternal alcohol use and 11 605 (1.2%) other drug use (figure 3, online supplemental file 15). In the early-life period (ie, 28 days to 2nd birthday), 26 198 (2.7%) children had records of maternal substance use problems, including 11 262 (1.2%) with alcohol use and 17 479 (1.8%) other drug use.

### Sociodemographic and health profile of children and mothers, according to maternal substance use problems

Adverse pregnancy and birth characteristics were more common among children with maternal substance use problems recorded than without, including maternal smoking during pregnancy, low birth weight, preterm birth and admission to neonatal intensive/special care units (table 1). Indicators of socioeconomic disadvantage were more common among children with than without records of maternal substance use problems, including younger maternal age at birth (eg, <20 years: 14.7% vs 2.4%), living in more disadvantaged areas (eg, most disadvantaged: 37.5% vs 21.4%), single mother at birth (60.3% vs 15.1%) and living in or being on the wait list for public housing (34.2% vs 3.6%). Aboriginal and Torres Strait Islander children and mothers were over-represented in the maternal substance use problems group (Aboriginal and/or Torres Strait Islander child 37.5% vs 5.9%).

### Sensitivity analyses

When we included diagnosis codes that potentially indicated misuse of, or accidental overdose/poisoning from, prescription medicines, 236 children were added to the maternal substance use problems group, with the prevalence remaining 3.4% (online supplemental file 16).

Almost all children (91.5%) with a mother on the opioid treatment register also had a record of maternal substance use problems in another data source (figure 1). When we excluded OAT from the outcome, the prevalence of maternal substance use problems reduced from 3.36% to 3.34%, representing 275 fewer children (online supplemental file 17).

## Discussion

Our study of almost one million Australian children showed that more than 3 in every 100 children had a record of maternal substance use problems during their first 1000 days of life. This included >1 in 100 children with prenatal substance use exposure and almost 3 in 100 children with maternal substance use problems recorded from 28 days to 2 years of age. More than 1 in 100 children had a record of maternal alcohol use problems and >2 in 100 children had a record of other drug use problems. All indicators of health and social disadvantage at birth were more common among children with records of maternal substance use problems, compared with other children.

Adding child protection data to health and death data more than doubled our prevalence estimates, from 1.4% to 3.4%. While we consider these child protection measures to provide a useful additional indicator of maternal substance use problems, in some cases, they may represent suspected harm relating to the substance use of a non-maternal carer. Nevertheless, children with exposure to family substance use are also at potential risk of harm during early life and important targets of support services. After child protection data, the next highest numbers of children with maternal substance use problems were ascertained from mother, then child hospital records, which include near-universal health system contacts at birth. Our prevalence estimates from mother/child hospital records during pregnancy and birth were comparable to previous studies using whole-of-population mother/child hospital data conducted in Australia (estimates ranging from 0.1% to 0.5%)^810 11 25^,^30^ and other high-income countries (estimates ranging from 0.3% to 1.1%)^11^,^1431 32^ over the last two decades. We followed children beyond birth, revealing that almost 3 in 100 had maternal substance use problems recorded between 28 days and their second birthday.

While we found fewer children had records of maternal alcohol use problems than other drug use problems, another whole-of-population cohort study in Canada reported the opposite (during-pregnancy alcohol use 11.7% vs illicit drug use 3.6%) when using prenatal screening data in addition to hospital data.^18^ This difference likely reflects the varied levels of substance use measured in different healthcare contexts and data. Our study using tertiary healthcare and child protection data likely captured visible and potentially harmful alcohol consumption, relevant to planning substance use in pregnancy and related service supports. In contrast, it is likely self-reported substance use in universal prenatal screening also captures lower level and/or intermittent alcohol consumption, similar to general population surveys such as the Australian National Drug Strategy Household Survey,^6^ which estimated that 15% of the pregnant subset of women in the survey consumed any alcohol when they knew they were pregnant. It is also documented that while both drug and alcohol use disorder diagnoses are underreported in hospital records, alcohol use disorder diagnoses are underreported to an even greater extent than other drug disorders.^33^

A strength of our study was the use of comprehensive health, death and child protection data sources spanning 10 years. Using administrative data enabled us to assemble birth cohorts highly representative of our target population, avoiding selection issues common in primary data collection studies. However, using administrative data limited the ascertainment of substance use to what was observed, reported, treated and/or recorded in health system or child protection contacts. In our study, we ascertained substance use recorded in tertiary healthcare contacts; from records potentially indicating chronic harmful use (eg, diagnoses of drug dependence), acute harmful use (eg, emergency presentations for intoxication), as well as the receipt of substance use treatment (mental health appointments and OAT). We cannot determine how well mothers were supported by their healthcare professionals and other services from such data. If mothers were supported, could function as parents and were not breastfeeding or around their child while using/affected, the risks to the child may have been low. However, we have likely captured more harmful substance use from most data sources that is relevant to the planning of substance use in pregnancy services.

Other data, such as data from antenatal screening and primary care settings, may capture information on lower levels of substance use, as well as more universal health system contacts. In our study, only birth admissions in hospital data represented universal service contacts for all mothers and children. All data sources were collected for statewide services, but most were limited to those mothers and/or children in contact with a service (eg, emergency department or publicly funded mental health services). However, the additional children ascertained from child protection data may represent children who were visible to and reported from other health and human services. We describe other considerations related to data source-specific coding and ascertainment in online supplemental file 18.

Another important consideration when using administrative data is that different population groups may be screened or surveilled differently for maternal substance use. Studies suggest women who present late to antenatal care, are younger^34 35^ or among racial and ethnic minority populations^35 36^ are more likely to be screened for substance use. We also found a higher proportion of younger, single mothers, who did not receive antenatal care before 20 weeks had records of maternal substance use problems. Aboriginal mothers and children were also over-represented, reflecting the historical and ongoing systemic racism, oversurveillance and intervention in the lives of Aboriginal peoples in Australia across sectors, including health, child protection and social services.^37 38^ Given substance use can be a response and coping mechanism to adversity, interpersonal and state violence—including intergenerational trauma and child removals^39^—it is critical to consider culturally safe and trauma-informed responses to maternal substance use, alongside other unmet health and social needs.^37 40^

More than 3 in 100 children had maternal substance use problems or related conditions recorded in administrative data during the critical first 1000 days of development. This period of near universal contact with the health system provides the opportune time to identify and respond to maternal and family substance use early in a child’s life to prevent adverse outcomes. By combining child protection records with health data more traditionally used for public health surveillance, we demonstrated the potential to build a more comprehensive view of maternal substance use at the population level to inform prevention responses for pregnant women, new mothers and children at the earliest opportunity. However, as substance use was ascertained from health and child protection records, our estimate (3.4%) likely represents the minimum number of families that may need support for potentially harmful substance use.

Children and families with evidence of maternal substance use problems experienced a disproportionate burden of health and social disadvantage, for example, 4 in 10 children with a record of maternal substance use problems live in the most disadvantaged areas. Pregnant women and new mothers using substances may need support from antenatal, birth and postnatal health services that is coordinated with other mainstream and Aboriginal community-controlled services, including alcohol and other drug services, mental health services, early childhood health services and other social services such as housing and child protection.

## Conclusions

More than 3 in every 100 Australian children had a record of maternal substance use problems or conditions in administrative data sources during the first 1000 days of life in this decade-long study. In addition to health and death data sources, child protection data offers public health insights into the scale of maternal substance use problems among whole-population cohorts of children. The higher burden of health and social disadvantage among children and families affected by maternal substance use during pregnancy and early life highlights the need for coordinated health and social supports from the earliest opportunity.

## Supplementary material

10.1136/jech-2024-223439 online supplemental file 1

10.1136/jech-2024-223439 online supplemental file 2

## Data Availability

No data are available.
