# Supplementary material for: Prevalence of maternal substance use problems during pregnancy and the first 2 years of life: a whole-population birth cohort of 970 470 Australian children born 2008–2017
Source: J Epidemiol Community Health. 2025 Mar 24;79(8):e223439. doi: 10.1136/jech-2024-223439 (PMC12322432; doi:10.1136/jech-2024-223439)
Supplement: online supplemental file 1 [file jech-79-8-s001.pdf]

Supplemental files to
Prevalence of maternal substance use problems during pregnancy and first two years of life: A whole-population cohort of 970,470 Australian children born 2008-2017

Contents

Supplemental file 1. RECORD statement – checklist of items that should be reported in observational studies using routinely collected health data 3

Supplemental file 2. Data sources included in the analysis, with technical names and the years data sources are available 7

Supplemental file 3. Study population and years data available for each birth cohort 8

Supplemental file 4. Study population assembled from linked data sources 9

Supplemental file 5: Data sources and variables/codes used to ascertain maternal substance use problems during the first 1000 days of life 10

Supplemental file 6: Data used to ascertain maternal substance use problems from six administrative data sources 11

Supplemental file 7. Sources used to inform the selection of ICD-10-AM and SNOMED codes 16

Supplemental file 8. Outcome ascertainment periods 17

Supplemental file 9. Supporting information for Table 1. Maternal, pregnancy and child characteristics at birth of children with and without maternal substance use problems recorded during the first 1000 days 18

Supplemental file 10. Number, percentage, and 95% CIS of children with maternal substance use problems in the first 1000 days, ascertained from six data sources, for children born in NSW from 2008-2017 21

Supplemental file 11. Data for Figure 1. Number of children with maternal substance use problems in different combinations of data sources 22

Supplemental file 12. Most common combinations of substance types recorded in maternal data sources, among children born in NSW from 2008-2017 (n=970,470) 25

Supplemental file 13. Data to accompany Supplemental file 12. Number of children with maternal substance use problems in different combinations of substance types recorded in maternal records 26

Supplemental file 14. Data to accompany Figure 2. Children with maternal substance use problems in the first 1000 days of life, ascertained from different combinations of data sources, by child's year of birth 28

Supplemental file 15. Data to accompany Figure 3. Number and percentage of children born in NSW from 2008-2017 (N=970,470) with maternal substance use problems records during three ascertainment periods and six administrative data sources for children and mothers 29

Supplemental file 16. Sensitivity analysis: Number and percentage of children with maternal substance use problems in the first 1000 days, ascertained from six data sources, for children born in NSW from 2008-2017 (N=970,470) using additional ICD codes for substance use ascertainment from maternal hospital, mental health and cause of death records 30

Supplemental file 17. Sensitivity analysis: number and percentage of children with maternal substance use problems in the first 1000 days, ascertained from five data sources, for children born in NSW from 2008-2017 (N=970,470) 31

Supplemental file 18. Limitations of data sources used in analysis........ 32

Supplemental file 1. RECORD statement – checklist of items that should be reported in observational studies using routinely collected health data.

	Item No.	STROBE items	RECORD items	Location in manuscript
**Title and abstract**				
	1	(a) Indicate the study's design with a commonly used term in the title or the abstract (b) Provide in the abstract an informative and balanced summary of what was done and what was found	RECORD 1.1: The type of data used should be specified in the title or abstract. When possible, the name of the databases used should be included.<br><br>RECORD 1.2: If applicable, the geographic region and timeframe within which the study took place should be reported in the title or abstract.<br><br>RECORD 1.3: If linkage between databases was conducted for the study, this should be clearly stated in the title or abstract.	Title page 1. Abstract page 3.
**Introduction**				
Background rationale	2	Explain the scientific background and rationale for the investigation being reported		Introduction Paragraph 1&2
Objectives	3	State specific objectives, including any prespecified hypotheses		Introduction paragraph 3
**Methods**				
Study Design	4	Present key elements of study design early in the paper		Methods
Setting	5	Describe the setting, locations, and relevant dates, including periods of recruitment, exposure, follow-up, and data collection		Study population paragraph, Supplemental file 3-4
Participants	6	*(a) Cohort study* - Give the	RECORD 6.1: The methods of study	
		eligibility criteria, and the sources and methods of selection of participants. Describe methods of follow-up<br>*Case-control study* - Give the eligibility criteria, and the sources and methods of case ascertainment and control selection. Give the rationale for the choice of cases and controls *Cross-sectional study* - Give the eligibility criteria, and the sources and methods of selection of participants<br><br>*(b) Cohort study* - For matched studies, give matching criteria and number of exposed and unexposed	population selection (such as codes or algorithms used to identify subjects) should be listed in detail. If this is not possible, an explanation should be provided.<br><br>RECORD 6.2: Any validation studies of the codes or algorithms used to select the population should be referenced. If validation was conducted for this study and not published elsewhere, detailed methods and results should be provided.	Study population, Supplemental file 3 & 4

	Item No.	STROBE items	RECORD items	Location in manuscript
		Case-control study - For matched studies, give matching criteria and the number of controls per case	RECORD 6.3: If the study involved linkage of databases, consider use of a flow diagram or other graphical display to demonstrate the data linkage process, including the number of individuals with linked data at each stage.	
Variables	7	Clearly define all outcomes, exposures, predictors, potential confounders, and effect modifiers. Give diagnostic criteria, if applicable.	RECORD 7.1: A complete list of codes and algorithms used to classify exposures, outcomes, confounders, and effect modifiers should be provided. If these cannot be reported, an explanation should be provided.	Outcomes, Sociodemographic and health characteristics of mothers and children at birth, Supplemental file 4, 6, 6a, 8
Data sources/ measurement	8	For each variable of interest, give sources of data and details of methods of assessment (measurement).<br>Describe comparability of assessment methods if there is more than one group		Data sources and data linkage, Outcomes, Supplemental file 4, 6, 6a
Bias	9	Describe any efforts to address potential sources of bias		Outcomes, Sensitivity Analysis
Study size	10	Explain how the study size was arrived at		Study population, Supplemental file 3 & 4
Quantitative variables	11	Explain how quantitative variables were handled in the analyses. If applicable, describe which groupings were chosen, and why		Analysis methods, Outcomes
Statistical methods	12	(a) Describe all statistical methods, including those used to control for confounding (b) Describe any methods used to examine subgroups and interactions<br>(c) Explain how missing data were addressed<br>(d) *Cohort study* - If applicable, explain how loss to follow-up<br>was addressed<br>*Case-control study* - If applicable, explain how matching of cases and controls<br>was addressed<br>*Cross-sectional study* - If applicable, describe analytical methods taking account of sampling strategy		Analysis methods

	Item No.	STROBE items	RECORD items	Location in manuscript
		(e) Describe any sensitivity analyses		
Data access and cleaning methods		..	RECORD 12.1: Authors should describe the extent to which the investigators had access to the database population used to create the study population.<br><br>RECORD 12.2: Authors should provide information on the data cleaning methods used in the study.	Methods, Data source and data linkage, study population, Supplemental file 4
Linkage		..	RECORD 12.3: State whether the	
			study included person-level, institutional-level, or other data linkage across two or more databases. The methods of linkage and methods of linkage quality evaluation should be provided.	Data sources and data linkage
Participants	13	(a) Report the numbers of individuals at each stage of the study (*e.g.*, numbers potentially eligible, examined for eligibility, confirmed eligible, included in the study, completing follow-up, and analysed)<br>(b) Give reasons for nonparticipation at each stage. (c) Consider use of a flow diagram	RECORD 13.1: Describe in detail the selection of the persons included in the study (*i.e.,* study population selection) including filtering based on data quality, data availability and linkage. The selection of included persons can be described in the text and/or by means of the study flow diagram.	Study population, Supplemental file 3 & 4
Descriptive data	14	(a) Give characteristics of study participants (*e.g.*, demographic, clinical, social) and information on exposures and potential confounders<br>(b) Indicate the number of participants with missing data for each variable of interest (c) *Cohort study* - summarise follow-up time (*e.g.*, average and total amount)		Results paragraph 1, Table 1, Results: Sociodemographic and health profile of children and mothers, according to maternal substance use
Outcome data	15	*Cohort study* - Report numbers of outcome events or summary measures over time<br>*Case-control study* - Report numbers in each exposure category, or summary measures of exposure<br>*Cross-sectional study* - Report numbers of outcome events or		Table 2, Figure 1-3, Supplemental file 10-11,<br><br>Results paragraph 2-3
		summary measures		

	Item No.	STROBE items	RECORD items	Location in manuscript
Main results	16	(a) Give unadjusted estimates and, if applicable, confounder adjusted estimates and their precision (e.g., 95% confidence interval). Make clear which confounders were adjusted for and why they were included (b) Report category boundaries when continuous variables were categorized<br>(c) If relevant, consider translating estimates of relative risk into absolute risk for a meaningful time period		Table 2, Figure 1-3, Supplemental file 9-13<br>Results paragraph 2-5
Other analyses	17	Report other analyses done—e.g., analyses of subgroups and interactions, and sensitivity analyses		Supplemental file 16-17, Results: Sensitivity analyses
Key results	18	Summarise key results with reference to study objectives		Discussion paragraph 1 and 2
Limitations	19	Discuss limitations of the study, taking into account sources of potential bias or imprecision. Discuss both direction and magnitude of any potential bias	RECORD 19.1: Discuss the implications of using data that were not created or collected to answer the specific research question(s). Include discussion of misclassification bias, unmeasured confounding, missing data, and changing eligibility over time, as they pertain to the study being reported.	Discussion Paragraph 2-6
Interpretation	20	Give a cautious overall interpretation of results considering objectives, limitations, multiplicity of analyses, results from similar studies, and other relevant evidence		Discussion paragraph 7
Generalisability	21	Discuss the generalisability (external validity) of the study results		Discussion Paragraph 4, 5, 6
Funding	22	Give the source of funding and the role of the funders for the present study and, if applicable, for the original study on which the present article is based		Funding and acknowledgments
Accessibility of protocol, raw data, and programming code		..	RECORD 22.1: Authors should provide information on how to access any supplemental information such as the study protocol, raw data, or programming code.	NA

*Reference: Benchimol EI, Smeeth L, Guttmann A, Harron K, Moher D, Petersen I, Sørensen HT, von Elm E, Langan SM, the RECORD Working Committee. The REporting of studies Conducted using Observational Routinely-collected health Data (RECORD) Statement. *PLoS Medicine* 2015; in press.

Supplemental file 2. Data sources included in the analysis, with technical names and the years data sources are available

Data sources[1]	Pregnancy	Birth	Neonatal period	First 1000 days	Years data are available
**Mother's records**					
NSW Perinatal Data Collection (formerly known as the Midwives Data Collection) (Perinatal data)					2001-2019
NSW Registry of Births, Deaths, and Marriages birth registrations (Birth registrations)					2001-2019
NSW Admitted Patient Data Collection (Hospital inpatient data)					Jul 2001-2019
NSW Emergency Department Data Collection (Emergency department presentations)					2005 -2019
NSW Mental Health Ambulatory Data Collection (Mental health outpatient data)					2006-2019
Registry of Births, Deaths, and Marriages (RBDM) death registrations					2001 -2019
NSW Cause of Death Unit Record File Unit Record File (Cause of death records)					2001-2019
NSW Department of Communities and Justice, Social housing applicant file and Tenancy file (Public housing data)					2001-2019
NSW Controlled Drugs Data Collection - Opioid Treatment Program (Opioid treatment registry)					2001-2019
**Child's records**					
NSW Perinatal Data Collection (formerly known as the Midwives Data Collection) (Perinatal data)					2001-2019
NSW Registry of Births, Deaths, and Marriages birth registrations (Birth registrations)					2001-2019
NSW Admitted Patient Data Collection (Hospital inpatient data)					Jul 2001-2019
Registry of Births, Deaths, and Marriages (RBDM) death registrations (Beath registrations)					2001 -2019
NSW Cause of Death Unit Record File Unit Record File (Bause of death records)					2001-2019
NSW Department of Communities and Justice, child protection reports and investigations (Child protection data)					2004 -2019

1. More information on all data sources can be found at https://www.cherel.org.au/datasets Definitions: NSW: New South Wales

Supplemental file 3. Study population and years data available for each birth cohort

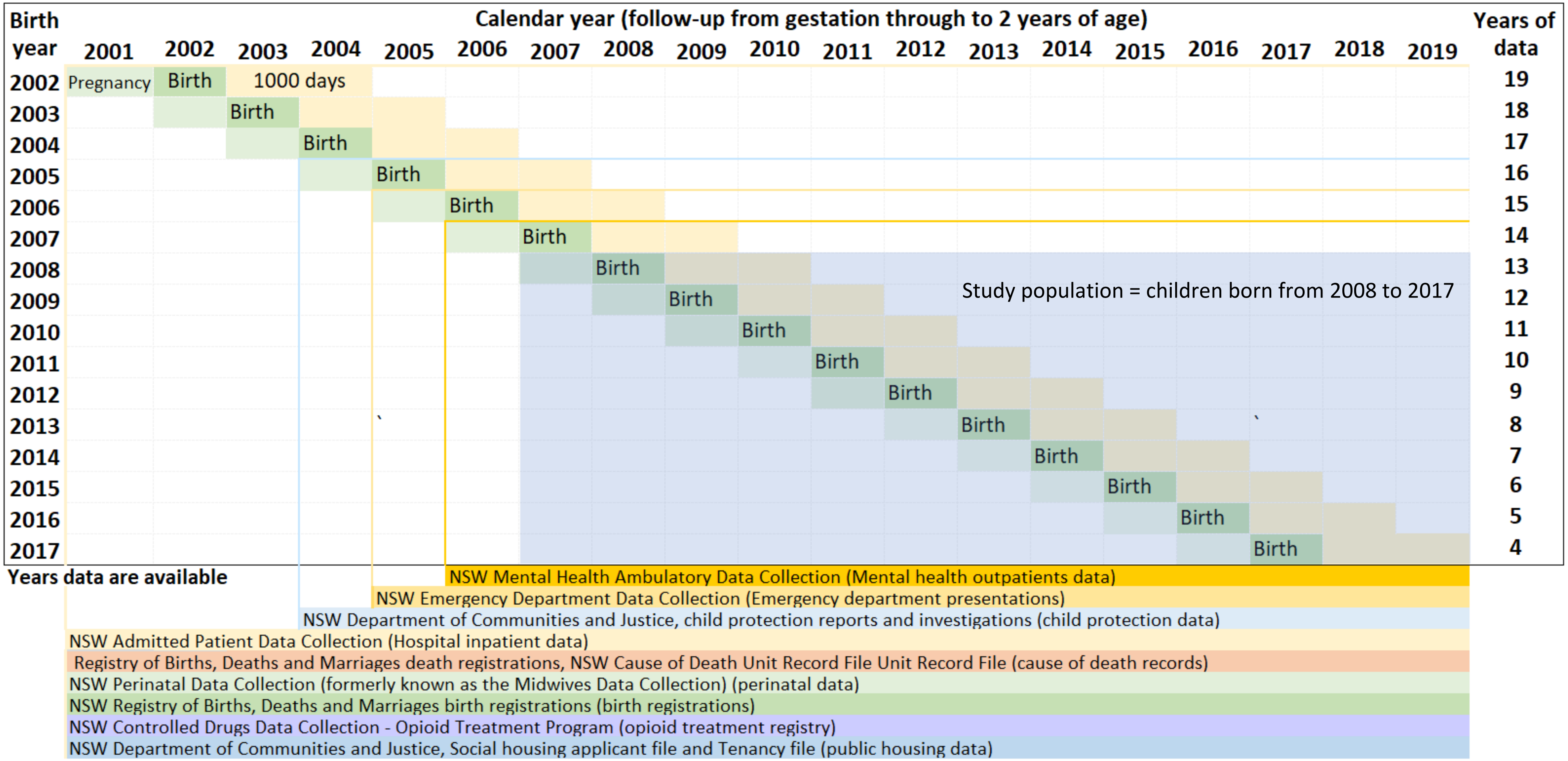

Supplemental file 4. Study population assembled from linked data sources

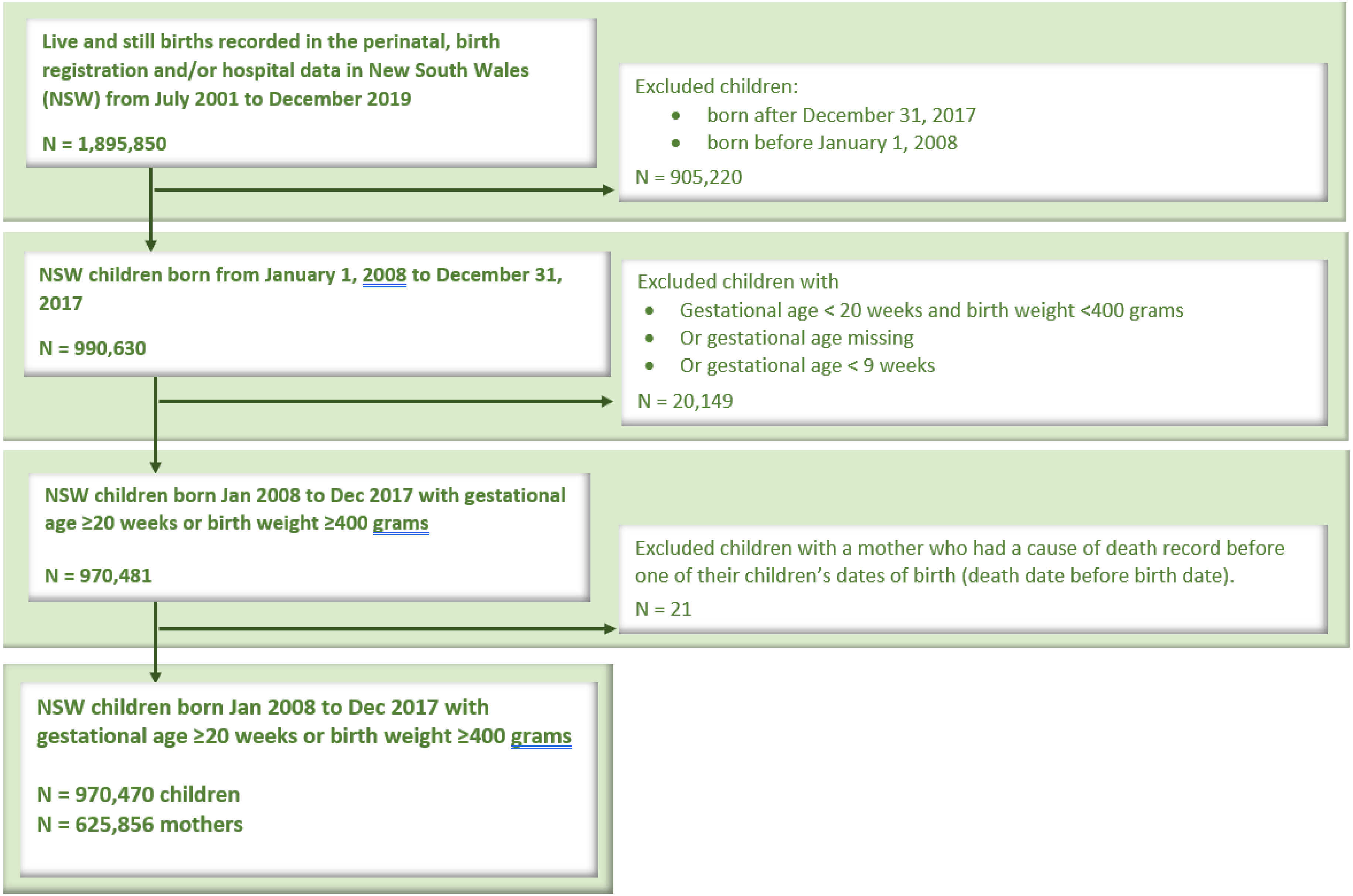

Supplemental file 5: Data sources and variables/codes used to ascertain maternal substance use problems during the first 1000 days of life

Data source	Maternal records	Child records
**Hospital data** (Admitted Patient Data Collection)	**257 ICD-10-AM codes for substance use related diagnoses** (e.g., mental health diagnoses, poisonings, and intoxication/withdrawal related to substance use, and findings of substances in blood tests)	**4 ICD-10-AM codes indicating in utero exposure to substances** (e.g., neonatal withdrawal symptoms, newborn affected by maternal use of substances, and fetal alcohol syndrome)
**Emergency Presentations** (Emergency Department Data Collection)	**257 ICD-10-AM + 534 SNOMED-CT codes for substance use related diagnoses** (e.g., mental health diagnoses, poisonings, and intoxication/withdrawal related to substance use, and findings of substance use in blood tests)	NA
**Mental health outpatient's data** (Mental Health Ambulatory Data Collection)	**257 ICD-10-AM codes for substance use related diagnoses** (e.g., mental health diagnoses related to substance use)	NA
**Cause of Death Register** (Cause of Death Unit Record File)	**257 ICD-10-AM codes for substance use related diagnoses** (e.g., mental health diagnoses, poisonings, and intoxication/withdrawal related to substance use, and findings of substances in blood tests)	**4 ICD-10-AM codes indicating in utero exposure to substances** (e.g., neonatal withdrawal symptoms, newborn affected by maternal use of substances, and fetal alcohol syndrome)
**Opioid Treatment Register** (Controlled Drugs Data Collection)	**Mother had a current authority for opioid agonist treatment**	NA
**Reports to child protection**	NA	**Harm and/or risk of harm related to carer drug or alcohol use** reported to or assessed by child protection services

Supplemental file 6: Data used to ascertain maternal substance use problems from six administrative data sources

<table>
<tr><th>Details of data used</th><th>Codes/variables (ICD-10-AM and SNOMED-CT code definitions are provided in the separate Supplemental File 6a)</th></tr>
<tr><td>Hospital records (APDC)[1]</td><td></td></tr>
<tr><td>Primary and secondary diagnoses (ICD10-AM diagnostic codes) indicating use of substances in the mother's records and children's.</td><td>Mother records:
Any substance: Combined measure of other drugs and alcohol.
• Alcohol: O35.4, Z50.2, Z72.1, R78.0, Y90-91.9, T51-51.9, X45, X65, Y15, F10-10.9
• Other drugs: Combined measure of ICD10-AM codes indicating opioids, cannabis, stimulants (cocaine, amphetamines, other stimulants), hallucinogens, solvents, sedatives and hypnotics, and drug unspecified or multiple drug:
    • Opioids: T40.0-40.3, F11- F11.9
    • Cannabis T40.7, F12-12.9
    • Stimulants: including cocaine: R78.2, T40.5, F14-14.9, amphetamines: T43.61, T43.62, and other stimulants T43.69, F15-15.99
    • Sedatives or other: Sedatives and hypnotics: F13-13.99; Volatile and organic solvents: F18-18.9, T52, T52.0, T52.1, T52.8, T52.9; and Hallucinogens: R78.3, T40.8, T40.9, F16-16.99
    • Drug unspecified or multiple: O35.5, Z50.3, Z72.2, R78, F19-19.9, T40, T40.4, T40.6

Additional codes for sensitivity analysis:
Other drugs: Combined measure as above, with the addition of codes which could indicate poisoning from a substance that may be misused, but also could be attributed to the accidental poisoning from prescription medication: T41.22, T42, T42.3, T42.4, T42.6, T42.7, T43, T43.6, T43.60, T43.8, T43.9)</td></tr>
<tr><td></td><td>Child records:
Any substance: Combined measure of other drugs and alcohol.
• Alcohol: P04.3, Q86.0
Other drugs: P96.1, P04.4</td></tr>
<tr><td>Cause of Death records[1]</td><td></td></tr>
<tr><td>Underlying Cause of Death Diagnosis Code and Contributing causes of death (ICD-10) diagnosis codes indicating use of substances in the mother's records and children's.</td><td>Mother records:
Any substance: Combined measure of other drugs and alcohol.
• Alcohol: O35.4, Z50.2, Z72.1, R78.0, Y90-91.9, T51-51.9, X45, X65, Y15, F10-10.9
• Other drugs: Combined measure of ICD10-AM codes indicating opioids, cannabis, stimulants (cocaine, amphetamines, other stimulants), hallucinogens, solvents, sedatives and hypnotics, and drug unspecified or multiple drug:
    • Opioids: T40.0-40.3, F11- F11.9
    • Cannabis T40.7, F12-12.9
    • Stimulants: including cocaine: R78.2, T40.5, F14-14.9, amphetamines: T43.61, T43.62, and other stimulants T43.69, F15-15.99</td></tr>
</table>

Details of data used	Codes/variables (ICD-10-AM and SNOMED-CT code definitions are provided in the separate Supplemental File 6a)
	• **Sedatives or other:** Sedatives and hypnotics: F13-13.99; Volatile and organic solvents: F18-18.9, T52, T52.0, T52.1, T52.8, T52.9; and Hallucinogens: R78.3, T40.8, T40.9, F16-16.99<br>• **Drug unspecified or multiple:** O35.5, Z50.3, Z72.2, R78, F19-19.9, T40, T40.4, T40.6<br><br>**Additional codes for sensitivity analysis:**<br>*Other drugs: Combined measure as above, with the addition of codes which could indicate poisoning from a substance that may be misused, but also could be attributed to the accidental poisoning from prescription medication: T41.22, T42, T42.3, T42.4, T42.6, T42.7, T43, T43.6, T43.60, T43.8, T43.9)*
	Child records:<br>Any substance: Combined measure of other drugs and alcohol.<br>• Alcohol: P04.3, Q86.0<br>• Other drugs: P96.1, P04.4
Mental health outpatients records (MHAMB) [1]	
Mental Health Diagnosis Code and additional diagnosis codes (ICD-10) indicating use of substances in the mother's records.	**Mother records:**<br>**Any substance:** Combined measure of other drugs and alcohol.<br>• **Alcohol**: O35.4, Z50.2, Z72.1, R78.0, Y90-91.9, T51-51.9, X45, X65, Y15, F10-10.9<br>• **Other drugs:** Combined measure of ICD10-AM codes indicating opioids, cannabis, stimulants (cocaine, amphetamines, other stimulants), hallucinogens, solvents, sedatives and hypnotics, and drug unspecified or multiple drug:<br>• **Opioids:** T40.0-40.3, F11- F11.9<br>• **Cannabis** T40.7, F12-12.9<br>• **Stimulants:** including cocaine: R78.2, T40.5, F14-14.9, amphetamines: T43.61, T43.62, and other stimulants T43.69, F15-15.99<br>• **Sedatives or other:** Sedatives and hypnotics: F13-13.99; Volatile and organic solvents: F18-18.9, T52, T52.0, T52.1, T52.8, T52.9; and Hallucinogens: R78.3, T40.8, T40.9, F16-16.99<br>• **Drug unspecified or multiple:** O35.5, Z50.3, Z72.2, R78, F19-19.9, T40, T40.4, T40.6<br><br>**Additional codes for sensitivity analysis:**<br>*Other drugs: Combined measure as above, with the addition of codes which could indicate poisoning from a substance that may be misused, but also could be attributed to the accidental poisoning from prescription medication: T41.22, T42, T42.3, T42.4, T42.6, T42.7, T43, T43.6, T43.60, T43.8, T43.9)*
Emergency Department Data Collection (EDDC) *[1]*	

Details of data used	Codes/variables (ICD-10-AM and SNOMED-CT code definitions are provided in the separate Supplemental File 6a)
Presenting issue, ICD-10AM and SNOMED codes indicating use of substances in the mother's records in mother's records	**Mother records:**<br>***ICD10-AM***<br>**Any substance:** Combined measure of other drugs and alcohol.<br>• **Alcohol**: O35.4, Z50.2, Z72.1, R78.0, Y90-91.9, T51-51.9, X45, X65, Y15, F10-10.9<br>• **Other drugs:** Combined measure of ICD10-AM codes indicating opioids, cannabis, stimulants (cocaine, amphetamines, other stimulants), hallucinogens, solvents, sedatives and hypnotics, and drug unspecified or multiple drug:<br>o **Opioids:** T40.0-40.3, F11- F11.9<br>o **Cannabis** T40.7, F12-12.9<br>o **Stimulants:** including cocaine: R78.2, T40.5, F14-14.9; amphetamines: T43.61, T43.62; and other stimulants T43.69, F15-15.99<br>o **Sedatives or other:** Sedatives and hypnotics: F13-13.99; Volatile and organic solvents: F18-18.9, T52, T52.0, T52.1, T52.8, T52.9; and Hallucinogens: R78.3, T40.8, T40.9, F16-16.99<br>o **Drug unspecified or multiple:** O35.5, Z50.3, Z72.2, R78, F19-19.9, T40, T40.4, T40.6<br><br>**Additional codes for sensitivity analysis:**<br>*Other drugs: Combined measure as above, with the addition of codes which could indicate poisoning from a substance that may be misused, but also could be attributed to the accidental poisoning from prescription medication: T41.22, T42, T42.3, T42.4, T42.6, T42.7, T43, T43.6, T43.60, T43.8, T43.9)*<br><br>*SNOMED CT*<br>**Any substance:** Combined measure of other drugs and alcohol.<br>• **Alcohol**: 281078001, 64297001, 15167005, 440652002, 20093000, 25702006, 66590003, 191882002, 191883007, 191891003, 228330005, 231467000, 268645007, 284591009, 304605000, 386449006, 713862009, 827094004, 1149333003, 10741871000119100, 4953006, 6749002, 7052005, 7200002, 8635005, 18653004, 21000000, 25966003, 29212009, 32553006, 34938008, 41083005, 42344001, 53936005, 57346004, 61144001, 67426006, 78524005, 79578000, 82047000, 82782008, 85561006, 87460008, 89507002, 95906008, 160592001, 169942003, 191475009, 191476005, 191477001, 191478006, 191480000, 191802004, 191804003, 191805002, 191811004, 191812006, 192206005, 192207001, 192208006, 192209003, 192210008, 192211007, 192212000, 192213005, 192214004, 192215003, 192216002, 207273009, 212806006, 212807002, 212808007, 212809004, 212811008, 212813006, 212814000, 212815004, 212816003, 212817007, 212818002, 212819005, 212820004, 213687005, 216633005, 216635003, 216636002, 216640006, 216645001, 216648004, 216651006, 221843007, 221844001, 221845000, 221846004, 221847008, 221848003, 221849006, 221850006, 221851005, 221852003, 222103001, 222104007, 222105008, 87106005, 222106009, 222107000, 222108005, 222110007, 222111006, 222112004, 222113009, 222114003, 222702003, 222703008, 222704002, 222705001, 222706000, 222707009, 222708004, 222709007, 222710002, 222711003, 222713000, 223333005, 223334004, 223335003, 223336002, 223337006, 223338001, 223339009, 223340006, 223341005, 223342003, 223343008, 223344002, 223345001, 223346000, 223347009, 223348004, 223349007, 228310006, 228315001, 228316000, 228317009, 228322009, 228323004,

Details of data used	Codes/variables (ICD-10-AM and SNOMED-CT code definitions are provided in the separate Supplemental File 6a)
	228341007, 228350009, 228351008, 228353006, 228354000, 228357007, 230800004, 242263000, 242265007, 268639004, 268683008, 268684002, 268685001, 269765000, 274776000, 278363000, 287166006, 300939009, 304606004, 308742005, 315226008, 361267005, 441685000, 442669008, 442764005, 442766007, 713583005, 714829008, 10755041000119100, 288021000119107, 288041000119101, 219006, 24165007, 62213004, 28045007, 35637008, 38670004, 53041004, 53527002, 86933000, 102612005, 102897001, 135827004, 160573003, 160581002, 160593006, 160599005, 163184002, 183098002, 183486001, 191479003, 191481001, 191482008, 191803009, 191807005, 191809008, 191814007, 191815008, 191885000, 212810009, 212812001, 216632000, 216634004, 216637006, 216638001, 216639009, 216643008, 216644002, 216646000, 216649007, 216650007, 216652004, 216653009, 221842002, 228273003, 228281002, 228312003, 228313008, 228326007, 228358002, 228364009, 231464007, 231465008, 274257003, 292880007, 294420000, 302237007, 307730003, 311492009, 316322002, 316494009, 365967005, 365973006, 408945004, 408946003, 408947007, 408948002, 412198003, 413473000, 415685003, 417096006, 417633001, 419442005, 419572002, 420140004, 427013000, 429501006, 429775004, 431260004, 445628007;<br>• **Other drugs:** Combined measure of SNOMED CT codes indicating opioids, cannabis, stimulants (cocaine, amphetamines, other stimulants), hallucinogens, solvents, sedatives and hypnotics, and drug unspecified or multiple drug:<br>o **Opioids or opioid treatment:** 5602001, 52866005, 75544000, 191820008, 191909007, 191912005, 191913000, 231477003, 231478008, 231479000, 231480002, 310653000, 428819003, 429512006, 792901003, 792902005, 1255015004, 1081000119105, 145121000119106, 1515351000168100, 1640611000168100, 1640661000168100, 13187008, 14784000, 60199004, 77721001, 87132004, 191819002, 216463005, 216464004, 242828004, 242829007, 242831003, 290182008, 290183003, 295161000, 295163002, 295164008, 295165009, 295170002, 295171003, 295172005, 295173000, 295174006, 295175007, 295176008, 295184007, 295186009, 426001001, 242253008;<br>o **Cannabis:** 37344009, 39807006, 191838006, 191893000, 191894006, 428823006, 737336003, 26714005, 39951001, 63649001, 77355000, 85005007, 268641003, 703848005;<br>o **Stimulants:** 21647008, 27956007, 31956009, 78267003, 191831000, 191916008, 191918009, 191919001, 191920007, 296321004, 428493006, 428659002, 429782000, 441527004, 442406005, 699449003, 762504005, 762505006, 762671008, 785277001, 1155772001, 1230071005, 1230072003, 1230082002, 1230084001, 1230085000, 1230086004, 1230087008, 1230088003, 1230089006, 1231319004, 145101000119102, 429001000124103, 12398571000119100, 8837000, 45421006, 46975003, 51493001, 80868005, 84758004, 290544006, 290545007, 296322006, 296323001, 428370001<br>o **Sedatives or other:** : 7071007, 38247002, 50320000, 60901005, 64386003, 70340006, 74851005, 105549004, 191849000, 191856006, 191899001, 191900006, 231462006, 231468005, 268646008, 275471001, 426095000, 426590003, 427229002, 428495004, 428623008, 724713006, 724715004, 772999000, 1230074002, 1230077009, 1230081009, 1231160001, 86391000119101, 125851000119106, 145841000119107, 1382351000168100, 1382391000168100, 1382421000168100, 1382521000168100, 1382531000168100, 1382541000168100, 1382551000168100, 1382561000168100, 1382571000168100, 1382581000168100, 88320008, 191853003, 231473004;<br>o **Drug unspecified or multiple:** 2403008, 6525002, 9769006, 23915005, 26416006, 49540005, 56876005, 61480009, 66214007, 70545002, 87106005, 91388009, 95607001, 105546006, 110281001, 182969009, 191934007, 191936009, 191937000, 191938005, 226034001, 228371004, 228372006, 228373001, 228374007, 228375008, 228376009, 228377000, 228378005, 228379002, 228381000, 228382007, 228383002, 228384008, 228386005, 228387001, 228388006, 228389003, 228390007, 228391006, 228394003, 228401002, 228415000,

Details of data used	Codes/variables (ICD-10-AM and SNOMED-CT code definitions are provided in the separate Supplemental File 6a)
	228421001, 228422008, 228423003, 228425005, 228429004, 266707007, 307052004, 361049005, 361055000, 386450006, 386451005, 414054004, 414056002, 416262000, 416437003, 417252002, 417284009, 424848002, 425533007, 440664005, 442229000, 445273005, 708079007, 713775002, 741063003, 772808000, 1149322001, 1231333004, 1254812006, 1254974001, 1254987007, 1255017007, 1255020004, 1259017004, 1334001000168100, 1545151000168100, 1648321000168100, 1648331000168100, 16076691000119100, 11061003, 11196001, 11387009, 28368009, 32709003, 43242008, 50026000, 51339003, 74934004, 84584008, 191483003, 191484009, 191485005, 191486006, 191492000, 191494004, 191495003, 191496002, 191816009, 191865004, 191873008, 191939002, 228438002, 231451006, 231466009, 231481003, 231482005, 247702001, 290220008, 290221007, 290222000, 295154004, 295167001, 295169003, 295190006, 295193008, 295194002, 295213004, 297199006, 363101005, 363314000, 365984004, 396344000, 416119007, 429299000, 429672007, 442351006, 62213004
Receipt (commencement or continuation) of opioid agonist treatment in the mother's records.	**Mother records:**<br>**Any substance:** *Receipt (commencement or continuation) of opioid agonist treatment during or before the study period/s, individuals with no treatment end date were assumed to be in treatment.*<br>• ***Other drugs:*** Receipt (commencement or continuation) of opioid agonist treatment during or before the study period/s, in*dividuals with no treatment end date were assumed to be in treatment*.
Child protection records	
Primary and other issue/s reported to the child protection helpline<br>and<br>Primary and other assessed Issue/s at field assessment indicating use of substances in the children's records.	Any substance: Combined measure of alcohol and other drugs, and the below variables indicating substance use:<br>• *Reported issues group* and/or *Field assessment group*:<br>o Drug/alcohol use by carer<br>• *Primary helpline assessed issue, Other helpline assessed issues, Other field assessment assessed issue,* and *Primary field assessment assed issue:*<br>o drug/alcohol abuse by carer,<br>o Neglect: exposure to drug/alcohol use<br>o Parental risk factor: substance use<br>o Parents substance use led to emotional harm<br>• Alcohol: *Primary helpline assessed issue, Other helpline assessed issues, Other field assessment assessed issue,* and *Primary field assessment assed issue:*<br>o alcohol abuse by carer<br>• Other drugs: *Primary helpline assessed issue, Other helpline assessed issues, Other field assessment assessed issue,* and *Primary field assessment assed issue:*<br>o drug abuse by carer

1. Definitions for ICD-10 AM and SNOMED CT codes provided in Supplemental file 6a

Supplemental file 7. Sources used to inform the selection of ICD-10-AM and SNOMED codes

Published literature:

1. Burns L, Mattick RP, Cooke M. The use of record linkage to examine illicit drug use in pregnancy. 2006;101(6):873-82.
2. Yuen WS, Leung J, Man N, Chiu V, Gisev N, Livingston M, et al. Age at first alcohol-related hospital separation or emergency department presentation and rate of re-admission: A retrospective data linkage cohort of young Australians. Drug and alcohol review. 2022;41(7):1577-88.
3. Zhang J, Qian S, Su G, Deng C, Reid D, Curtis K, et al. Emergency department presentations of patients with alcohol use disorders in an Australian regional health district. Subst Abus. 2022;43(1):1126-38.
4. Whitlam G, Dinh M, Rodgers C, Muscatello DJ, McGuire R, Ryan T, et al. Diagnosis-based emergency department alcohol harm surveillance: What can it tell us about acute alcohol harms at the population level? Drug and alcohol review. 2016;35(6):693-701.
5. Oni HT, Buultjens M, Mohamed AL, Islam MM. Neonatal Outcomes of Infants Born to Pregnant Women With Substance Use Disorders: A Multilevel Analysis of Linked Data. Substance use & misuse. 2022;57(1):1-10.
6. Xu F, Bonello M, Burns L, Austin M-P, Li Z, Sullivan E. Hospital Admissions for Alcohol Use Disorders Before, During, and After Pregnancy: A Study Based on Linked Population Data in New South Wales, Australia. 2013;37(10):1706-12.
7. Kelty EA, Cumming CN, Preen DB. Contribution of pharmaceutical drugs of dependence to the incidence of neonatal abstinence syndrome in Western Australia between 2003 and 2018. Pharmacotherapy. 2022;42(5):405-10.
8. Burns L, Mattick RP, Cooke M. Use of record linkage to examine alcohol use in pregnancy. Alcoholism, clinical and experimental research. 2006;30(4):642-8.
9. Prindle JJ, Hammond I, Putnam-Hornstein E. Prenatal substance exposure diagnosed at birth and infant involvement with child protective services. Child abuse & neglect. 2018;76:75-83.
10. Burns L, Mattick RP. Using population data to examine the prevalence and correlates of neonatal abstinence syndrome. Drug and alcohol review. 2007;26(5):487-92.

Government reports:

1. NSW Ministry of Health. Alcohol Use and Related Harms in NSW: Surveillance Report 2021. Sydney: NSW Ministry of Health; 2022.
2. Chrzanowska A, Man, N, Akhurst, J, Sutherland, R, Degenhardt, L,, Peacock A. Trends in drug-related hospitalisations in Australia, 1999-2021. Sydney: National Drug and Alcohol Research Centre, UNSW Sydney; 2022.

Syndicated terminology server (Ontoserver) for SNOMED

1. Metke-Jimenez A, Steel J, Hansen D, Lawley M. Ontoserver: a syndicated terminology server. Journal of Biomedical Semantics. 2018;9(1):24.

Supplemental file 8. Outcome ascertainment periods

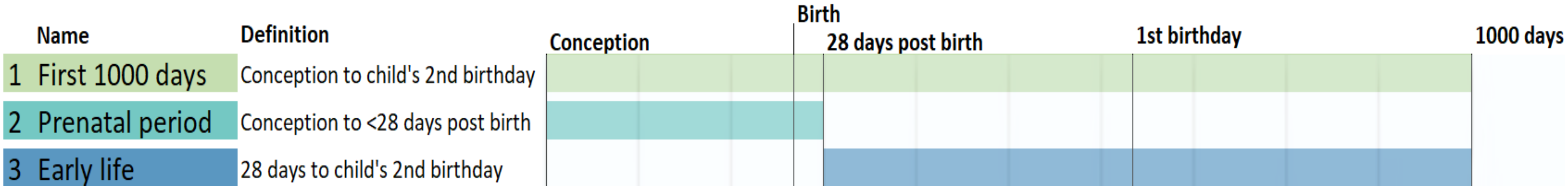

Supplemental file 9. Supporting information for Table 1. Maternal, pregnancy and child characteristics at birth of children with and without maternal substance use problems recorded during the first 1000 days

	Total		No Maternal Substance use recorded in any data		Maternal substance use recorded in ≥**1 data sources**					
					Child protection data[1]		Health +/or death data		Any data source	
	n	%	n	%	n	%	n	%	n	%
**Total children**	970,470		937,823		26,045		13,975		32,647	
Child's year of birth										
2008	96,288	9.9	91,731	9.8	3,936	15.1	1,616	11.6	4,557	14.0
2009	96,390	9.9	92,648	9.9	3,187	12.2	1,356	9.7	3,742	11.5
2010	96,427	9.9	93,401	10.0	2,337	9.0	1,414	10.1	3,026	9.3
2011	97,187	10	94,250	10.0	2,221	8.5	1,381	9.9	2,937	9.0
2012	99,458	10.2	96,398	10.3	2,357	9.0	1,430	10.2	3,060	9.4
2013	96,899	10.0	93,707	10.0	2,549	9.8	1,371	9.8	3,192	9.8
2014	97,279	10.0	94,026	10.0	2,566	9.9	1,419	10.2	3,253	10
2015	96,345	9.9	93,220	9.9	2,448	9.4	1,407	10.1	3,125	9.6
2016	98,472	10.1	95,559	10.2	2,256	8.7	1,321	9.5	2,913	8.9
2017	95,725	9.9	92,883	9.9	2,188	8.4	1,260	9.0	2,842	8.7
**Total, denominator for column**	**970,470**	**100**	**937,823**	**100**	**26,045**	**100**	**13,975**	**100**	**32,647**	**100**
Child's sex										
Male	498,280	51.3	<482,000	<52.0	13,258	50.9	<7,300	<52.0	<16,700	<52.0
Female	472,128	48.6	456,167	48.6	12,787	49.1	6,772	48.5	15,961	48.9
Intersex, indeterminate	46	<0.1	<46	<0.1	0	0	<46	<0.1	<46	<0.1
Missing	16	<0.1	16	<0.1	0	0	0	0	0	0
**Total, denominator for column**	**970,470**	**100**	**937,823**	**100**	**26,045**	**100**	**13,975**	**100**	**32,647**	**100**
Aboriginal and/or Torres Strait Islander child[2]										
No	903,266	93.1	882,875	94.1	15,690	60.2	9,016	64.5	20,391	62.5
Yes	67,186	6.9	54,930	5.9	10,355	39.8	4,959	35.5	12,256	37.5
Missing	18	<0.1	18	<0.1	0	0	0	0	0	0
**Total, denominator for column**	**970,470**	**100**	**937,823**	**100**	**26,045**	**100**	**13,975**	**100**	**32,647**	**100**
Preterm (<37 weeks)										
No	897,569	92.5	870,033	92.8	22,136	85	11,222	80.3	27,536	84.3
Yes	72,901	7.5	67,790	7.2	3,909	15	2,753	19.7	5,111	15.7
**Total, denominator for column**	**970,470**	**100**	**937,823**	**100**	**26,045**	**100**	**13,975**	**100**	**32,647**	**100**
Small for gestational age										
**No**	873148	90.0	846820	90.3	21058	80.9	10866	77.8	26328	80.6
**Yes**	96420	9.9	90125	9.6	4974	19.1	3091	22.1	6295	19.3
**Missing**	902	0.1	878	0.1	13	0.0	18	0.1	24	0.1
**Total, denominator for column**	**970470**	**100**	**937823**	**100**	**26045**	**100**	**13975**	**100**	**32647**	**100**
Low birth weight <2500g										
No	908,670	93.6	881,340	94	22,008	84.5	11,012	78.8	27,330	83.7
Yes	61,030	6.3	55,734	5.9	4,027	15.5	2,947	21.1	5,296	16.2
Missing	770	0.1	749	0.1	10	<0.1	16	0.1	21	0.1
**Total, denominator for column**	**970,470**	**100**	**937,823**	**100**	**26,045**	**100**	**13,975**	**100**	**32,647**	**100**
Admitted to Neonatal Intensive care unit or Special care nursery (Data available for children born 2008-2015)[3]										
Not recorded in data	9	<0.1	9	<0.1	0	<0.1	0	<0.1	0	<0.1
No admission	662,543	85.3	644,038	85.9	15,016	69.5	6,557	57.5	18,505	68.8
Admitted to NICU or SCN	113,167	14.6	104,803	14.0	6,566	30.4	4,826	42.4	8,364	31.1
Missing	554	0.1	531	0.1	19	0.1	11	0.1	23	0.1
**Total, denominator for column (children born 2008-2015)**	**776,273**	**100**	**749,381**	**100**	**21,601**	**100**	**11,394**	**100**	**26,892**	**100**
Mothers age at delivery (years)										
Missing	13	<0.1	13	0	0	0	0	0	0	0
<20	27,512	2.8	22,726	2.4	4,150	15.9	1,382	9.9	4,786	14.7
24	120,840	12.5	112,028	11.9	7,136	27.4	3,401	24.3	8,812	27

	Total		No Maternal Substance use recorded in any data		Maternal substance use recorded in **≥1 data sources**					
					Child protection data[1]		Health +/or death data		Any data source	
	n	%	n	%	n	%	n	%	n	%
29	260,485	26.8	252,548	26.9	6,255	24	3,672	26.3	7,937	24.3
34	328,250	33.8	321,960	34.3	4,833	18.6	3,121	22.3	6,290	19.3
35 +	233,370	24	228,548	24.4	3,671	14.1	2,399	17.2	4,822	14.8
Total, denominator for column	970,470	100	937,823	100	26,045	100	13,975	100	32,647	100
Aboriginal or Torres Strait Islander mother										
Not recorded in data	21,722	2.2	19,028	2	1,934	7.4	1,228	8.8	2,694	8.3
Aboriginal or Torres Strait Islander	51,003	5.3	41,914	4.5	7,796	29.9	3,674	26.3	9,089	27.8
Non-Aboriginal	897,616	92.5	876,764	93.5	16,304	62.6	9,066	64.9	20,852	63.9
Missing	129	<0.1	117	<0.1	11	<0.1	7	0.1	12	<0.1
Total, denominator for column	970,470	100	937,823	100	26,045	100	13,975	100	32,647	100
Mother's country of birth										
Australia	634,493	65.4	604,849	64.5	23,798	91.4	12,671	90.7	29,644	90.8
New Zealand/Pacific	34,884	3.6	33,727	3.6	911	3.5	460	3.3	1,157	3.5
Europe	51,520	5.3	51,020	5.4	359	1.4	243	1.7	500	1.5
Africa and the Middle East	55,734	5.7	55,366	5.9	255	1.0	164	1.2	368	1.1
South-East Asia	54,175	5.6	53,680	5.7	377	1.4	227	1.6	495	1.5
North-East Asia	56,943	5.9	56,783	6.1	114	0.4	64	0.5	160	0.5
Southern and Central Asia	62,139	6.4	62,003	6.6	92	0.4	51	0.4	136	0.4
Americas	17,387	1.8	17,224	1.8	119	0.5	80	0.6	163	0.5
Missing	3,195	0.3	3,171	0.3	20	0.1	15	0.1	24	0.1
Total, denominator for column	970,470	100	937,823	100	26,045	100	13,975	100	32,647	100
Married or in de facto partnership[4]										
No	137,720	14.2	118,820	12.7	15,560	59.7	8,087	57.9	18,900	57.9
Yes	808,753	83.3	795,798	84.9	9,845	37.8	5,538	39.6	12,955	39.7
Missing	23,997	2.5	23,205	2.5	640	2.5	350	2.5	792	2.4
Total, denominator for column	970,470	100	937,823	100	26,045	100	13,975	100	32,647	100
Private health insurance/patient[5]										
Not recorded in data	58	0	58	0	0	0	0	0	0	0
No	764,546	78.8	732,503	78.1	25,745	98.8	13,632	97.5	32,043	98.1
Yes	205,866	21.2	205,262	21.9	300	1.2	343	2.5	604	1.9
Total, denominator for column	970,470	100	937,823	100	26,045	100	13,975	100	32,647	100
Mothers area of residence[6] (missing vales n=<5)										
Not recorded in data	16,713	1.7	16,312	1.7	293	1.1	227	1.6	401	1.2
Lived in major city	751,815	77.5	732,726	78.1	15,059	57.8	8,719	62.4	19,089	58.5
Lived in inner regional area	150,122	15.5	140,645	15.0	7,678	29.5	3,698	26.5	9,477	29.0
Lived in outer regional area	47,059	4.8	43,823	4.7	2,630	10.1	1,188	8.5	3,236	9.9
Lived in remote/very remote area	4,759	0.4	4,317	0.4	385	1.9	143	1.5	444	1.8
Total, denominator for column	970,470	100	937,823	100	26,045	100	13,975	100	32,647	100
Area-level disadvantage[7]										
Not recorded in data	370	0	357	0	7	0	11	0.1	13	0
Quintile 1 (most disadvantaged)	213,028	22	200,803	21.4	9,893	38	5,102	36.5	12,225	37.4
Quintile 2	177,648	18.3	169,072	18	6,982	26.8	3,417	24.5	8,576	26.3
Quintile 3	211,875	21.8	205,204	21.9	5,383	20.7	2,785	19.9	6,671	20.4
Quintile 4	148,809	15.3	145,901	15.6	2,235	8.6	1,415	10.1	2,908	8.9
Quintile 5 (least disadvantaged)	218,740	22.5	216,486	23.1	1,545	5.9	1,245	8.9	2,254	6.9
Total, denominator for column	970,470	100	937,823	100	26,045	100	13,975	100	32,647	100
Living in or awaiting public housing two years prior to child's birth[8]										
No	925,155	95.3	903,672	96.4	16,514	63.4	9,165	65.6	21,483	65.8
Yes	45,315	4.7	34,151	3.6	9,531	36.6	4,810	34.4	11,164	34.2
Total, denominator for column	970,470	100	937,823	100	26,045	100	13,975	100	32,647	100
Number of previous pregnancies										
Mother had no prior births	420,220	43.3	409,737	43.7	7,975	30.6	4,511	32.3	10,483	32.1

	Total		No Maternal Substance use recorded in any data		Maternal substance use recorded in **≥1 data sources**					
					Child protection data[1]		Health +/or death data		Any data source	
	n	%	n	%	n	%	n	%	n	%
Mother had one prior birth	326,902	33.7	318,947	34	6,201	23.8	3,434	24.6	7,955	24.4
Mother had two or more prior births	222,591	22.9	208,421	22.2	11,842	45.5	6,005	43	14,170	43.4
Missing	757	0.1	718	0.1	27	0.1	25	0.2	39	0.1
**Total, denominator for column**	**970,470**	**100**	**937,823**	**100**	**26,045**	**100**	**13,975**	**100**	**32,647**	**100**
Received antenatal care in first 20 weeks of pregnancy										
No	102125	10.5	94581	10.1	6256	24.0	3365	24.1	7544	23.1
Yes	856670	88.3	832730	88.8	18778	72.1	9993	71.5	23940	73.3
Missing	11675	1.2	10512	1.1	1011	3.9	617	4.4	1163	3.6
**Total**	**970470**	**100**	**937823**	**100**	**26045**	**100**	**13975**	**100**	**32647**	**100**
Mother smoked during pregnancy[9]										
No	870,883	89.7	858,927	91.6	9,025	34.7	4,326	31	11,956	36.6
Yes	99,260	10.2	78,612	8.4	16,985	65.2	9,630	68.9	20,648	63.2
Missing	327	<0.1	284	<0.1	35	0.1	19	0.1	43	0.1
**Total, denominator for column**	**970,470**	**100**	**937,823**	**100**	**26,045**	**100**	**13,975**	**100**	**32,647**	**100**

1.The child protection data source includes maternal and other carer substance use; 2. Defined as child or parent identified as Aboriginal on any of the birth records (i.e. perinatal data collection, birth registration or hospital record; 3. Numbers and percents calculated for children born from 2008-2015 (N=776,273) when this variable was collected in the Perinatal data collection; 4. The month and year of birth for the mother was obtained from the birth registration, or the Perinatal Data Collection if the birth registration was unavailable, and used to calculate maternal age at childbirth, in years; 5. As recorded on the hospital birth record; 6. Based on the Accessibility/Remoteness Index of Australia (ARIA+) for the Statistical Local Area of residence at the child’s birth; 7. Based on the Index of Relative Socio-economic Advantage and Disadvantage for the Statistical Local Area of residence at the child’s birth; 8. Mother is living in public housing or is on the tenancy waitlist at any time 2 years prior to the child’s birth. 9. Variable derived from variables recorded in Perinatal Data Collection, indicates if the mother smoked at any time point during pregnancy.

Supplemental file 10. Number, percentage, and 95% CIS of children with maternal substance use problems in the first 1000 days, ascertained from six data sources, for children born in NSW from 2008-2017

N = 970, 470	Single data sources												Combined data sources			
Data source	Cause of death[1]		Mental health outpatients		Opioid treatment register		Emergency department		Hospital		Child protection[2]		Health +/or cause of death[3]		Any data source[3]	
Substance type	n	% (95% CI)	n	% (95% CI)	n	% (95% CI)	n	% (95% CI)	n	% (95% CI)	n	% (95% CI)	n	% (95% CI)	n	% (95% CI)
**Maternal data sources (5 data sources)**																
Any substance[4]	<64	0.01 (<0.01-0.01)	964	0.10 (0.09-0.11)	3,229	0.33 (0.32-0.34)	2,203	0.23 (0.22-0.24)	10,793	1.11 (1.09-1.13)	-	-	-	-	12,956	1.34 (1.31-1.36)
Alcohol	-	-	511	<0.01	-	-	1,431	0.15 (0.14-0.16)	3,046	0.31 (0.3-0.32)	-	-	-	-	4,246	0.44 (0.42-0.45)
Other drug[5]	-	-	473	<0.01	3,229	0.33 (0.32-0.34)	814	0.08 (0.08-0.09)	8,985	0.93 (0.91-0.94)	-	-	-	-	10,126	1.04 (1.02-1.06)
Opioid/opioid treatment	-	-	30	<0.01	3,229	0.33 (0.32-0.34)	140	0.01 (0.01-0.02)	3,063	0.32 (0.3-0.33)	-	-	-	-	4,011	0.41 (0.4-0.43)
Cannabis	-	-	107	<0.01	-	-	43	0 (0-0.01)	4,435	0.46 (0.44-0.47)	-	-	-	-	4,505	0.46 (0.45-0.48)
Stimulant	-	-	102	<0.01	-	-	56	0.01 (0-0.01)	2,117	0.22 (0.21-0.23)	-	-	-	-	2,182	0.22 (0.22-0.23)
Sedatives or other[6]	-	-	6	<0.01	-	-	10	0 (0-0)	549	0.06 (0.05-0.06)	-	-	-	-	572	0.06 (0.05-0.06)
Unspecified or multiple[7]	-	-	342	<0.01	-	-	618	0.06 (0.06-0.07)	1,858	0.19 (0.18-0.2)	-	-	-	-	2,576	0.27 (0.26-0.28)
**Child data sources (3 data sources)**																
Any substance[4]	<5	<0.01	-	-	-	-	-	-	3,827	0.39 (0.38-0.41)	26,045	2.68 (2.65-2.72)	-	-	27,327	2.82 (2.78-2.85)
Alcohol	-	-	-	-	-	-	-	-	62	0.01 (<0.01-0.01)	10,702	1.1 (1.08-1.12)	-	-	10,726	1.11 (1.08-1.13)
Other drug[5]	-	-	-	-	-	-	-	-	3,787	0.39 (0.38-0.40)	18,152	1.87 (1.84-1.9)	-	-	19,559	2.02 (1.99-2.04)
**Child and maternal data sources combined (6 data sources)**																
Any substance[4]	<64	0.01 (<0.01-0.01)	964	0.10 (0.09-0.11)	3,229	0.33 (0.32-0.34)	2,203	0.23 (0.22-0.24)	12,067	1.24 (1.22-1.27)	26,045	2.68 (2.65-2.72)	13,975	1.44 (1.42-1.46)	32,647	3.36 (3.33-3.40)
Alcohol	-	-	511	<0.01	-	-	1,431	0.15 (0.14-0.16)	3,074	0.32 (0.31-0.33)	10,702	1.1 (1.08-1.12)	11,175	1.15 (1.13-1.17)	13,637	1.41 (1.38-1.43)
Other drug[5]	-	-	473	<0.01	3,229	0.33 (0.32-0.34)	814	0.08 (0.08-0.09)	10,282	1.06 (1.04-1.08)	18,152	1.87 (1.84-1.9)	4,272	0.44 (0.43-0.45)	23,485	2.42 (2.39-2.45)

1.For cause of death data, reporting is limited to the outcome “any substance use” due to low numbers in specific substance types; 2. The child protection data source includes maternal and other carer substance use; 3. The outcomes are not mutually exclusive across data sources or substance types; therefore, numbers in rows/columns do not sum to numbers reported in “All records and data sources” (rows) or the “Any substance use”outcome (columns); 4. Any substance use includes the use of alcohol and or other drug use; 5. Other drug use includes the use of drugs other than alcohol and tobacco, including illicit drugs, misuse of prescription drugs, and use of opioid agonist treatment; 6. “Sedatives or other” includes the use of sedatives, hallucinogens, sedatives, solvents, or organic compounds, which were aggregated due to small cell sizes in individual substance categories; 7. “Unspecified or multiple substance use” includes ICD10AM codes that indicate multiple drug use or do not specify the type of drug or psychoactive substance.

Supplemental file 11. Data for Figure 1. Number of children with maternal substance use problems in different combinations of data sources

Child protection	Child hospital	Child cause of death	Mother hospital	Mother cause of death	Mother mental health outpatient	Mother opioid treatment register	Mother emergency department	Number of data sources	n	N	%	SE	Lower 95% CI	Upper 95% CI
No	No	No	No	No	No	No	No	0	937,823	970,470	0.9664	0.0002	0.9660	0.9667
Yes	No	No	No	No	No	No	No	1	18,672	970,470	0.0192	0.0001	0.0190	0.0195
No	No	No	Yes	No	No	No	No	1	3,283	970,470	0.0034	0.0001	0.0033	0.0035
Yes	No	No	Yes	No	No	No	No	2	3,057	970,470	0.0032	0.0001	0.0030	0.0033
Yes	Yes	No	Yes	No	No	Yes	No	4	1,052	970,470	0.0011	0.0000	0.0010	0.0011
No	No	No	No	No	No	No	Yes	1	809	970,470	0.0008	0.0000	0.0008	0.0009
Yes	Yes	No	Yes	No	No	No	No	3	549	970,470	0.0006	0.0000	0.0005	0.0006
No	Yes	No	No	No	No	No	No	1	544	970,470	0.0006	0.0000	0.0005	0.0006
Yes	Yes	No	No	No	No	No	No	2	473	970,470	0.0005	0.0000	0.0004	0.0005
Yes	No	No	Yes	No	No	Yes	No	3	392	970,470	0.0004	0.0000	0.0004	0.0004
No	Yes	No	Yes	No	No	Yes	No	3	385	970,470	0.0004	0.0000	0.0004	0.0004
Yes	No	No	Yes	No	No	No	Yes	3	375	970,470	0.0004	0.0000	0.0003	0.0004
No	No	No	Yes	No	No	Yes	No	2	306	970,470	0.0003	0.0000	0.0003	0.0004
No	No	No	No	No	No	Yes	No	1	275	970,470	0.0003	0.0000	0.0002	0.0003
No	No	No	Yes	No	No	No	Yes	2	274	970,470	0.0003	0.0000	0.0002	0.0003
Yes	No	No	No	No	No	No	Yes	2	230	970,470	0.0002	0.0000	0.0002	0.0003
Yes	No	No	Yes	No	Yes	No	No	3	221	970,470	0.0002	0.0000	0.0002	0.0003
No	Yes	No	Yes	No	No	No	No	2	211	970,470	0.0002	0.0000	0.0002	0.0002
Yes	No	No	No	No	No	Yes	No	2	189	970,470	0.0002	0.0000	0.0002	0.0002
No	No	No	No	No	Yes	No	No	1	186	970,470	0.0002	0.0000	0.0002	0.0002
Yes	Yes	No	No	No	No	Yes	No	3	141	970,470	0.0001	0.0000	0.0001	0.0002
Yes	No	No	No	No	Yes	No	No	2	133	970,470	0.0001	0.0000	0.0001	0.0002
Yes	Yes	No	Yes	No	No	Yes	Yes	5	116	970,470	0.0001	0.0000	0.0001	0.0001
Yes	No	No	Yes	No	Yes	No	Yes	4	90	970,470	<0.0001	<0.0001	<0.0001	<0.0001
No	Yes	No	No	No	No	Yes	No	2	87	970,470	<0.0001	<0.0001	<0.0001	<0.0001
No	No	No	Yes	No	Yes	No	No	2	84	970,470	<0.0001	<0.0001	<0.0001	<0.0001
Yes	Yes	No	Yes	No	Yes	Yes	No	5	56	970,470	<0.0001	<0.0001	<0.0001	<0.0001
Yes	Yes	No	Yes	No	No	No	Yes	4	51	970,470	<0.0001	<0.0001	<0.0001	<0.0001
Yes	No	No	Yes	No	No	Yes	Yes	4	50	970,470	<0.0001	<0.0001	<0.0001	<0.0001
Yes	Yes	No	Yes	No	Yes	No	No	4	32	970,470	<0.0001	<0.0001	<0.0001	<0.0001
No	No	No	Yes	No	No	Yes	Yes	3	30	970,470	<0.0001	<0.0001	<0.0001	<0.0001
Yes	No	No	Yes	No	Yes	Yes	No	4	23	970,470	<0.0001	<0.0001	<0.0001	<0.0001
No	No	No	Yes	No	Yes	No	Yes	3	22	970,470	<0.0001	<0.0001	<0.0001	<0.0001
No	Yes	No	Yes	No	No	Yes	Yes	4	21	970,470	<0.0001	<0.0001	<0.0001	<0.0001
Yes	Yes	No	Yes	No	Yes	No	Yes	5	19	970,470	<0.0001	<0.0001	<0.0001	<0.0001

Child protection	Child hospital	Child cause of death	Mother hospital	Mother cause of death	Mother mental health outpatient	Mother opioid treatment register	Mother emergency department	Number of data sources	n	N	%	SE	Lower 95% CI	Upper 95% CI
Yes	Yes	No	Yes	No	Yes	Yes	Yes	6	19	970,470	<0.0001	<0.0001	<0.0001	<0.0001
Yes	No	No	No	Yes	No	No	No	2	17	970,470	<0.0001	<0.0001	<0.0001	<0.0001
No	No	No	No	Yes	No	No	No	1	15	970,470	<0.0001	<0.0001	<0.0001	<0.0001
Yes	No	No	No	No	Yes	No	Yes	3	15	970,470	<0.0001	<0.0001	<0.0001	<0.0001
Yes	No	No	No	No	No	Yes	Yes	3	14	970,470	<0.0001	<0.0001	<0.0001	<0.0001
No	No	No	No	No	No	Yes	Yes	2	11	970,470	<0.0001	<0.0001	<0.0001	<0.0001
No	No	No	No	No	Yes	No	Yes	2	10	970,470	<0.0001	<0.0001	<0.0001	<0.0001
Yes	No	No	Yes	No	Yes	Yes	Yes	5	10	970,470	<0.0001	<0.0001	<0.0001	<0.0001
No	Yes	No	Yes	No	No	No	Yes	3	9	970,470	<0.0001	<0.0001	<0.0001	<0.0001
Yes	Yes	No	Yes	Yes	No	Yes	No	5	9	970,470	<0.0001	<0.0001	<0.0001	<0.0001
No	No	No	Yes	No	Yes	Yes	No	3	8	970,470	<0.0001	<0.0001	<0.0001	<0.0001
No	Yes	No	Yes	No	Yes	No	No	3	7	970,470	<0.0001	<0.0001	<0.0001	<0.0001
No	Yes	No	Yes	No	Yes	Yes	Yes	5	6	970,470	<0.0001	<0.0001	<0.0001	<0.0001
Yes	Yes	No	No	No	No	No	Yes	3	6	970,470	<0.0001	<0.0001	<0.0001	<0.0001
Yes	Yes	No	No	No	Yes	No	No	3	6	970,470	<0.0001	<0.0001	<0.0001	<0.0001
Yes	No	No	Yes	Yes	No	No	No	3	5	970,470	<0.0001	<0.0001	<0.0001	<0.0001
Yes	Yes	No	No	No	No	Yes	Yes	4	5	970,470	<0.0001	<0.0001	<0.0001	<0.0001
Yes	No	No	Yes	Yes	No	Yes	No	4	<5	970,470	<0.0001	<0.0001	<0.0001	<0.0001
No	No	No	Yes	No	Yes	Yes	Yes	4	<5	970,470	<0.0001	<0.0001	<0.0001	<0.0001
No	Yes	No	No	No	No	No	Yes	2	<5	970,470	<0.0001	<0.0001	<0.0001	<0.0001
No	Yes	No	No	No	Yes	No	No	2	<5	970,470	<0.0001	<0.0001	<0.0001	<0.0001
No	Yes	No	Yes	No	Yes	Yes	No	4	<5	970,470	<0.0001	<0.0001	<0.0001	<0.0001
No	No	No	Yes	Yes	No	Yes	No	3	<5	970,470	<0.0001	<0.0001	<0.0001	<0.0001
Yes	Yes	No	No	No	Yes	Yes	No	4	<5	970,470	<0.0001	<0.0001	<0.0001	<0.0001
Yes	Yes	No	Yes	Yes	No	Yes	Yes	6	<5	970,470	<0.0001	<0.0001	<0.0001	<0.0001
Yes	Yes	Yes	Yes	No	No	Yes	No	5	<5	970,470	<0.0001	<0.0001	<0.0001	<0.0001
No	No	No	No	No	Yes	Yes	No	2	<5	970,470	<0.0001	<0.0001	<0.0001	<0.0001
No	No	No	No	Yes	No	Yes	No	2	<5	970,470	<0.0001	<0.0001	<0.0001	<0.0001
No	Yes	No	No	No	Yes	Yes	No	3	<5	970,470	<0.0001	<0.0001	<0.0001	<0.0001
No	Yes	No	Yes	No	Yes	No	Yes	4	<5	970,470	<0.0001	<0.0001	<0.0001	<0.0001
No	Yes	No	Yes	Yes	No	Yes	No	4	<5	970,470	<0.0001	<0.0001	<0.0001	<0.0001
Yes	No	No	No	No	Yes	Yes	No	3	<5	970,470	<0.0001	<0.0001	<0.0001	<0.0001
Yes	No	No	Yes	Yes	Yes	No	No	4	<5	970,470	<0.0001	<0.0001	<0.0001	<0.0001
Yes	No	Yes	No	No	No	No	No	2	<5	970,470	<0.0001	<0.0001	<0.0001	<0.0001
Yes	Yes	No	No	No	Yes	Yes	Yes	5	<5	970,470	<0.0001	<0.0001	<0.0001	<0.0001
Yes	Yes	No	No	Yes	No	No	No	3	<5	970,470	<0.0001	<0.0001	<0.0001	<0.0001

Child protection	Child hospital	Child cause of death	Mother hospital	Mother cause of death	Mother mental health outpatient	Mother opioid treatment register	Mother emergency department	Number of data sources	n	N	%	SE	Lower 95% CI	Upper 95% CI
Yes	Yes	No	Yes	Yes	No	No	No	4	<5	970,470	<0.0001	<0.0001	<0.0001	<0.0001
Yes	Yes	No	Yes	Yes	No	No	Yes	5	<5	970,470	<0.0001	<0.0001	<0.0001	<0.0001
Yes	Yes	Yes	No	No	No	No	No	3	<5	970,470	<0.0001	<0.0001	<0.0001	<0.0001

Footnotes: Total number of children with values <5 is 37; 1. n = numerator, number of children with maternal substance use problems recorded in the combination of data sources; N = denominator, total children born from 2008-2017; SE: Standard error; 95% CI: 95% confidence intervals

Supplemental file 12. Most common combinations of substance types recorded in maternal data sources, among children born in NSW from 2008-2017 (n=970,470)

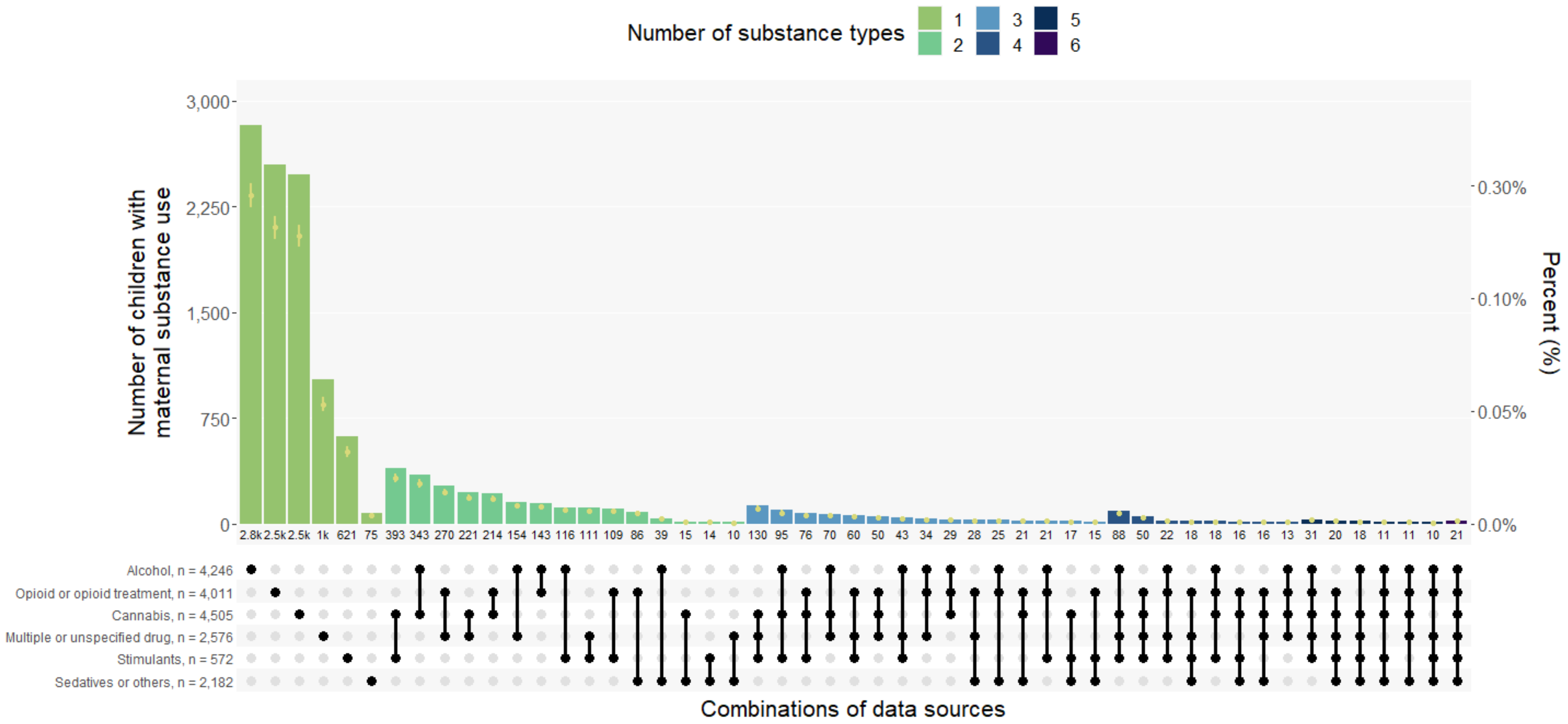

Footnotes: 86 children were not included in upset plot as we excluded combinations of substances with less than 10 children. All values for numerators, denominators, and 95% confidence intervals provided in Supplemental file 13. Definition: “Sedatives or other” includes the use of sedatives, hallucinogens, sedatives, solvents, or organic compounds, which were aggregated due to small cell sizes in individual substance categories; “Unspecified or multiple substance use” includes ICD-10-AM codes that indicate multiple drug use or do not specify the type of drug or psychoactive substance.

Supplemental file 13. Data to accompany Supplemental file 12. Number of children with maternal substance use problems in different combinations of substance types recorded in maternal records

Opioid	Alcohol	Cannabis	Sedatives or other[1]	Unspecified or multiple[2]	Stimulant	Number of substance types	N[3]	N	%	SE	Lower 95% CI	Upper 95% CI
No	No	No	No	No	No	0	957495	970470	0.9866	0.0001	0.9864	0.9869
No	Yes	No	No	No	No	1	2825	970470	0.0029	0.0001	0.0028	0.0030
Yes	No	No	No	No	No	1	2550	970470	0.0026	0.0001	0.0025	0.0027
No	No	Yes	No	No	No	1	2477	970470	0.0026	0.0001	0.0025	0.0027
No	No	No	No	Yes	No	1	1026	970470	0.0011	0.0000	0.0010	0.0011
No	No	No	No	No	Yes	1	621	970470	0.0006	0.0000	0.0006	0.0007
No	No	Yes	No	No	Yes	2	393	970470	0.0004	0.0000	0.0004	0.0004
No	Yes	Yes	No	No	No	2	343	970470	0.0004	0.0000	0.0003	0.0004
Yes	No	No	No	Yes	No	2	270	970470	0.0003	0.0000	0.0002	0.0003
No	No	Yes	No	Yes	No	2	221	970470	0.0002	0.0000	0.0002	0.0003
Yes	No	Yes	No	No	No	2	214	970470	0.0002	0.0000	0.0002	0.0003
No	Yes	No	No	Yes	No	2	154	970470	0.0002	0.0000	0.0001	0.0002
Yes	Yes	No	No	No	No	2	143	970470	0.0001	0.0000	0.0001	0.0002
No	No	Yes	No	Yes	Yes	3	130	970470	0.0001	0.0000	0.0001	0.0002
No	Yes	No	No	No	Yes	2	116	970470	0.0001	0.0000	0.0001	0.0001
No	No	No	No	Yes	Yes	2	111	970470	0.0001	0.0000	0.0001	0.0001
Yes	No	No	No	No	Yes	2	109	970470	0.0001	0.0000	0.0001	0.0001
No	Yes	Yes	No	No	Yes	3	95	970470	<0.0001	<0.0001	<0.0001	<0.0001
No	Yes	Yes	No	Yes	Yes	4	88	970470	<0.0001	<0.0001	<0.0001	<0.0001
Yes	No	No	Yes	No	No	2	86	970470	<0.0001	<0.0001	<0.0001	<0.0001
Yes	No	Yes	No	No	Yes	3	76	970470	<0.0001	<0.0001	<0.0001	<0.0001
No	No	No	Yes	No	No	1	75	970470	<0.0001	<0.0001	<0.0001	<0.0001
No	Yes	Yes	No	Yes	No	3	70	970470	<0.0001	<0.0001	<0.0001	<0.0001
Yes	No	No	No	Yes	Yes	3	60	970470	<0.0001	<0.0001	<0.0001	<0.0001
Yes	No	Yes	No	Yes	No	3	50	970470	<0.0001	<0.0001	<0.0001	<0.0001
Yes	No	Yes	No	Yes	Yes	4	50	970470	<0.0001	<0.0001	<0.0001	<0.0001
No	Yes	No	No	Yes	Yes	3	43	970470	<0.0001	<0.0001	<0.0001	<0.0001
No	Yes	No	Yes	No	No	2	39	970470	<0.0001	<0.0001	<0.0001	<0.0001
Yes	Yes	No	No	Yes	No	3	34	970470	<0.0001	<0.0001	<0.0001	<0.0001
Yes	Yes	Yes	No	Yes	Yes	5	31	970470	<0.0001	<0.0001	<0.0001	<0.0001
Yes	Yes	Yes	No	No	No	3	29	970470	<0.0001	<0.0001	<0.0001	<0.0001
Yes	No	No	Yes	Yes	No	3	28	970470	<0.0001	<0.0001	<0.0001	<0.0001
Yes	Yes	No	Yes	No	No	3	25	970470	<0.0001	<0.0001	<0.0001	<0.0001

Opioid	Alcohol	Cannabis	Sedatives or other[1]	Unspecified or multiple[2]	Stimulant	Number of substance types	N[3]	N	%	SE	Lower 95% CI	Upper 95% CI
Yes	Yes	No	No	Yes	Yes	4	22	970470	<0.0001	<0.0001	<0.0001	<0.0001
Yes	No	Yes	Yes	No	No	3	21	970470	<0.0001	<0.0001	<0.0001	<0.0001
Yes	Yes	No	No	No	Yes	3	21	970470	<0.0001	<0.0001	<0.0001	<0.0001
Yes	Yes	Yes	Yes	Yes	Yes	6	21	970470	<0.0001	<0.0001	<0.0001	<0.0001
Yes	No	Yes	Yes	Yes	Yes	5	20	970470	<0.0001	<0.0001	<0.0001	<0.0001
No	Yes	Yes	Yes	Yes	Yes	5	18	970470	<0.0001	<0.0001	<0.0001	<0.0001
Yes	No	No	Yes	Yes	Yes	4	18	970470	<0.0001	<0.0001	<0.0001	<0.0001
Yes	Yes	Yes	No	No	Yes	4	18	970470	<0.0001	<0.0001	<0.0001	<0.0001
No	No	Yes	Yes	No	Yes	3	17	970470	<0.0001	<0.0001	<0.0001	<0.0001
Yes	No	Yes	Yes	No	Yes	4	16	970470	<0.0001	<0.0001	<0.0001	<0.0001
Yes	No	Yes	Yes	Yes	No	4	16	970470	<0.0001	<0.0001	<0.0001	<0.0001
No	No	Yes	Yes	No	No	2	15	970470	<0.0001	<0.0001	<0.0001	<0.0001
Yes	No	No	Yes	No	Yes	3	15	970470	<0.0001	<0.0001	<0.0001	<0.0001
No	No	No	Yes	No	Yes	2	14	970470	<0.0001	<0.0001	<0.0001	<0.0001
Yes	Yes	Yes	No	Yes	No	4	13	970470	<0.0001	<0.0001	<0.0001	<0.0001
Yes	Yes	Yes	Yes	No	Yes	5	11	970470	<0.0001	<0.0001	<0.0001	<0.0001
Yes	Yes	Yes	Yes	Yes	No	5	11	970470	<0.0001	<0.0001	<0.0001	<0.0001
No	No	No	Yes	Yes	No	2	10	970470	<0.0001	<0.0001	<0.0001	<0.0001
Yes	Yes	No	Yes	Yes	Yes	5	10	970470	<0.0001	<0.0001	<0.0001	<0.0001
No	No	No	Yes	Yes	Yes	3	9	970470	<0.0001	<0.0001	<0.0001	<0.0001
No	Yes	Yes	Yes	No	No	3	9	970470	<0.0001	<0.0001	<0.0001	<0.0001
No	Yes	Yes	Yes	Yes	No	4	9	970470	<0.0001	<0.0001	<0.0001	<0.0001
Yes	Yes	No	Yes	Yes	No	4	9	970470	<0.0001	<0.0001	<0.0001	<0.0001
No	Yes	No	Yes	No	Yes	3	8	970470	<0.0001	<0.0001	<0.0001	<0.0001
Yes	Yes	Yes	Yes	No	No	4	8	970470	<0.0001	<0.0001	<0.0001	<0.0001
No	Yes	No	Yes	Yes	No	3	7	970470	<0.0001	<0.0001	<0.0001	<0.0001
No	No	Yes	Yes	Yes	No	3	6	970470	<0.0001	<0.0001	<0.0001	<0.0001
No	Yes	No	Yes	Yes	Yes	4	6	970470	<0.0001	<0.0001	<0.0001	<0.0001
Yes	Yes	No	Yes	No	Yes	4	6	970470	<0.0001	<0.0001	<0.0001	<0.0001
No	No	Yes	Yes	Yes	Yes	4	≤5	970470	<0.0001	<0.0001	<0.0001	<0.0001
No	Yes	Yes	Yes	No	Yes	4	≤5	970470	<0.0001	<0.0001	<0.0001	<0.0001

Footnotes: 1. “Sedatives or other” includes the use of sedatives, hallucinogens, sedatives, solvents, or organic compounds, which were aggregated due to small cell sizes in individual substance categories;2. “Unspecified or multiple substance use” includes ICD-10-AM codes that indicate multiple drug use or do not specify the type of drug or psychoactive substance. 3. Total number of children with values ≤5 is 9. Definitions: n = numerator, number of children with maternal substance use problems recorded in the combination of data sources; N = denominator, total children born from 2008-2017; SE: Standard error; 95% CI: 95% confidence intervals.

Supplemental file 14. Data to accompany Figure 2. Children with maternal substance use problems in the first 1000 days of life, ascertained from different combinations of data sources, by child's year of birth

Year of birth	N[1]	Data source/s	N[2]	%	95% CI
2008	96,288	Child protection records[3]	3,936	4.09	3.96 - 4.21
2009	96,390	Child protection records	3,187	3.31	3.19 - 3.42
2010	96,427	Child protection records	2,337	2.42	2.33 - 2.52
2011	97,187	Child protection records	2,221	2.29	2.19 - 2.38
2012	99,458	Child protection records	2,357	2.37	2.28 - 2.46
2013	96,899	Child protection records	2,549	2.63	2.53 - 2.73
2014	97,279	Child protection records	2,566	2.64	2.54 - 2.74
2015	96,345	Child protection records	2,448	2.54	2.44 - 2.64
2016	98,472	Child protection records	2,256	2.29	2.20 - 2.38
2017	95,725	Child protection records	2,188	2.29	2.19 - 2.38
2008	96,288	Five health and death records[4]	1,616	1.68	1.60 - 1.76
2009	96,390	Five health and death records	1,356	1.41	1.33 - 1.48
2010	96,427	Five health and death records	1,414	1.47	1.39 - 1.54
2011	97,187	Five health and death records	1,381	1.42	1.35 - 1.5
2012	99,458	Five health and death records	1,430	1.44	1.36 - 1.51
2013	96,899	Five health and death records	1,371	1.41	1.34 - 1.49
2014	97,279	Five health and death records	1,419	1.46	1.38 - 1.53
2015	96,345	Five health and death records	1,407	1.46	1.38 - 1.54
2016	98,472	Five health and death records	1,321	1.34	1.27 - 1.41
2017	95,725	Five health and death records	1,260	1.32	1.24 - 1.39
2008	96,288	All six mother and child records[5]	4,557	4.73	4.60 - 4.87
2009	96,390	All six mother and child records	3,742	3.88	3.76 – 4.00
2010	96,427	All six mother and child records	3,026	3.14	3.03 - 3.25
2011	97,187	All six mother and child records	2,937	3.02	2.91 - 3.13
2012	99,458	All six mother and child records	3,060	3.08	2.97 - 3.18
2013	96,899	All six mother and child records	3,192	3.29	3.18 - 3.41
2014	97,279	All six mother and child records	3,253	3.34	3.23 - 3.46
2015	96,345	All six mother and child records	3,125	3.24	3.13 - 3.36
2016	98,472	All six mother and child records	2,913	2.96	2.85 - 3.06
2017	95,725	All six mother and child records	2,842	2.97	2.86 - 3.08

Footnotes: 1. Denominator value, the total number of children born each year; 2. Numerator values, the number of children with maternal substance use recorded in data source/s born each year; 3. The child protection data source includes maternal and other carer substance use 4. Substance use ascertained from maternal hospital, cause of death, mental health outpatients, opioid treatment registry and, emergency department, and child hospital and cause of death records; 5. Substance use ascertained from maternal hospital, cause of death, mental health outpatients, opioid treatment registry, emergency department and child protection records, and from child hospital, cause of death and child protection records. Definitions: 95% CI: 95% confidence intervals.

Supplemental file 15. Data to accompany Figure 3. Number and percentage of children born in NSW from 2008-2017 (N=970,470) with maternal substance use problems records during three ascertainment periods and six administrative data sources for children and mothers

Time window	Prenatal (Conception to <28 days post birth)			Early life (28 days post-birth to 2nd birthday)			First 1000 days[2] (Conception to 2nd birthday)		
Substance type	n[1]	%	95% CIs	n	%	95% CIs	n	%	95% CIs
**Any substance use**									
All six mother and child records[3]	13,987	1.44	1.42 - 1.46	26,198	2.70	2.67 - 2.73	32,647	3.36	3.33 - 3.40
Child protection records	10,434	1.08	1.05 - 1.1	20,556	2.12	2.09 - 2.15	26,045	2.68	2.65 - 2.72
Five health and death records[4]	7,089	0.73	0.71 - 0.75	9,396	0.97	0.95 - 0.99	13,975	1.44	1.42 - 1.46
**Alcohol use**									
All six mother and child records[3]	3,776	0.39	0.38 - 0.4	11,262	1.16	1.14 - 1.18	13,637	1.41	1.38 - 1.43
Child protection records	3,046	0.31	0.3 - 0.32	8,659	0.89	0.87 - 0.91	10,702	1.1	1.08 - 1.12
Five health and death records[4]	1,072	0.11	0.1 - 0.12	3,490	0.36	0.35 - 0.37	4,272	0.44	0.43 - 0.45
**Other drug use**									
All six mother and child records[3]	11,605	1.2	1.17 - 1.22	17,479	1.8	1.77 - 1.83	23,485	2.42	2.39 - 2.45
Child protection records	8,311	0.86	0.84 - 0.87	13,109	1.35	1.33 - 1.37	18,152	1.87	1.84 - 1.9
Five health and death records[4]	6,376	0.66	0.64 - 0.67	6,946	0.72	0.7 - 0.73	11,175	1.15	1.13 - 1.17

Definitions: 95% CI: 95% confidence intervals. 1. Numerator value/number of children with substance use recorded in data source/s. 2. The outcomes are not mutually exclusive across each time period; therefore, the number of children in the prenatal period and early life periods do not sum to the total number in the Frist 1000 days. 3. Substance use ascertained from maternal hospital, cause of death, mental health outpatients, opioid treatment registry, emergency department and child protection records; 4. Substance use ascertained from maternal hospital, cause of death, mental health outpatients, opioid treatment registry, and emergency department records, and child hospital and cause of death records.

Supplemental file 16. Sensitivity analysis: Number and percentage of children with maternal substance use problems in the first 1000 days, ascertained from six data sources, for children born in NSW from 2008-2017 (N=970,470) using additional ICD codes for substance use ascertainment from maternal hospital, mental health and cause of death records

Data source	Single data sources												Combined data sources			
	Cause of death[1]		Mental health outpatients		Opioid treatment register		Emergency department		Hospital		Child protection[2]		Health +/or cause of death[3]		Any data source[3]	
Substance type	n	%	n	%	n	%	n	%	n	%	n	%	n	%	n	%
**Maternal data sources** (5 data sources)																
Any substance[3]	<73	<0.1	1,042	0.1	3,229	0.3	2,264	0.2	10,998	1.1	-	-	-	-	13,201	1.4
Alcohol	-	-	511	0.1	-	-	1,431	0.2	3,046	0.3	-	-	-	-	4,246	0.4
Other drug[4]	-	-	562	<0.1	3,229	0.3	877	0.1	9,268	1.0	-	-	-	-	10,467	1.1
Opioid or opioid treatment	-	-	30	<0.1	3,229	0.3	144	<0.1	3,064	0.3	-	-	-	-	4,013	0.4
Cannabis	-	-	107	<0.1	-	-	43	<0.1	4,435	0.5	-	-	-	-	4,505	0.5
Stimulant	-	-	104	<0.1	-	-	62	<0.1	2,118	0.2	-	-	-	-	2,191	0.2
Sedatives or other[5]	-	-	23	<0.1	-	-	66	<0.1	1,020	0.1	-	-	-	-	1,088	0.1
Unspecified or multiple[6]	-	-	342	<0.1	-	-	618	<0.1	1,860	0.2	-	-	-	-	2,578	0.3
**Child data sources** (3 data sources)																
Any substance[3]	<5	<0.1	-	-	-	-	-	-	3,827	0.4	26,045	2.7	-	-	**27,327**	**2.8**
Alcohol	-	-	-	-	-	-	-	-	62	<0.1	10,702	1.1	-	-	**10,726**	**1.1**
Other drug[4]	-	-	-	-	-	-	-	-	3,787	0.4	18,152	1.9	-	-	**19,559**	**2.0**
**Child and maternal data sources combined** (6 data sources)[2]																
Any substance[3]	73	<0.1	1,042	0.1	3,229	0.3	2,264	0.2	12,272	1.3	26,045	2.7	**14,219**	**1.5**	**32,840**	**3.4**
Alcohol	-	-	511	0.1	-	-	1,431	0.2	3,074	0.3	10,702	1.1	**11,514**	**1.2**	**13,637**	**1.4**
Other drug[4]	-	-	562	0.1	3,229	0.3	877	0.1	10,564	1.1	18,152	1.9	**4,272**	**0.4**	**23,768**	**2.4**

1. For cause of death data, reporting is limited to the outcome "any substance use" due to low numbers in specific substance types; 2. The child protection data source includes maternal and other carer substance use; 3. The outcomes are not mutually exclusive across data sources or substance types; therefore, numbers in rows/columns do not sum to numbers reported in "All records and data sources" (rows) or the "Any substance use"outcome (columns); 3. Any substance use includes the use of alcohol and or any drug use; 4. Other drug use includes the use of drugs other than alcohol and tobacco, including illicit drugs, misuse of prescription drugs, and use of opioid agonist treatment; 5. "Sedatives or other" includes the use of sedatives, hallucinogens, sedatives, solvents, or organic compounds, which were aggregated due to small cell sizes in individual substance categories; 6. "Unspecified or multiple substance use" includes ICD-10-AM codes that indicate multiple drug use or do not specify the type of drug or psychoactive substance.

Supplemental file 17. Sensitivity analysis: number and percentage of children with maternal substance use problems in the first 1000 days, ascertained from five data sources, for children born in NSW from 2008-2017 (N=970,470)

Data source	Single data sources										Combined data sources			
	Cause of death[1]		Mental health outpatients		Emergency department		Hospital		Child protection[2]		Health +/or cause of death[3]		Any data source[3]	
Substance type	n	%	n	%	n	%	n	%	n	%	n	%	n	%
**Maternal data sources (5 data sources)**														
Any substance[4]	<64	<0.1	964	0.1	2,206	0.2	10,793	1.1	-	-	-	-	12,681	1.31
Alcohol	-	-	511	<0.1	1,431	0.1	3,046	0.3	-	-	-	-	4,246	0.44
Other drug[5]	-	-	473	<0.1	817	0.1	8,985	0.9	-	-	-	-	9,851	1.02
Cannabis	-	-	107	<0.1	43	<0.1	4,435	0.5	-	-	-	-	4,505	0.46
Opioid or opioid treatment	-	-	30	<0.1	144	<0.1	3,063	0.3	-	-	-	-	3,736	0.38
Stimulant	-	-	102	<0.1	56	<0.1	2,117	0.2	-	-	-	-	2,182	0.22
Sedatives or other[6]	-	-	6	<0.1	10	<0.1	549	0.1	-	-	-	-	572	0.06
Unspecified or multiple[7]	-	-	342	<0.1	618	0.1	1,858	0.2	-	-	-	-	2,576	0.27
**Child data sources (3 data sources)**														
Any substance[4]	<5	<0.1	-	-	-	-	3,827	0.4	26,045	2.7	-	-	27,327	2.82
Alcohol	-	-	-	-	-	-	62	<0.1	10,702	1.1	-	-	10,726	1.11
Other drug[5]	-	-	-	-	-	-	3,787	0.4	18,152	1.9	-	-	19,559	2.02
**Child and maternal data sources combined (6 data sources)[3]**														
Any substance[4]	64	<0.1	964	0.1	2,203	0.2	12,067	1.2	26,045	2.7	13,700	1.41	32,372	3.34
Alcohol	-	-	511	<0.1	1,431	0.1	3,074	0.3	10,702	1.1	11,175	1.15	13,637	1.41
Other drug[5]	-	-	473	<0.1	814	0.1	10,282	1.1	18,152	1.9	3,997	0.41	23,210	2.39

1. For cause of death data, reporting is limited to the outcome "any substance use" due to low numbers in specific substance types; 2. Child protection data includes all carers 3. The outcomes are not mutually exclusive across data sources or substance types; therefore, numbers in rows/columns do not sum to numbers reported in "All records and data sources" (rows) or the "Any substance use"outcome (columns); 4. Any substance use includes the use of alcohol and or any drug use; 5. Other drug use includes the use of drugs other than alcohol and tobacco, including illicit drugs, misuse of prescription drugs, and use of opioid agonist treatment; 6. "Sedatives or other" includes the use of sedatives, hallucinogens, sedatives, solvents, or organic compounds, which were aggregated due to small cell sizes in individual substance categories; 7. "Unspecified or multiple substance use" includes ICD10AM codes that indicate multiple drug use or do not specify the type of drug or psychoactive substance.

Supplemental file 18. Limitations of data sources used in analysis

Data source	Additional information, limitations and potential sources of bias
Perinatal data	• NSW perinatal data collection does not include children who are born very premature or underweight, and those who do not have a birth registration. However, both birth data sources have high population coverage and registration of births among disadvantaged populations has greatly improved over recent years.
NSW Department of Communities and Justice, child protection reports (child protection report data)	• Child protection records only represent a small number of the children who are maltreated and reported to child protection services, missing those children who are not reported.[1]<br>• Carer substance use may be under-ascertained in the prenatal period, as child protection records for prenatal reports do not included details on the reason for report, meaning that substance use could not be ascertained from *prenatal reports*. However, all children with a prenatal report in our study went on to have subsequent contact with child protection after pregnancy, when we were able to ascertain a reason for referral.<br>• Maternal substance use reported or assessed by child protection is included in carer drug and/or alcohol use issues recorded in child protection data; as such, it is not possible to differentiate maternal from other carer substance use from this data source. We addressed this limitation by: (i) noting the inclusion of carer substance use from child protection data in the maternal substance use outcome derived from health, death, and child protection data in results; (ii) using 'maternal and other carer' rather than 'maternal' substance use for estimates from child protection data alone; and (iii) conducting an additional analysis estimating the prevalence of maternal substance use derived only from health and death records.<br>• However, Australian data show in most cases of substantiated maltreatment the subjects of the investigation are mothers (79% of all neglect cases which includes carer substance use, 60% of all maltreatment cases).[2, 3] Furthermore, in this study at the time of birth 60% of the mothers in the maternal substance use group were single.
NSW Admitted Patient Data Collection (Hospital inpatient data)	• Surveillance bias may mean that some populations are more likely to be screened during pregnancy and at birth for substance use, such as women who absent, or present late to prenatal care, have low maternal age,[4, 5] or are among racially and ethnically minoritized populations.[5, 6]<br>• ICD-10-AM diagnostic codes recorded in hospital inpatient data underreport the prevalence of alcohol and other drug use disorders,[7] and also provide limited information on the modes of administration, dosage and potency of drugs used.
NSW Emergency Department Data Collection (emergency department presentations)	• Emergency records only report the primary presenting reason, missing other conditions that may be contributing to the person's emergency presentation.<br>• There is also variation in electronic medical record systems and practices across NSW emergency departments, resulting in variable diagnosis coding practices.<br>• Primary presenting reasons coded using SNOMED-CT coding systems can represent symptoms or investigations, and are not restricted to diagnoses.

	• The EDDC is not necessarily representative of all NSW emergency departments, as not all emergency departments contribute data to the emergency department data collection (EDDC). For example, only public hospitals contribute to the EDDC and less emergency departments in rural and regional areas contribute than in other areas. However, the proportion of participating emergency departments has increased, from around 30% in 1996 to around 60% in 2010.
NSW Mental Health Ambulatory Data Collection (Mental health outpatient data)	• Mental health outpatients' appointments only represent the publicly funded mental health services, missing a significant market of private health providers and services.
NSW Cause of Death Unit Record File Unit Record File (death registrations)	• Substance use may be under-ascertained in cause of death records as coroners do not routinely screen for all substances (e.g., volatile solvents) unless there is reason to suspect their use.[8]

References

1. Fallon B, Trocmé N, Fluke J, MacLaurin B, Tonmyr L, Yuan Y-Y. Methodological challenges in measuring child maltreatment. Child abuse & neglect. 2010;34(1):70-9.
2. Child Family Community Australia. Who abuses children? Melborune; 2014.
3. Hurren E, Thompson C, Jenkins B, Chrzanowski A, Allard T, Stewart A. Who are the Perpetrators of Child Maltreatment? Report to the Criminology Research Advisory Council.; 2018.
4. Drescher Burke K. Substance-exposed newborns: Hospital and child protection responses. Children and Youth Services Review. 2007;29(12):1503-19.
5. Patel E, Bandara S, Saloner B, Stuart EA, Goodman D, Terplan M, et al. Heterogeneity in prenatal substance use screening despite universal screening recommendations: findings from the Pregnancy Risk Assessment Monitoring System, 2016-2018. Am J Obstet Gynecol MFM. 2021;3(5):100419.
6. Tucker Edmonds B. Mandated Reporting of Perinatal Substance Use: The Root of Inequity. JAMA Pediatr. 2022;176(11):1073-5.
7. Williams EC, Fletcher OV, Frost MC, Harris AHS, Washington DL, Hoggatt KJ. Comparison of Substance Use Disorder Diagnosis Rates From Electronic Health Record Data With Substance Use Disorder Prevalence Rates Reported in Surveys Across Sociodemographic Groups in the Veterans Health Administration. JAMA Network Open. 2022;5(6):e2219651-e.
8. Darke S, Zahra E, Duflou J, Peacock A, Farrell M, Lappin J. Characteristics and circumstances of volatile solvent misuse-related death in Australia, 2000–2021. Clinical Toxicology. 2023;61(4):260-5.
