# Supplementary material for: Prevalence of maternal substance use problems during pregnancy and the first 2 years of life: a whole-population birth cohort of 970 470 Australian children born 2008–2017
Source: J Epidemiol Community Health. 2025 Mar 24;79(8):e223439. doi: 10.1136/jech-2024-223439 (PMC12322432; doi:10.1136/jech-2024-223439)
Supplement: online supplemental file 2 [file jech-79-8-s002.pdf]

Supplemental file 6a. Definitions of ICD-10-AM and/or SNOMED CT[1] codes used to ascertain maternal substance use from four data sources available in the NSW child e-cohort

Code description	Code	Analysis	Substance type
**ICD-10 AM codes**			
**Child records**			
P96.1 Neonatal withdrawal symptoms from maternal use of drugs of addiction	P96.1	Main analysis	Other drug
P04.4 Fetus and newborn affected by maternal use of drugs of addiction	P04.4	Main analysis	Other drug
P04.3 Fetus and newborn affected by maternal use of alcohol	P04.3	Main analysis	Alcohol
Q86.0 Fetal alcohol syndrome (dysmorphic)	Q86.0	Main analysis	Alcohol
**Mother records**			
**Alcohol**			
O35.4 Maternal care for (suspected) damage to fetus from alcohol	O35.4	Main analysis	Alcohol
Z50.2 Alcohol rehabilitation	Z50.2	Main analysis	Alcohol
Z72.1 Alcohol use	Z72.1	Main analysis	Alcohol
R78.0 Finding of alcohol in blood	R78.0	Main analysis	Alcohol
Y90 Evidence of alcohol involvement determined by blood alcohol level	Y90	Main analysis	Alcohol
Y90.0 Blood alcohol level of less than 20 mg/100 ml	Y90.0	Main analysis	Alcohol
Y90.1 Blood alcohol level of 20-39 mg/100 ml	Y90.1	Main analysis	Alcohol
Y90.2 Blood alcohol level of 40-59 mg/100 ml	Y90.2	Main analysis	Alcohol
Y90.3 Blood alcohol level of 60-79 mg/100 ml	Y90.3	Main analysis	Alcohol
Y90.4 Blood alcohol level of 80-99 mg/100 ml	Y90.4	Main analysis	Alcohol
Y90.5 Blood alcohol level of 100-119 mg/100 ml	Y90.5	Main analysis	Alcohol
Y90.6 Blood alcohol level of 120-199 mg/100 ml	Y90.6	Main analysis	Alcohol
Y90.7 Blood alcohol level of 200-239 mg/100 ml	Y90.7	Main analysis	Alcohol
Y90.8 Blood alcohol level of 240 mg/100 ml or more	Y90.8	Main analysis	Alcohol
Y90.9 Presence of alcohol in blood, level not specified	Y90.9	Main analysis	Alcohol
Y91 Evidence of alcohol involvement determined by level of intoxication	Y91	Main analysis	Alcohol
Y91.0 Mild alcohol intoxication	Y91.0	Main analysis	Alcohol
Y91.1 Moderate alcohol intoxication	Y91.1	Main analysis	Alcohol
Y91.2 Severe alcohol intoxication	Y91.2	Main analysis	Alcohol
Y91.3 Very severe alcohol intoxication	Y91.3	Main analysis	Alcohol
Y91.9 Alcohol involvement, not otherwise specified	Y91.9	Main analysis	Alcohol
T51 Toxic effect of alcohol	T51	Main analysis	Alcohol
T51.0 Ethanol	T51.0	Main analysis	Alcohol
T51.1 Methanol	T51.1	Main analysis	Alcohol
T51.2 2-Propanol	T51.2	Main analysis	Alcohol
T51.8 Other alcohols	T51.8	Main analysis	Alcohol
T51.9 Alcohol, unspecified	T51.9	Main analysis	Alcohol
X45 Accidental poisoning by and exposure to alcohol	X45	Main analysis	Alcohol

Code description	Code	Analysis	Substance type
X65 Intentional self-poisoning by and exposure to alcohol	X65	Main analysis	Alcohol
Y15 Poisoning by and exposure to alcohol, undetermined intent	Y15	Main analysis	Alcohol
F10 Mental and behavioural disorders due to use of alcohol	F10	Main analysis	Alcohol
F10.0 Mental and behavioural disorders due to use of alcohol, acute intoxication	F10.0	Main analysis	Alcohol
F10.1 Mental and behavioural disorders due to use of alcohol, harmful use	F10.1	Main analysis	Alcohol
F10.2 Mental and behavioural disorders due to use of alcohol, dependence syndrome	F10.2	Main analysis	Alcohol
F10.3 Mental and behavioural disorders due to use of alcohol, withdrawal state	F10.3	Main analysis	Alcohol
F10.4 Mental and behavioural disorders due to use of alcohol, withdrawal state with delirium	F10.4	Main analysis	Alcohol
F10.5 Mental and behavioural disorders due to use of alcohol, psychotic disorder	F10.5	Main analysis	Alcohol
F10.6 Mental and behavioural disorders due to use of alcohol, amnesic syndrome	F10.6	Main analysis	Alcohol
F10.7 Mental and behavioural disorders due to use of alcohol, residual and late-onset psychotic disorder	F10.7	Main analysis	Alcohol
F10.8 Mental and behavioural disorders due to use of alcohol, other mental and behavioural disorders	F10.8	Main analysis	Alcohol
F10.9 Mental and behavioural disorders due to use of alcohol, unspecified mental and behavioural disorder	F10.9	Main analysis	Alcohol
**Opioids**			
T40.0 Opium	T40.0	Main analysis	Opioids
T40.1 Heroin	T40.1	Main analysis	Opioids
T40.2 Other opioids	T40.2	Main analysis	Opioids
T40.3 Methadone	T40.3	Main analysis	Opioids
F11 Mental and behavioural disorders due to use of opioids	F11	Main analysis	Opioids
F11.0 Mental and behavioural disorders due to use of opioids, acute intoxication	F11.0	Main analysis	Opioids
F11.1 Mental and behavioural disorders due to use of opioids, harmful use	F11.1	Main analysis	Opioids
F11.2 Mental and behavioural disorders due to use of opioids, dependence syndrome	F11.2	Main analysis	Opioids
F11.3 Mental and behavioural disorders due to use of opioids, withdrawal state	F11.3	Main analysis	Opioids
F11.4 Mental and behavioural disorders due to use of opioids, withdrawal state with delirium	F11.4	Main analysis	Opioids
F11.5 Mental and behavioural disorders due to use of opioids, psychotic disorder	F11.5	Main analysis	Opioids
F11.6 Mental and behavioural disorders due to use of opioids, amnesic syndrome	F11.6	Main analysis	Opioids
F11.7 Mental and behavioural disorders due to use of opioids, residual and late-onset psychotic disorder	F11.7	Main analysis	Opioids
F11.8 Mental and behavioural disorders due to use of opioids, other mental and behavioural disorders	F11.8	Main analysis	Opioids
F11.9 Mental and behavioural disorders due to use of opioids, unspecified mental and behavioural disorder	F11.9	Main analysis	Opioids
R78.1 Finding of opiate drug in blood	R78.1	Sensitivity analysis	Opioids
**Cannabis/Cannabinoids**			
T40.7 Cannabis (derivatives)	T40.7	Main analysis	Cannabis
F12 Mental and behavioural disorders due to use of cannabinoids	F12	Main analysis	Cannabis
F12.0 Mental and behavioural disorders due to use of cannabinoids, acute intoxication	F12.0	Main analysis	Cannabis
F12.1 Mental and behavioural disorders due to use of cannabinioids, harmful use	F12.1	Main analysis	Cannabis
F12.2 Mental and behavioural disorders due to use of cannabinioids, dependence syndrome	F12.2	Main analysis	Cannabis
F12.3 Mental and behavioural disorders due to use of cannabinioids, withdrawal state	F12.3	Main analysis	Cannabis
F12.4 Mental and behavioural disorders due to use of cannabinioids, withdrawal state with delirium	F12.4	Main analysis	Cannabis

Code description	Code	Analysis	Substance type
F12.5 Mental and behavioural disorders due to use of cannabinioids, psychotic disorder	F12.5	Main analysis	Cannabis
F12.6 Mental and behavioural disorders due to use of cannabinioids, amnesic syndrome	F12.6	Main analysis	Cannabis
F12.7 Mental and behavioural disorders due to use of cannabinioids, residual and late-onset psychotic disorder	F12.7	Main analysis	Cannabis
F12.8 Mental and behavioural disorders due to use of cannabinioids, other mental and behavioural disorders	F12.8	Main analysis	Cannabis
F12.9 Mental and behavioural disorders due to use of cannabinioids, unspecified mental and behavioural disorder	F12.9	Main analysis	Cannabis
**STIMULANTS**			
**Cocaine**			
R78.2 Finding of cocaine in blood	R78.2	Main analysis	Stimulants
T40.5 Cocaine	T40.5	Main analysis	Stimulants
F14 Mental and behavioural disorders due to use of cocaine	F14	Main analysis	Stimulants
F14.0Mental and behavioural disorders due to use of cocaine, acute intoxication	F14.0	Main analysis	Stimulants
F14.1 Mental and behavioural disorders due to use of cocaine, harmful use	F14.1	Main analysis	Stimulants
F14.2 Mental and behavioural disorders due to use of cocaine, dependence syndrome	F14.2	Main analysis	Stimulants
F14.3 Mental and behavioural disorders due to use of cocaine, withdrawal state	F14.3	Main analysis	Stimulants
F14.4 Mental and behavioural disorders due to use of cocaine, withdrawal state with delirium	F14.4	Main analysis	Stimulants
F14.5 Mental and behavioural disorders due to use of cocaine, psychotic disorder	F14.5	Main analysis	Stimulants
F14.6 Mental and behavioural disorders due to use of cocaine, amnesic syndrome	F14.6	Main analysis	Stimulants
F14.7 Mental and behavioural disorders due to use of cocaine, residual and late-onset psychotic disorder	F14.7	Main analysis	Stimulants
F14.8 Mental and behavioural disorders due to use of cocaine, other mental and behavioural disorders	F14.8	Main analysis	Stimulants
F14.9 Mental and behavioural disorders due to use of cocaine, unspecified mental and behavioural disorder	F14.9	Main analysis	Stimulants
**Other stimulants**			
T43.69 Other psychostimulants with potential for use disorder	T43.69	Main analysis	Stimulants
F15 Mental and behavioural disorders due to use other stimulants, including caffeine	F15	Main analysis	Stimulants
F15.0 Mental and behavioural disorders due to use other stimulants including caffeine, acute intoxication	F15.0	Main analysis	Stimulants
F15.00 Mental and behavioural disorders due to use other stimulants including caffeine, acute intoxication, unspecified stimulants	F15.00	Main analysis	Stimulants
F15.01 Mental and behavioural disorders due to use other stimulants including caffeine, acute intoxication, methylamphetamine	F15.01	Main analysis	Stimulants
F15.02 Mental and behavioural disorders due to use other stimulants including caffeine, acute intoxication, methylenedioxy methylamphetamine	F15.02	Main analysis	Stimulants
F15.09 Mental and behavioural disorders due to use other stimulants including caffeine, acute intoxication, other specified stimulants	F15.09	Main analysis	Stimulants
F15.1 Mental and behavioural disorders due to use other stimulants including caffeine, harmful use	F15.1	Main analysis	Stimulants
F15.10 Mental and behavioural disorders due to use other stimulants including caffeine, harmful use, unspecified stimulants	F15.10	Main analysis	Stimulants
F15.11 Mental and behavioural disorders due to use other stimulants including caffeine, harmful use, methylamphetamine	F15.11	Main analysis	Stimulants
F15.12 Mental and behavioural disorders due to use other stimulants including caffeine, harmful use, methylenedioxy methylamphetamine	F15.12	Main analysis	Stimulants
F15.19 Mental and behavioural disorders due to use other stimulants including caffeine, harmful use, other specified stimulants	F15.19	Main analysis	Stimulants
F15.2 Mental and behavioural disorders due to use other stimulants including caffeine, dependence syndrome	F15.2	Main analysis	Stimulants
F15.20 Mental and behavioural disorders due to use other stimulants including caffeine, dependence syndrome, unspecified stimulants	F15.20	Main analysis	Stimulants
F15.21 Mental and behavioural disorders due to use other stimulants including caffeine, dependence syndrome, methylamphetamine	F15.21	Main analysis	Stimulants

Code description	Code	Analysis	Substance type
F15.22 Mental and behavioural disorders due to use other stimulants including caffeine, dependence syndrome, methylenedioxy methylamphetamine	F15.22	Main analysis	Stimulants
F15.29 Mental and behavioural disorders due to use other stimulants including caffeine, dependence syndrome, other specified stimulants	F15.29	Main analysis	Stimulants
F15.3 Mental and behavioural disorders due to use other stimulants including caffeine, withdrawal state	F15.3	Main analysis	Stimulants
F15.30 Mental and behavioural disorders due to use other stimulants including caffeine, withdrawal state, unspecified stimulants	F15.30	Main analysis	Stimulants
F15.31 Mental and behavioural disorders due to use other stimulants including caffeine, withdrawal state, methylamphetamine	F15.31	Main analysis	Stimulants
F15.32 Mental and behavioural disorders due to use other stimulants including caffeine, withdrawal state, methylenedioxy methylamphetamine	F15.32	Main analysis	Stimulants
F15.39 Mental and behavioural disorders due to use other stimulants including caffeine, withdrawal state, other specified stimulants	F15.39	Main analysis	Stimulants
F15.4 Mental and behavioural disorders due to use other stimulants including caffeine, withdrawal state with delirium	F15.4	Main analysis	Stimulants
F15.40 Mental and behavioural disorders due to use other stimulants including caffeine, withdrawal state with delirium, unspecified stimulants	F15.40	Main analysis	Stimulants
F15.41 Mental and behavioural disorders due to use other stimulants including caffeine, withdrawal state with delirium, methylamphetamine	F15.41	Main analysis	Stimulants
F15.42 Mental and behavioural disorders due to use other stimulants including caffeine, withdrawal state with delirium, methylamphetamine	F15.42	Main analysis	Stimulants
F15.49 Mental and behavioural disorders due to use other stimulants including caffeine, withdrawal state with delirium, other specified stimulants	F15.49	Main analysis	Stimulants
F15.5Mental and behavioural disorders due to use other stimulants including caffeine, psychotic disorder	F15.5	Main analysis	Stimulants
F15.50 Mental and behavioural disorders due to use other stimulants including caffeine, psychotic disorder, unspecified stimulants	F15.50	Main analysis	Stimulants
F15.51 Mental and behavioural disorders due to use other stimulants including caffeine, psychotic disorder, methylamphetamine	F15.51	Main analysis	Stimulants
F15.52 Mental and behavioural disorders due to use other stimulants including caffeine, psychotic disorder, methylenedioxy methylamphetamine	F15.52	Main analysis	Stimulants
F15.59 Mental and behavioural disorders due to use other stimulants including caffeine, psychotic disorder, other specified stimulants	F15.59	Main analysis	Stimulants
F15.6 Mental and behavioural disorders due to use other stimulants including caffeine, amnesic syndrome	F15.6	Main analysis	Stimulants
F15.60 Mental and behavioural disorders due to use other stimulants including caffeine, amnesic syndrome, unspecified stimulants	F15.60	Main analysis	Stimulants
F15.61 Mental and behavioural disorders due to use other stimulants including caffeine, amnesic syndrome, methlyamphetamine	F15.61	Main analysis	Stimulants
F15.62 Mental and behavioural disorders due to use other stimulants including caffeine, amnesic syndrome, methylenedioxy methlyamphetamine	F15.62	Main analysis	Stimulants
F15.69 Mental and behavioural disorders due to use other stimulants including caffeine, amnesic syndrome, other specified stimulants	F15.69	Main analysis	Stimulants
F15.7Mental and behavioural disorders due to use other stimulants including caffeine, residual and late-onset psychotic disorder	F15.7	Main analysis	Stimulants
F15.70 Mental and behavioural disorders due to use other stimulants including caffeine, residual and late-onset psychotic disorder, unspecified stimulants	F15.70	Main analysis	Stimulants
F15.71 Mental and behavioural disorders due to use other stimulants including caffeine, residual and late-onset psychotic disorder, methylamphetamine	F15.71	Main analysis	Stimulants
F15.72 Mental and behavioural disorders due to use other stimulants including caffeine, residual and late-onset psychotic disorder, methylenedioxy methylamphetamine	F15.72	Main analysis	Stimulants
F15.79 Mental and behavioural disorders due to use other stimulants including caffeine, residual and late-onset psychotic disorder, other specified stimulants	F15.79	Main analysis	Stimulants
F15.8 Mental and behavioural disorders due to use other stimulants including caffeine, other mental and behavioural disorders	F15.8	Main analysis	Stimulants

Code description	Code	Analysis	Substance type
F15.80 Mental and behavioural disorders due to use other stimulants including caffeine, other mental and behavioural disorders, unspecified stimulants	F15.80	Main analysis	Stimulants
F15.81 Mental and behavioural disorders due to use other stimulants including caffeine, other mental and behavioural disorders, methylamphetamine	F15.81	Main analysis	Stimulants
F15.82 Mental and behavioural disorders due to use other stimulants including caffeine, other mental and behavioural disorders, methylenedioxy methylamphetamine	F15.82	Main analysis	Stimulants
F15.89 Mental and behavioural disorders due to use other stimulants including caffeine, other mental and behavioural disorders, other specified stimulants	F15.89	Main analysis	Stimulants
F15.9 Mental and behavioural disorders due to use other stimulants including caffeine, unspecified mental and behavioural disorder	F15.9	Main analysis	Stimulants
F15.90 Mental and behavioural disorders due to use other stimulants including caffeine, unspecified mental and behavioural disorder, unspecified stimulants	F15.90	Main analysis	Stimulants
F15.91 Mental and behavioural disorders due to use other stimulants including caffeine, unspecified mental and behavioural disorder, methylamphetamine	F15.91	Main analysis	Stimulants
F15.92 Mental and behavioural disorders due to use other stimulants including caffeine, unspecified mental and behavioural disorder, methylenedioxy methylamphetamine	F15.92	Main analysis	Stimulants
F15.99 Mental and behavioural disorders due to use other stimulants including caffeine, unspecified mental and behavioural disorder, other specified stimulants	F15.99	Main analysis	Stimulants
T43.6 Psychostimulants with potential for use disorder	T43.6	Sensitivity analysis	Stimulants
T43.60 Unspecified psychostimulants with potential for use disorder	T43.60	Sensitivity analysis	Stimulants
**Methamphetamines**			
T43.61 Methylamphetamine	T43.61	Main analysis	Stimulants
T43.62 Methylenedioxy methamphetamine	T43.62	Main analysis	Stimulants
**Sedatives or Other**			
**Sedatives, and hypnotics,**			
F13 Mental and behavioural disorders due to use of sedatives or hypnotics	F13	Main analysis	Sedatives and others
F13.0 Mental and behavioural disorders due to use of sedatives or hypnotics, acute intoxication	F13.0	Main analysis	Sedatives and others
F13.00 Mental and behavioural disorders due to use of sedatives or hypnotics, acute intoxication, unspecified sedative or hypnotic	F13.00	Main analysis	Sedatives and others
F13.01 Mental and behavioural disorders due to use of sedatives or hypnotics, acute intoxication, gamma hydroxybutyrate	F13.01	Main analysis	Sedatives and others
F13.09 Mental and behavioural disorders due to use of sedatives or hypnotics, acute intoxication, other specified sedative or hypnotic	F13.09	Main analysis	Sedatives and others
F13.1 Mental and behavioural disorders due to use of sedatives or hypnotics, harmful use	F13.1	Main analysis	Sedatives and others
F13.10 Mental and behavioural disorders due to use of sedatives or hypnotics, harmful use, unspecified sedative or hypnotic	F13.10	Main analysis	Sedatives and others
F13.11 Mental and behavioural disorders due to use of sedatives or hypnotics, harmful use, gamma hydroxybutyrate	F13.11	Main analysis	Sedatives and others
F13.19 Mental and behavioural disorders due to use of sedatives or hypnotics, harmful use, other specified sedative or hypnotic	F13.19	Main analysis	Sedatives and others
F13.2 Mental and behavioural disorders due to use of sedatives or hypnotics, dependence syndrome	F13.2	Main analysis	Sedatives and others
F13.20 Mental and behavioural disorders due to use of sedatives or hypnotics, dependence syndrome, unspecified sedative or hypnotic	F13.20	Main analysis	Sedatives and others
F13.21 Mental and behavioural disorders due to use of sedatives or hypnotics, dependence syndrome, gamma hydroxybutyrate	F13.21	Main analysis	Sedatives and others
F13.29 Mental and behavioural disorders due to use of sedatives or hypnotics, dependence syndrome, other specified sedative or hypnotic	F13.29	Main analysis	Sedatives and others
F13.3Mental and behavioural disorders due to use of sedatives or hypnotics, withdrawal state	F13.3	Main analysis	Sedatives and others
F13.30 Mental and behavioural disorders due to use of sedatives or hypnotics, withdrawal state, unspecified sedative or hypnotic	F13.30	Main analysis	Sedatives and others

Code description	Code	Analysis	Substance type
F13.31 Mental and behavioural disorders due to use of sedatives or hypnotics, withdrawal state, gamma hydroxybutyrate	F13.31	Main analysis	Sedatives and others
F13.39 Mental and behavioural disorders due to use of sedatives or hypnotics, withdrawal state, other specified sedative or hypnotic	F13.39	Main analysis	Sedatives and others
F13.4 Mental and behavioural disorders due to use of sedatives or hypnotics, withdrawal state with delirium	F13.4	Main analysis	Sedatives and others
F13.40 Mental and behavioural disorders due to use of sedatives or hypnotics, withdrawal state with delirium, unspecified sedative or hypnotic	F13.40	Main analysis	Sedatives and others
F13.41 Mental and behavioural disorders due to use of sedatives or hypnotics, withdrawal state with delirium, gamma hydroxybutyrate	F13.41	Main analysis	Sedatives and others
F13.49 Mental and behavioural disorders due to use of sedatives or hypnotics, withdrawal state with delirium, other specified sedative or hypnotic	F13.49	Main analysis	Sedatives and others
F13.5Mental and behavioural disorders due to use of sedatives or hypnotics, psychotic disorder	F13.5	Main analysis	Sedatives and others
F13.50 Mental and behavioural disorders due to use of sedatives or hypnotics, psychotic disorder, unspecified sedative or hypnotic	F13.50	Main analysis	Sedatives and others
F13.51 Mental and behavioural disorders due to use of sedatives or hypnotics, psychotic disorder, gamma hydroxybutyrate	F13.51	Main analysis	Sedatives and others
F13.59 Mental and behavioural disorders due to use of sedatives or hypnotics, psychotic disorder, other specified sedative or hypnotic	F13.59	Main analysis	Sedatives and others
F13.6 Mental and behavioural disorders due to use of sedatives or hypnotics, amnesic syndrome	F13.6	Main analysis	Sedatives and others
F13.60 Mental and behavioural disorders due to use of sedatives or hypnotics, amnesic syndrome, unspecified sedative or hypnotic	F13.60	Main analysis	Sedatives and others
F13.61 Mental and behavioural disorders due to use of sedatives or hypnotics, amnesic syndrome, gamma hydroxybutyrate	F13.61	Main analysis	Sedatives and others
F13.69 Mental and behavioural disorders due to use of sedatives or hypnotics, amnesic syndrome, other specified sedative or hypnotic	F13.69	Main analysis	Sedatives and others
F13.7Mental and behavioural disorders due to use of sedatives or hypnotics, residual and late-onset psychotic disorder	F13.7	Main analysis	Sedatives and others
F13.70 Mental and behavioural disorders due to use of sedatives or hypnotics, residual and late-onset psychotic disorder, unspecified sedative or hypnotic	F13.70	Main analysis	Sedatives and others
F13.71 Mental and behavioural disorders due to use of sedatives or hypnotics, residual and late-onset psychotic disorder, gamma hydroxybutyrate	F13.71	Main analysis	Sedatives and others
F13.79 Mental and behavioural disorders due to use of sedatives or hypnotics, residual and late-onset psychotic disorder, other specified sedative or hypnotic	F13.79	Main analysis	Sedatives and others
F13.8 Mental and behavioural disorders due to use of sedatives or hypnotics, other mental and behavioural disorders	F13.8	Main analysis	Sedatives and others
F13.80 Mental and behavioural disorders due to use of sedatives or hypnotics, other mental and behavioural disorders, unspecified sedative or hypnotic	F13.80	Main analysis	Sedatives and others
F13.81 Mental and behavioural disorders due to use of sedatives or hypnotics, other mental and behavioural disorders, gamma hydroxybutyrate	F13.81	Main analysis	Sedatives and others
F13.89 Mental and behavioural disorders due to use of sedatives or hypnotics, other mental and behavioural disorders, other specified sedative or hypnotic	F13.89	Main analysis	Sedatives and others
F13.9 Mental and behavioural disorders due to use of sedatives or hypnotics, unspecified mental and behavioural disorder	F13.9	Main analysis	Sedatives and others
F13.90 Mental and behavioural disorders due to use of sedatives or hypnotics, unspecified mental and behavioural disorder, unspecified sedative or hypnotic	F13.90	Main analysis	Sedatives and others
F13.91 Mental and behavioural disorders due to use of sedatives or hypnotics, unspecified mental and behavioural disorder, gamma hydroxybutyrate	F13.91	Main analysis	Sedatives and others
F13.99 Mental and behavioural disorders due to use of sedatives or hypnotics, unspecified mental and behavioural disorder, other specified sedative or hypnotic	F13.99	Main analysis	Sedatives and others
T42 Poisoning by antiepileptic, sedative-hypnotic and antiparkinsonism drugs	T42	Sensitivity analysis	Sedatives and others
T42.6 Other antiepileptic and sedative-hypnotic drugs	T42.6	Sensitivity analysis	Sedatives and others
T42.7 Antiepileptic and sedative-hypnotic drugs, unspecified	T42.7	Sensitivity analysis	Sedatives and others
T42.3 Barbiturates	T42.3	Sensitivity analysis	Sedatives and others

Code description	Code	Analysis	Substance type
T42.4 Benzodiazepines	T42.4	Sensitivity analysis	Sedatives and others
**Hallucinogens**			
R78.3 Finding of hallucinogen in blood	R78.3	Main analysis	Sedatives and others
T40.9 Other and unspecified psychodysleptics [hallucinogens]	T40.9	Main analysis	Sedatives and others
F16 Mental and behavioural disorders due to use hallucinogens	F16	Main analysis	Sedatives and others
F16.0 Mental and behavioural disorders due to use hallucinogens, acute intoxication	F16.0	Main analysis	Sedatives and others
F16.00 Mental and behavioural disorders due to use hallucinogens, acute intoxication, unspecified hallucinogen	F16.00	Main analysis	Sedatives and others
F16.01 Mental and behavioural disorders due to use hallucinogens, acute intoxication, ketamine	F16.01	Main analysis	Sedatives and others
F16.09 Mental and behavioural disorders due to use hallucinogens, acute intoxication, other specified hallucinogen	F16.09	Main analysis	Sedatives and others
F16.1 Mental and behavioural disorders due to use hallucinogens, harmful use	F16.1	Main analysis	Sedatives and others
F16.10 Mental and behavioural disorders due to use hallucinogens, harmful use, unspecified hallucinogen	F16.10	Main analysis	Sedatives and others
F16.11 Mental and behavioural disorders due to use hallucinogens, harmful use, ketamine	F16.11	Main analysis	Sedatives and others
F16.19 Mental and behavioural disorders due to use hallucinogens, harmful use, other specified hallucinogen	F16.19	Main analysis	Sedatives and others
F16.2 Mental and behavioural disorders due to use hallucinogens, dependence syndrome	F16.2	Main analysis	Sedatives and others
F16.20 Mental and behavioural disorders due to use hallucinogens, dependence syndrome, unspecified hallucinogen	F16.20	Main analysis	Sedatives and others
F16.21 Mental and behavioural disorders due to use hallucinogens, dependence syndrome, ketamine	F16.21	Main analysis	Sedatives and others
F16.29 Mental and behavioural disorders due to use hallucinogens, dependence syndrome, other specified hallucinogen	F16.29	Main analysis	Sedatives and others
F16.3 Mental and behavioural disorders due to use hallucinogens, withdrawal state	F16.3	Main analysis	Sedatives and others
F16.30 Mental and behavioural disorders due to use hallucinogens, withdrawal state, unspecified hallucinogen	F16.30	Main analysis	Sedatives and others
F16.31 Mental and behavioural disorders due to use hallucinogens, withdrawal state, Ketamine	F16.31	Main analysis	Sedatives and others
F16.39 Mental and behavioural disorders due to use hallucinogens, withdrawal state, other specified hallucinogen	F16.39	Main analysis	Sedatives and others
F16.4 Mental and behavioural disorders due to use hallucinogens, withdrawal state with delirium	F16.4	Main analysis	Sedatives and others
F16.40 Mental and behavioural disorders due to use hallucinogens, withdrawal state with delirium, unspecified hallucinogen	F16.40	Main analysis	Sedatives and others
F16.41 Mental and behavioural disorders due to use hallucinogens, withdrawal state with delirium, ketamine	F16.41	Main analysis	Sedatives and others
F16.49 Mental and behavioural disorders due to use hallucinogens, withdrawal state with delirium, other specified hallucinogen	F16.49	Main analysis	Sedatives and others
F16.5 Mental and behavioural disorders due to use hallucinogens, psychotic disorder	F16.5	Main analysis	Sedatives and others
F16.50 Mental and behavioural disorders due to use hallucinogens, psychotic disorder, unspecified hallucinogen	F16.50	Main analysis	Sedatives and others
F16.51 Mental and behavioural disorders due to use hallucinogens, psychotic disorder, ketamine	F16.51	Main analysis	Sedatives and others
F16.59 Mental and behavioural disorders due to use hallucinogens, psychotic disorder, other specified hallucinogen	F16.59	Main analysis	Sedatives and others
F16.6 Mental and behavioural disorders due to use hallucinogens, amnesic syndrome	F16.6	Main analysis	Sedatives and others
F16.60 Mental and behavioural disorders due to use hallucinogens, amnesic syndrome, unspecified hallucinogen	F16.60	Main analysis	Sedatives and others
F16.61 Mental and behavioural disorders due to use hallucinogens, amnesic syndrome, ketamine	F16.61	Main analysis	Sedatives and others
F16.69 Mental and behavioural disorders due to use hallucinogens, amnesic syndrome, other specified hallucinogen	F16.69	Main analysis	Sedatives and others
F16.7 Mental and behavioural disorders due to use hallucinogens, residual and late-onset psychotic disorder	F16.7	Main analysis	Sedatives and others
F16.70 Mental and behavioural disorders due to use hallucinogens, residual and late-onset psychotic disorder, unspecified hallucinogen	F16.70	Main analysis	Sedatives and others
F16.71 Mental and behavioural disorders due to use hallucinogens, residual and late-onset psychotic disorder, ketamine	F16.71	Main analysis	Sedatives and others
F16.79 Mental and behavioural disorders due to use hallucinogens, residual and late-onset psychotic disorder, other specified hallucinogen	F16.79	Main analysis	Sedatives and others
F16.8 Mental and behavioural disorders due to use hallucinogens, other mental and behavioural disorders	F16.8	Main analysis	Sedatives and others

Code description	Code	Analysis	Substance type
F16.80 Mental and behavioural disorders due to use hallucinogens, other mental and behavioural disorders, unspecified hallucinogen	F16.80	Main analysis	Sedatives and others
F16.81 Mental and behavioural disorders due to use hallucinogens, other mental and behavioural disorders, ketamine	F16.81	Main analysis	Sedatives and others
F16.89 Mental and behavioural disorders due to use hallucinogens, other mental and behavioural disorders, other specified hallucinogen	F16.89	Main analysis	Sedatives and others
F16.9 Mental and behavioural disorders due to use hallucinogens, unspecified mental and behavioural disorder	F16.9	Main analysis	Sedatives and others
F16.91 Mental and behavioural disorders due to use hallucinogens, unspecified mental and behavioural disorder, ketamine	F16.91	Main analysis	Sedatives and others
F16.99 Mental and behavioural disorders due to use hallucinogens, unspecified mental and behavioural disorder, other specified hallucinogen	F16.99	Main analysis	Sedatives and others
T40.8 Lysergide [LSD]	T40.8	Main analysis	Sedatives and others
T41.22 Ketamine	T41.22	Sensitivity analysis	Sedatives and others
**Unspecified or multiple**			
**Drug unspecified or multiple**			
O35.5 Maternal care for (suspected) damage to fetus by drugs ("drug addiction" in WHO)	O35.5	Main analysis	Drug unspecified or multiple
Z50.3 Drug rehabilitation	Z50.3	Main analysis	Drug unspecified or multiple
Z72.2 Drug use	Z72.2	Main analysis	Drug unspecified or multiple
R78 Findings of drugs and other substances, not normally found in blood	R78	Main analysis	Drug unspecified or multiple
R78.4 Finding of other drugs of addictive potential in blood	R78.4	Sensitivity analysis	Drug unspecified or multiple
**Psychotropic drug**			
R78.5 Finding of psychotropic drug in blood	R78.5	Sensitivity analysis	Drug unspecified or multiple
T43.9 Psychotropic drug, unspecified	T43.9	Sensitivity analysis	Drug unspecified or multiple
T43 Poisoning by psychotropic drugs, not elsewhere classified	T43	Sensitivity analysis	Drug unspecified or multiple
T43.8 Other psychotropic drugs, not elsewhere classified	T43.8	Sensitivity analysis	Drug unspecified or multiple
**Multiple drug**			
F19 Mental and behavioural disorders due to multiple drug use and use other psychoactive substances	F19	Main analysis	Drug unspecified or multiple
F19.0 Mental and behavioural disorders due to multiple drug use and use of psychoactive substances, acute intoxication	F19.0	Main analysis	Drug unspecified or multiple
F19.1 Mental and behavioural disorders due to multiple drug use and use of psychoactive substances, harmful use	F19.1	Main analysis	Drug unspecified or multiple
F19.2 Mental and behavioural disorders due to multiple drug use and use of psychoactive substances, dependence syndrome	F19.2	Main analysis	Drug unspecified or multiple
F19.3 Mental and behavioural disorders due to multiple drug use and use of psychoactive substances, withdrawal state	F19.3	Main analysis	Drug unspecified or multiple
F19.4 Mental and behavioural disorders due to multiple drug use and use of psychoactive substances, withdrawal state with delirium	F19.4	Main analysis	Drug unspecified or multiple
F19.5 Mental and behavioural disorders due to multiple drug use and use of psychoactive substances, psychotic disorder	F19.5	Main analysis	Drug unspecified or multiple
F19.6 Mental and behavioural disorders due to multiple drug use and use of psychoactive substances, amnesic syndrome	F19.6	Main analysis	Drug unspecified or multiple
F19.7 Mental and behavioural disorders due to multiple drug use and use of psychoactive substances, residual and late-onset psychotic disorder	F19.7	Main analysis	Drug unspecified or multiple
F19.8 Mental and behavioural disorders due to multiple drug use and use of psychoactive substances, other mental and behavioural disorders	F19.8	Main analysis	Drug unspecified or multiple
F19.9 Mental and behavioural disorders due to multiple drug use and use of psychoactive substances, unspecified mental and behavioural disorder	F19.9	Main analysis	Drug unspecified or multiple
**Narcotics and psychodysleptics**			
T40 Poisoning by narcotics and psychodysleptics [hallucinogens]	T40	Main analysis	Drug unspecified or multiple

Code description	Code	Analysis	Substance type
T40.4 Other synthetic narcotics	T40.4	Main analysis	Drug unspecified or multiple
T40.6 Other and unspecified narcotics	T40.6	Main analysis	Drug unspecified or multiple
**Solvents**			
F18 Mental and behavioural disorders due to use of volatile solvents	F18	Main analysis	Sedatives and others
F18.0 Mental and behavioural disorders due to use of volatile solvents, acute intoxication	F18.0	Main analysis	Sedatives and others
F18.1 Mental and behavioural disorders due to use of volatile solvents, harmful use	F18.1	Main analysis	Sedatives and others
F18.2 Mental and behavioural disorders due to use of volatile solvents, dependence syndrome	F18.2	Main analysis	Sedatives and others
F18.3 Mental and behavioural disorders due to use of volatile solvents, withdrawal state	F18.3	Main analysis	Sedatives and others
F18.4 Mental and behavioural disorders due to use of volatile solvents, withdrawal state with delirium	F18.4	Main analysis	Sedatives and others
F18.5 Mental and behavioural disorders due to use of volatile solvents, psychotic disorder	F18.5	Main analysis	Sedatives and others
F18.6 Mental and behavioural disorders due to use of volatile solvents, amnesic syndrome	F18.6	Main analysis	Sedatives and others
F18.7 Mental and behavioural disorders due to use of volatile solvents, residual and late-onset psychotic disorder	F18.7	Main analysis	Sedatives and others
F18.8 Mental and behavioural disorders due to use of volatile solvents, other mental and behavioural disorders	F18.8	Main analysis	Sedatives and others
F18.9 Mental and behavioural disorders due to use of volatile solvents, unspecified mental and behavioural disorder	F18.9	Main analysis	Sedatives and others
T52 Toxic effect of organic solvents	T52	Main analysis	Sedatives and others
T52.0 Petroleum products	T52.0	Main analysis	Sedatives and others
T52.1 Benzene	T52.1	Main analysis	Sedatives and others
T52.8 Other organic solvents	T52.8	Main analysis	Sedatives and others
T52.9 Organic solvent, unspecified	T52.9	Main analysis	Sedatives and others

SNOMED CT-Codes

Definition	SNOMED code	ICD-10 Equivalent[2]	Analysis	Substance type
	**Alcohol**			**Alcohol**
Alcohol rehabilitation and detoxification	20093000		Main analysis	Alcohol
Alcohol intoxication	25702006		Main analysis	Alcohol
Alcohol dependence	66590003		Main analysis	Alcohol
Nondependent alcohol abuse, continuous	191882002		Main analysis	Alcohol
Nondependent alcohol abuse, episodic	191883007		Main analysis	Alcohol
Nondependent cannabis abuse	191891003		Main analysis	Alcohol
Total time drunk alcohol	228330005		Main analysis	Alcohol
Absinthe addiction	231467000		Main analysis	Alcohol
Nondependent alcohol abuse	268645007		Main analysis	Alcohol
Persistent alcohol abuse	284591009		Main analysis	Alcohol
Methanol abuse	304605000		Main analysis	Alcohol
Substance use treatment: alcohol withdrawal	386449006		Main analysis	Alcohol
Severe alcohol dependence	713862009		Main analysis	Alcohol
Alcohol detoxification	827094004		Main analysis	Alcohol

SNOMED CT-Codes				
**Definition**	**SNOMED code**	**ICD-10 Equivalent[2]**	**Analysis**	**Substance type**
Acute alcohol intoxication	1149333003		Main analysis	Alcohol
Alcohol dependence in pregnancy	10741871000119100		Main analysis	Alcohol
Toxic effect of butyl alcohol	4953006	T51	Main analysis	Alcohol
Toxic effect of propyl alcohol	6749002	T51	Main analysis	Alcohol
Alcohol hallucinosis	7052005	F10	Main analysis	Alcohol
Alcoholism	7200002	F10	Main analysis	Alcohol
Alcohol withdrawal delirium	8635005	F10	Main analysis	Alcohol
Alcohol intoxication delirium	18653004	F10	Main analysis	Alcohol
Idiosyncratic intoxication	21000000	F10	Main analysis	Alcohol
Metabolic acidosis due to methanol	25966003	T51	Main analysis	Alcohol
Alcohol-induced organic mental disorder	29212009	F10	Main analysis	Alcohol
Hangover	32553006	F10	Main analysis	Alcohol
Alcohol-induced anxiety disorder	34938008	F10	Main analysis	Alcohol
Alcohol-induced sleep disorder	41083005	F10	Main analysis	Alcohol
Alcohol-induced psychosis	42344001	F10	Main analysis	Alcohol
Alcohol-induced mood disorder	53936005	F10	Main analysis	Alcohol
Toxic effect of fusel oil	57346004	T51	Main analysis	Alcohol
Alcohol-induced psychotic disorder with delusions	61144001	F10	Main analysis	Alcohol
Toxic effect of alcohol	67426006	T51	Main analysis	Alcohol
Alcohol-induced sexual dysfunction	78524005	F10	Main analysis	Alcohol
Alcohol paranoia	79578000	F10	Main analysis	Alcohol
Diarrhoea due to alcohol intake	82047000	T51	Main analysis	Alcohol
Alcohol poisoning	82782008	T51	Main analysis	Alcohol
Uncomplicated alcohol withdrawal	85561006	F10	Main analysis	Alcohol
Toxic effect of amyl alcohol	87460008	T51	Main analysis	Alcohol
Toxic effect of denatured alcohol	89507002	T51	Main analysis	Alcohol
Drug interaction with alcohol	95906008	T51	Main analysis	Alcohol
Alcohol intake above recommended sensible limits	160592001	F10	Main analysis	Alcohol
Maternal alcohol abuse	169942003	F10	Main analysis	Alcohol
Chronic alcoholic brain syndrome	191475009	F10	Main analysis	Alcohol
Alcohol withdrawal hallucinosis	191476005	F10	Main analysis	Alcohol
Pathological alcohol intoxication	191477001	F10	Main analysis	Alcohol
Alcoholic paranoia	191478006	F10	Main analysis	Alcohol
Alcohol withdrawal syndrome	191480000	F10	Main analysis	Alcohol
Acute alcoholic intoxication in alcoholism	191802004	F10	Main analysis	Alcohol

SNOMED CT-Codes				
**Definition**	**SNOMED code**	**ICD-10 Equivalent[2]**	**Analysis**	**Substance type**
Continuous acute alcoholic intoxication in alcoholism	191804003	F10	Main analysis	Alcohol
Episodic acute alcoholic intoxication in alcoholism	191805002	F10	Main analysis	Alcohol
Continuous chronic alcoholism	191811004	F10	Main analysis	Alcohol
Episodic chronic alcoholism	191812006	F10	Main analysis	Alcohol
Mental and behavioral disorders due to use of alcohol (disorder)	192206005	F10	Main analysis	Alcohol
Mental and behavioral disorders due to use of alcohol: acute intoxication (disorder)	192207001	F10	Main analysis	Alcohol
Mental and behavioral disorders due to use of alcohol: harmful use (disorder)	192208006	F10	Main analysis	Alcohol
Mental and behavioural disorders due to use of alcohol: dependence syndrome) or (chronic alcoholism [& (addiction) or (dipsomania)]) (disorder)	192209003	F10	Main analysis	Alcohol
Mental and behavioral disorders due to use of alcohol: withdrawal state (disorder)	192210008	F10	Main analysis	Alcohol
Mental and behavioral disorders due to use of alcohol: withdrawal state with delirium (disorder)	192211007	F10	Main analysis	Alcohol
Mental and behavioural disorders due to use of alcohol: psychotic disorder (& [hallucinosis] or [jealousy] or [paranoia] or [psychosis NOS]	192212000	F10	Main analysis	Alcohol
Mental and behavioral disorders due to use of alcohol: amnesic syndrome (disorder)	192213005	F10	Main analysis	Alcohol
Mental and behavioural disorders due to use of alcohol: residual and late-onset psychotic disorder) or (chronic alcoholic brain syndrome [& dementia NOS]	192214004	F10	Main analysis	Alcohol
Mental and behavioral disorders due to use of alcohol: other mental and behavioral disorders (disorder)	192215003	F10	Main analysis	Alcohol
Mental and behavioral disorders due to use of alcohol: unspecified mental and behavioral disorder (disorder)	192216002	F10	Main analysis	Alcohol
Alcohol blood level excessive (situation)	207273009	R78.0	Main analysis	Alcohol
Ethyl alcohol causing toxic effect (disorder)	212806006	T51	Main analysis	Alcohol
Grain alcohol causing toxic effect	212807002	T51	Main analysis	Alcohol
Ethyl alcohol causing toxic effect NOS (disorder)	212808007	T51	Main analysis	Alcohol
Methyl alcohol causing toxic effect	212809004	T51	Main analysis	Alcohol
Wood alcohol causing toxic effect (disorder)	212811008	T51	Main analysis	Alcohol
Toxic effect of isopropyl alcohol	212813006	T51	Main analysis	Alcohol
Dimethyl carbinol causing toxic effect (disorder)	212814000	T51	Main analysis	Alcohol
Isopropanol causing toxic effect (disorder)	212815004	T51	Main analysis	Alcohol
Rubbing alcohol causing toxic effect (disorder)	212816003	T51	Main analysis	Alcohol
Isopropyl alcohol causing toxic effect NOS (disorder)	212817007	T51	Main analysis	Alcohol
Fusel oil causing toxic effect NOS (disorder)	212818002	T51	Main analysis	Alcohol
Other alcohol causing toxic effect (disorder)	212819005	T51	Main analysis	Alcohol
Alcohol causing toxic effect NOS (disorder)	212820004	T51	Main analysis	Alcohol
Toxic effect of other alcohols (disorder)	213687005	T51	Main analysis	Alcohol
Accidental poisoning by alcoholic beverage	216633005	T51	Main analysis	Alcohol
Accidental poisoning by denatured alcohol	216635003	T51	Main analysis	Alcohol

SNOMED CT-Codes

Definition	SNOMED code	ICD-10 Equivalent[2]	Analysis	Substance type
Accidental poisoning by methylated spirit	216636002	T51	Main analysis	Alcohol
Accidental poisoning by methanol	216640006	T51	Main analysis	Alcohol
Accidental poisoning by isopropyl alcohol	216645001	T51	Main analysis	Alcohol
Accidental poisoning by rubbing alcohol substitute	216648004	T51	Main analysis	Alcohol
Accidental poisoning by fusel oil	216651006	T51	Main analysis	Alcohol
Accidental poisoning by and exposure to alcohol, occurrence at home (event)	221843007	X45	Main analysis	Alcohol
Accidental poisoning by and exposure to alcohol, occurrence in residential institution (event)	221844001	X45	Main analysis	Alcohol
Accidental poisoning by and exposure to alcohol, occurrence at school, other institution and public administrative area (event)	221845000	X45	Main analysis	Alcohol
Accidental poisoning by and exposure to alcohol, occurrence at sports and athletics area (event)	221846004	X45	Main analysis	Alcohol
Accidental poisoning by and exposure to alcohol, occurrence on street and highway (event)	221847008	X45	Main analysis	Alcohol
Accidental poisoning by and exposure to alcohol, occurrence at trade and service area (event)	221848003	X45	Main analysis	Alcohol
Accidental poisoning by and exposure to alcohol, occurrence at industrial and construction area (event)	221849006	X45	Main analysis	Alcohol
Accidental poisoning by and exposure to alcohol, occurrence on farm (event)	221850006	X45	Main analysis	Alcohol
Accidental poisoning by and exposure to alcohol, occurrence at other specified place (event)	221851005	X45	Main analysis	Alcohol
Accidental poisoning by and exposure to alcohol, occurrence at unspecified place (event)	221852003	X45	Main analysis	Alcohol
Intentional self-poisoning by and exposure to alcohol (event)	222103001	X65	Main analysis	Alcohol
Intentional self-poisoning by and exposure to alcohol, occurrence at home (event)	222104007	X65	Main analysis	Alcohol
Intentional self-poisoning by and exposure to alcohol, occurrence in residential institution (event)	222105008	X65	Main analysis	Alcohol
Intentional self-poisoning by and exposure to alcohol, occurrence at school, other institution and public administrative area (event)	222106009	X65	Main analysis	Alcohol
Intentional self-poisoning by and exposure to alcohol, occurrence at sports and athletics area (event)	222107000	X65	Main analysis	Alcohol
Intentional self-poisoning by and exposure to alcohol, occurrence on street and highway (event)	222108005	X65	Main analysis	Alcohol
Intentional self-poisoning by and exposure to alcohol, occurrence at trade and service area (event)	222110007	X65	Main analysis	Alcohol
Intentional self-poisoning by and exposure to alcohol, occurrence at industrial and construction area (event)	222111006	X65	Main analysis	Alcohol
Intentional self-poisoning by and exposure to alcohol, occurrence on farm (event)	222112004	X65	Main analysis	Alcohol
Intentional self-poisoning by and exposure to alcohol, occurrence at other specified place (event)	222113009	X65	Main analysis	Alcohol
Intentional self-poisoning by and exposure to alcohol, occurrence at unspecified place (event)	222114003	X65	Main analysis	Alcohol
Poisoning by and exposure to alcohol, undetermined intent (event)	222702003	Y15	Main analysis	Alcohol
Poisoning by and exposure to alcohol, occurrence at home, undetermined intent (event)	222703008	Y15	Main analysis	Alcohol
Poisoning by and exposure to alcohol, occurrence in residential institution, undetermined intent (event)	222704002	Y15	Main analysis	Alcohol
Poisoning by and exposure to alcohol, occurrence at school, other institution and public administrative area, undetermined intent (event)	222705001	Y15	Main analysis	Alcohol
Poisoning by and exposure to alcohol, occurrence at sports and athletics area, undetermined intent (event)	222706000	Y15	Main analysis	Alcohol
Poisoning by and exposure to alcohol, occurrence on street and highway, undetermined intent (event)	222707009	Y15	Main analysis	Alcohol

SNOMED CT-Codes				
**Definition**	**SNOMED code**	**ICD-10 Equivalent[2]**	**Analysis**	**Substance type**
Poisoning by and exposure to alcohol, occurrence at trade and service area, undetermined intent (event)	222708004	Y15	Main analysis	Alcohol
Poisoning by and exposure to alcohol, occurrence at industrial and construction area, undetermined intent (event)	222709007	Y15	Main analysis	Alcohol
Poisoning by and exposure to alcohol, occurrence on farm, undetermined intent (event)	222710002	Y15	Main analysis	Alcohol
Poisoning by and exposure to alcohol, occurrence at other specified place, undetermined intent (event)	222711003	Y15	Main analysis	Alcohol
Poisoning by and exposure to alcohol, occurrence at unspecified place, undetermined intent (event)	222713000	Y15	Main analysis	Alcohol
Evidence of alcohol involvement determined by blood alcohol level (navigational concept)	223333005	Y90	Main analysis	Alcohol
Evidence of alcohol involvement determined by blood alcohol level of less than 20 mg/100 ml (navigational concept)	223334004	Y90	Main analysis	Alcohol
Evidence of alcohol involvement determined by blood alcohol level of 20-39 mg/100 ml (navigational concept)	223335003	Y90	Main analysis	Alcohol
Evidence of alcohol involvement determined by blood alcohol level of 40-59 mg/100 ml (navigational concept)	223336002	Y90	Main analysis	Alcohol
Evidence of alcohol involvement determined by blood alcohol level of 60-79 mg/100 ml (navigational concept)	223337006	Y90	Main analysis	Alcohol
Evidence of alcohol involvement determined by blood alcohol level of 80-99 mg/100 ml (navigational concept)	223338001	Y90	Main analysis	Alcohol
Evidence of alcohol involvement determined by blood alcohol level of 100-119 mg/100 ml (navigational concept)	223339009	Y90	Main analysis	Alcohol
Evidence of alcohol involvement determined by blood alcohol level of 120-199 mg/100 ml (navigational concept)	223340006	Y90	Main analysis	Alcohol
Evidence of alcohol involvement determined by blood alcohol level of 200-239 mg/100 ml (navigational concept)	223341005	Y90	Main analysis	Alcohol
Evidence of alcohol involvement determined by blood alcohol level of 240 mg/100 ml or more (navigational concept)	223342003	Y90	Main analysis	Alcohol
Evidence of alcohol involvement determined by presence of alcohol in blood, level not specified (navigational concept)	223343008	Y90	Main analysis	Alcohol
Evidence of alcohol involvement determined by level of intoxication (navigational concept)	223344002	Y91	Main analysis	Alcohol
Evidence of alcohol involvement determined by level of intoxication, mild alcohol intoxication (navigational concept)	223345001	Y91	Main analysis	Alcohol
Evidence of alcohol involvement determined by level of intoxication, moderate alcohol intoxication (navigational concept)	223346000	Y91	Main analysis	Alcohol
Evidence of alcohol involvement determined by level of intoxication, severe alcohol intoxication (navigational concept)	223347009	Y91	Main analysis	Alcohol
Evidence of alcohol involvement determined by level of intoxication, very severe alcohol intoxication (navigational concept)	223348004	Y91	Main analysis	Alcohol
Evidence of alcohol involvement determined by level of intoxication, alcohol involvement, not otherwise specified (navigational concept)	223349007	Y91	Main analysis	Alcohol
Drinks in morning to get rid of hangover	228310006	F10	Main analysis	Alcohol
Binge drinker	228315001	F10	Main analysis	Alcohol

SNOMED CT-Codes				
**Definition**	**SNOMED code**	**ICD-10 Equivalent[2]**	**Analysis**	**Substance type**
Alcoholic binges exceeding sensible amounts	228316000	F10	Main analysis	Alcohol
Alcoholic binges exceeding safe amounts	228317009	F10	Main analysis	Alcohol
Drinking episode	228322009	F10	Main analysis	Alcohol
Drinking bout	228323004	F10	Main analysis	Alcohol
Unable to abstain from drinking	228341007	F10	Main analysis	Alcohol
Behavioural tolerance to alcohol	228350009	F10	Main analysis	Alcohol
Physical tolerance to alcohol	228351008	F10	Main analysis	Alcohol
Reverse tolerance to alcohol	228353006	F10	Main analysis	Alcohol
Drink driving	228354000	F10	Main analysis	Alcohol
Persistent effect of alcohol	228357007	F10	Main analysis	Alcohol
Alcoholic coma	230800004	Y91	Main analysis	Alcohol
Accidental exposure to alcohol	242263000	X45	Main analysis	Alcohol
Accidental exposure to ethanol	242265007	X45	Main analysis	Alcohol
Chronic alcoholism (disorder)	268639004	F10	Main analysis	Alcohol
Mental and behavioral disorders due to use of alcohol: dependence syndrome (disorder)	268683008	F10	Main analysis	Alcohol
Mental and behavioral disorders due to use of alcohol: psychotic disorder (disorder)	268684002	F10	Main analysis	Alcohol
Mental and behavioral disorders due to use of alcohol: residual and late-onset psychotic disorder (disorder)	268685001	F10	Main analysis	Alcohol
Accidental poisoning by alcohol	269765000	T51	Main analysis	Alcohol
Finding of alcohol in blood	274776000	R78.0	Main analysis	Alcohol
Alcoholic macrocytosis	278363000	X45	Main analysis	Alcohol
Accidental poisoning with ethyl alcohol	287166006	T51	Main analysis	Alcohol
Abstinent alcoholic	300939009	F10	Main analysis	Alcohol
Ethanol abuse (finding)	304606004	F10	Main analysis	Alcohol
Alcohol withdrawal-induced convulsion	308742005	F10	Main analysis	Alcohol
Pain in lymph nodes after alcohol consumption	315226008	T51	Main analysis	Alcohol
Alcohol-related fit	361267005	Y91	Main analysis	Alcohol
Ethanol in blood specimen above reference range	441685000	R78.0	Main analysis	Alcohol
Ethanol in blood specimen above legal threshold for operating vehicle	442669008	R78.0	Main analysis	Alcohol
Poisoning by benzene	442764005	T51	Main analysis	Alcohol
Alcohol in blood specimen above reference range	442766007	R78.0	Main analysis	Alcohol
Mild alcohol dependence	713583005	F10	Main analysis	Alcohol
Moderate alcohol dependence	714829008	F10	Main analysis	Alcohol
Disorder due to alcohol abuse	288021000119107	F10	Main analysis	Alcohol
Perceptual disturbance due to alcohol withdrawal	288041000119101	F10	Main analysis	Alcohol
Alcohol dependence in childbirth	10755041000119100	F10	Main analysis	Alcohol

SNOMED CT-Codes				
**Definition**	**SNOMED code**	**ICD-10 Equivalent[2]**	**Analysis**	**Substance type**
current drinking	219006		Main analysis	Alcohol
Alcoholism counselling	24165007		Main analysis	Alcohol
Smell of alcohol on breath	28045007		Main analysis	Alcohol
Alcohol rehabilitation	35637008		Main analysis	Alcohol
Referral to alcoholism rehabilitation service (procudeure)	38670004		Main analysis	Alcohol
Substance with alcohol structure	53041004		Main analysis	Alcohol
Alcoholic beverage	53527002		Main analysis	Alcohol
Heavy drinker	86933000		Main analysis	Alcohol
Alcohol intolerance	102612005		Main analysis	Alcohol
Feeling intoxicated	102897001		Main analysis	Alcohol
Under care of community alcohol team	135827004		Main analysis	Alcohol
Alcohol intake	160573003		Main analysis	Alcohol
Suspect alcohol abuse - denied	160581002		Main analysis	Alcohol
Alcohol intake within recommended sensible limits	160593006		Main analysis	Alcohol
Alcohol consumption NOS	160599005		Main analysis	Alcohol
O/E - breath - alcohol smell	163184002		Main analysis	Alcohol
Alcohol leaflet given	183098002		Main analysis	Alcohol
Admitted to alcohol detoxification centre	183486001		Main analysis	Alcohol
Other alcoholic psychosis	191479003		Main analysis	Alcohol
Other alcoholic psychosis NOS	191481001		Main analysis	Alcohol
Alcoholic psychosis NOS	191482008		Main analysis	Alcohol
Acute alcoholic intoxication, unspecified, in alcoholism	191803009		Main analysis	Alcohol
Acute alcoholic intoxication in alcoholism NOS	191807005		Main analysis	Alcohol
Unspecified chronic alcoholism	191809008		Main analysis	Alcohol
Chronic alcoholism NOS	191814007		Main analysis	Alcohol
Alcohol dependence syndrome NOS	191815008		Main analysis	Alcohol
Nondepenedent alcohol abuse, NOS	191885000		Main analysis	Alcohol
Methanol causing toxic effect	212810009		Main analysis	Alcohol
Methyl alcohol causing toxic effect NOS	212812001		Main analysis	Alcohol
Accidental poisoning by alcohol, NEC	216632000		Main analysis	Alcohol
Accidental poisoning by other ethyl alcohol and its products	216634004		Main analysis	Alcohol
Accidental poisoning by grain alcohol NOS	216637006		Main analysis	Alcohol
Accidental poisoning by ethanol, NOS	216638001		Main analysis	Alcohol
Accidental poisoning by ethyl alcohol NOS	216639009		Main analysis	Alcohol
Accidental poisoning by wood alcohol	216643008		Main analysis	Alcohol

**SNOMED CT-Codes**				
**Definition**	**SNOMED code**	**ICD-10 Equivalent[2]**	**Analysis**	**Substance type**
Accidental poisoning by methyl alcohol NOS	216644002		Main analysis	Alcohol
Accidental poisoning by dimethyl carbinol	216646000		Main analysis	Alcohol
Accidental poisoning by secondary propyl alcohol	216649007		Main analysis	Alcohol
Accidental poisoning by isopropyl alcohol NOS	216650007		Main analysis	Alcohol
Accidental poisoning by other alcohols	216652004		Main analysis	Alcohol
Accidental poisoning by alcohol NOS	216653009		Main analysis	Alcohol
[X]Accidental poisoning by and exposure to alcohol	221842002		Main analysis	Alcohol
Finding relating to alcohol drinking behaviour	228273003		Main analysis	Alcohol
Problem drinker (finding)	228281002		Main analysis	Alcohol
Drinks alcohol evenly through week	228312003		Main analysis	Alcohol
Drinks alcohol unevenly through week	228313008		Main analysis	Alcohol
binge drinking	228326007		Main analysis	Alcohol
Feels effect of alcohol at work	228358002		Main analysis	Alcohol
Feels afraid of being an alcoholic	228364009		Main analysis	Alcohol
Inebriety NOS	231464007		Main analysis	Alcohol
Drunkenness NOS	231465008		Main analysis	Alcohol
[D]Alcohol blood elevated	274257003		Main analysis	Alcohol
Alcohol products adverse reaction	292880007		Main analysis	Alcohol
Alcohol products allergy	294420000		Main analysis	Alcohol
[V] Alcohol use	302237007		Main analysis	Alcohol
[V] Alcohol use	307730003		Main analysis	Alcohol
Ingestible alcohol	311492009		Main analysis	Alcohol
[V]Alcohol rehabilitation	316322002		Main analysis	Alcohol
[V]Alcohol abuse counselling and surveillance	316494009		Main analysis	Alcohol
Alcohol intake - finding	365967005		Main analysis	Alcohol
Pattern of alcohol consumption through week - finding	365973006		Main analysis	Alcohol
Alcohol abuse prevention	408945004		Main analysis	Alcohol
Alcohol abuse prevention assessment	408946003		Main analysis	Alcohol
Alcohol abuse prevention education	408947007		Main analysis	Alcohol
Alcohol abuse prevention management	408948002		Main analysis	Alcohol
Alcohol + dextrose	412198003		Main analysis	Alcohol
Alcohol consumption counselling	413473000		Main analysis	Alcohol
Suspected alcohol abuse	415685003		Main analysis	Alcohol
Referral to community drug and alcohol team	417096006		Main analysis	Alcohol
Alcohol induced hallucinations	417633001		Main analysis	Alcohol

SNOMED CT-Codes				
**Definition**	**SNOMED code**	**ICD-10 Equivalent[2]**	**Analysis**	**Substance type**
Ethanol	419442005		Main analysis	Alcohol
Alcohol agent	419572002		Main analysis	Alcohol
Allergy to ethanol	420140004		Main analysis	Alcohol
Alcohol consumption during pregnancy	427013000		Main analysis	Alcohol
Alcohol withdrawal scale	429501006		Main analysis	Alcohol
Alcohol intake exceeds recommended daily limit	429775004		Main analysis	Alcohol
Referral to specialist alcohol treatment service	431260004		Main analysis	Alcohol
Assessment using alcohol withdrawal scale	445628007		Main analysis	Alcohol
Alcohol abuse	15167005		Main analysis	Alcohol
**Cannabis/Cannabinoids**				
Cannabis use disorder	37344009		Main analysis	Cannabis
Cannabis intoxication delirium	39807006		Main analysis	Cannabis
Cannabis dependence, episodic	191838006		Main analysis	Cannabis
Nondependent cannabis abuse, continuous	191893000		Main analysis	Cannabis
Nondependent cannabis abuse, episodic	191894006		Main analysis	Cannabis
Cannabis misuse	428823006		Main analysis	Cannabis
Synthetic cannabinoid abuse	737336003		Main analysis	Cannabis
Mental and behavioural disorders due to use of cannabinoids	26714005	F12	Main analysis	Cannabis
Mental and behavioural disorders due to use of cannabinoids	39951001	F12	Main analysis	Cannabis
Mental and behavioural disorders due to use of cannabinoids	63649001	F12	Main analysis	Cannabis
Mental and behavioural disorders due to use of cannabinoids	77355000	F12	Main analysis	Cannabis
Mental and behavioural disorders due to use of cannabinoids	85005007	F12	Main analysis	Cannabis
Mental and behavioural disorders due to use of cannabinoids	268641003	F12	Main analysis	Cannabis
Mental and behavioural disorders due to use of cannabinoids	703848005	F12	Main analysis	Cannabis
**Drug unspecified or multiple**				
Psychoactive substance dependence	2403008		Main analysis	Drug unspecified or multiple
Dependent drug abuse	6525002		Main analysis	Drug unspecified or multiple
Drug habituation	9769006		Main analysis	Drug unspecified or multiple
Combined alcohol and drug rehabilitation and detoxification	23915005		Main analysis	Drug unspecified or multiple
Drug abuse	26416006		Main analysis	Drug unspecified or multiple
Non dependent drug abuse	49540005		Main analysis	Drug unspecified or multiple
Drug rehabilitation and detoxification	56876005		Main analysis	Drug unspecified or multiple
Drug detoxification	61480009		Main analysis	Drug unspecified or multiple
Detoxification psychiatric therapy for alcoholism	64297001		Main analysis	Drug unspecified or multiple
Substance use disorder	66214007		Main analysis	Drug unspecified or multiple

SNOMED CT-Codes				
**Definition**	**SNOMED code**	**ICD-10 Equivalent[2]**	**Analysis**	**Substance type**
Narcotic drug user	70545002		Main analysis	Drug unspecified or multiple
Combined alcohol and drug detoxification	87106005		Main analysis	Drug unspecified or multiple
Psychoactive substance abuse	91388009		Main analysis	Drug unspecified or multiple
Maternal drug use	95607001		Main analysis	Drug unspecified or multiple
Occasional drug abuser	105546006		Main analysis	Drug unspecified or multiple
Chronic drug abuse	110281001		Main analysis	Drug unspecified or multiple
Dependent drug detoxification	182969009		Main analysis	Drug unspecified or multiple
Nondependent mixed drug abuse	191934007		Main analysis	Drug unspecified or multiple
Nondependent mixed drug abuse, continuous	191936009		Main analysis	Drug unspecified or multiple
Nondependent mixed drug abuse, episodic	191937000		Main analysis	Drug unspecified or multiple
Injecting drug user	226034001		Main analysis	Drug unspecified or multiple
Long-term drug misuser	228371004		Main analysis	Drug unspecified or multiple
Poly-drug misuser	228372006		Main analysis	Drug unspecified or multiple
Drug addict	228373001		Main analysis	Drug unspecified or multiple
Notified addict	228374007		Main analysis	Drug unspecified or multiple
Misuses drugs orally	228375008		Main analysis	Drug unspecified or multiple
Inhales drugs	228376009		Main analysis	Drug unspecified or multiple
Smokes drugs	228377000		Main analysis	Drug unspecified or multiple
Smokes drugs in cigarette form	228378005		Main analysis	Drug unspecified or multiple
Smokes drugs through a pipe	228379002		Main analysis	Drug unspecified or multiple
Sniffs drugs	228381000		Main analysis	Drug unspecified or multiple
Misuses drugs vaginally	228382007		Main analysis	Drug unspecified or multiple
Misuses drugs rectally	228383002		Main analysis	Drug unspecified or multiple
Misuses drugs sublingually	228384008		Main analysis	Drug unspecified or multiple
Injects drugs subcutaneously	228386005		Main analysis	Drug unspecified or multiple
Injects drugs intramuscularly	228387001		Main analysis	Drug unspecified or multiple
Intravenous drug user	228388006		Main analysis	Drug unspecified or multiple
Groin injector	228389003		Main analysis	Drug unspecified or multiple
Frequency of drug misuse	228390007		Main analysis	Drug unspecified or multiple
Drug injection behaviour	228391006		Main analysis	Drug unspecified or multiple
Drug injecting equipment hygiene	228394003		Main analysis	Drug unspecified or multiple
Cleaning of drug injection equipment	228401002		Main analysis	Drug unspecified or multiple
Priority of drug-related activities	228415000		Main analysis	Drug unspecified or multiple
Time devoted to drug-related activities	228421001		Main analysis	Drug unspecified or multiple
Time spent obtaining drugs	228422008		Main analysis	Drug unspecified or multiple

**SNOMED CT-Codes**				
**Definition**	**SNOMED code**	**ICD-10 Equivalent[2]**	**Analysis**	**Substance type**
Time spent taking drugs	228423003		Main analysis	Drug unspecified or multiple
Routine of drug-related activities	228425005		Main analysis	Drug unspecified or multiple
Drug-related rituals	228429004		Main analysis	Drug unspecified or multiple
Drug addiction therapy	266707007		Main analysis	Drug unspecified or multiple
Illicit drug use	307052004		Main analysis	Drug unspecified or multiple
Habitual drug user	361049005		Main analysis	Drug unspecified or multiple
Substance abuser	361055000		Main analysis	Drug unspecified or multiple
Substance use treatment: drug withdrawal	386450006		Main analysis	Drug unspecified or multiple
Substance use treatment: overdose	386451005		Main analysis	Drug unspecified or multiple
Drug dependence home detoxification	414054004		Main analysis	Drug unspecified or multiple
Drug dependence self detoxification	414056002		Main analysis	Drug unspecified or multiple
Continuous use of drugs	416262000		Main analysis	Drug unspecified or multiple
Preoccupied with substance misuse	416437003		Main analysis	Drug unspecified or multiple
Episodic use of drugs	417252002		Main analysis	Drug unspecified or multiple
Current drug user	417284009		Main analysis	Drug unspecified or multiple
Recreational drug user	424848002		Main analysis	Drug unspecified or multiple
Episodic drug abuse	425533007		Main analysis	Drug unspecified or multiple
Drug use behaviour during pregnancy	440664005		Main analysis	Drug unspecified or multiple
Amount of money spent per day on drug habit	442229000		Main analysis	Drug unspecified or multiple
Polysubstance abuse	445273005		Main analysis	Drug unspecified or multiple
Illicit drug overdose	708079007		Main analysis	Drug unspecified or multiple
Novel psychoactive substance misuse	713775002		Main analysis	Drug unspecified or multiple
Illicit drug injection in last 12 months	741063003		Main analysis	Drug unspecified or multiple
Intoxication caused by recreational drug misuse	772808000		Main analysis	Drug unspecified or multiple
Intoxication	1149322001		Main analysis	Drug unspecified or multiple
Psychostimulant dependence episodic	1231333004		Main analysis	Drug unspecified or multiple
Substance dependence	1254812006		Main analysis	Drug unspecified or multiple
Substance dependence, episodic	1254974001		Main analysis	Drug unspecified or multiple
Substance dependence in childbirth	1254987007		Main analysis	Drug unspecified or multiple
Combined substance dependence, excluding opioid, continuous	1255017007		Main analysis	Drug unspecified or multiple
Combined substance dependence, excluding opioid, in remission	1255020004		Main analysis	Drug unspecified or multiple
Postpartum substance dependence	1259017004		Main analysis	Drug unspecified or multiple
Patient self-medicating with illicit substance	1334001000168100		Main analysis	Drug unspecified or multiple
Drug addiction therapy using non-opioid medicine	1545151000168100		Main analysis	Drug unspecified or multiple
Management of simple withdrawal from substance use	1648321000168100		Main analysis	Drug unspecified or multiple

SNOMED CT-Codes

Definition	SNOMED code	ICD-10 Equivalent[2]	Analysis	Substance type
Management of complex withdrawal from substance use	1648331000168100		Main analysis	Drug unspecified or multiple
Episodic polysubstance dependence	16076691000119100		Main analysis	Drug unspecified or multiple
Mental and behavioural disorders due to multiple drug use and use of other psychoactive substances	11061003	F22	Main analysis	Drug unspecified or multiple
Poisoning by other and unspecified narcotics	11196001	T40.6	Main analysis	Drug unspecified or multiple
Mental and behavioural disorders due to multiple drug use and use of other psychoactive substances	11387009	F23	Main analysis	Drug unspecified or multiple
Mental and behavioural disorders due to multiple drug use and use of other psychoactive substances	28368009	F25	Main analysis	Drug unspecified or multiple
Mental and behavioural disorders due to multiple drug use and use of other psychoactive substances	32709003	F26	Main analysis	Drug unspecified or multiple
Mental and behavioural disorders due to multiple drug use and use of other psychoactive substances	43242008	F27	Main analysis	Drug unspecified or multiple
Mental and behavioural disorders due to multiple drug use and use of other psychoactive substances	50026000	F28	Main analysis	Drug unspecified or multiple
Mental and behavioural disorders due to multiple drug use and use of other psychoactive substances	51339003	F29	Main analysis	Drug unspecified or multiple
Mental and behavioural disorders due to multiple drug use and use of other psychoactive substances	74934004	F32	Main analysis	Drug unspecified or multiple
Mental and behavioural disorders due to multiple drug use and use of other psychoactive substances	84584008	F33	Main analysis	Drug unspecified or multiple
Mental and behavioural disorders due to multiple drug use and use of other psychoactive substances	191483003	F19	Main analysis	Drug unspecified or multiple
Mental and behavioural disorders due to multiple drug use and use of other psychoactive substances	191484009	F19	Main analysis	Drug unspecified or multiple
Mental and behavioural disorders due to multiple drug use and use of other psychoactive substances	191485005	F19	Main analysis	Drug unspecified or multiple
Mental and behavioural disorders due to multiple drug use and use of other psychoactive substances	191486006	F19	Main analysis	Drug unspecified or multiple
Mental and behavioural disorders due to multiple drug use and use of other psychoactive substances	191492000	F19	Main analysis	Drug unspecified or multiple
Mental and behavioural disorders due to multiple drug use and use of other psychoactive substances	191494004	F19	Main analysis	Drug unspecified or multiple
Mental and behavioural disorders due to multiple drug use and use of other psychoactive substances	191495003	F19	Main analysis	Drug unspecified or multiple
Mental and behavioural disorders due to multiple drug use and use of other psychoactive substances	191496002	F19	Main analysis	Drug unspecified or multiple
Mental and behavioural disorders due to multiple drug use and use of other psychoactive substances	191816009	F19	Main analysis	Drug unspecified or multiple
Mental and behavioural disorders due to multiple drug use and use of other psychoactive substances	191865004	F19	Main analysis	Drug unspecified or multiple
Mental and behavioural disorders due to multiple drug use and use of other psychoactive substances	191873008	F19	Main analysis	Drug unspecified or multiple
Mental and behavioural disorders due to multiple drug use and use of other psychoactive substances	191939002	F19	Main analysis	Drug unspecified or multiple
Mental and behavioural disorders due to multiple drug use and use of other psychoactive substances	228438002	F19	Main analysis	Drug unspecified or multiple
Mental and behavioural disorders due to multiple drug use and use of other psychoactive substances	231451006	F19	Main analysis	Drug unspecified or multiple
Mental and behavioural disorders due to multiple drug use and use of other psychoactive substances	231466009	F19	Main analysis	Drug unspecified or multiple
Mental and behavioural disorders due to multiple drug use and use of other psychoactive substances	231481003	F19	Main analysis	Drug unspecified or multiple
Mental and behavioural disorders due to multiple drug use and use of other psychoactive substances	231482005	F19	Main analysis	Drug unspecified or multiple
Mental and behavioural disorders due to multiple drug use and use of other psychoactive substances	247702001	F19	Main analysis	Drug unspecified or multiple
Poisoning by other and unspecified narcotics	290220008	T40.6	Main analysis	Drug unspecified or multiple
Poisoning by other and unspecified narcotics	290221007	T40.6	Main analysis	Drug unspecified or multiple
Poisoning by other and unspecified narcotics	290222000	T40.6	Main analysis	Drug unspecified or multiple
Poisoning by other synthetic narcotics	295154004	T40.4	Main analysis	Drug unspecified or multiple
Poisoning by other synthetic narcotics	295167001	T40.4	Main analysis	Drug unspecified or multiple

SNOMED CT-Codes				
**Definition**	**SNOMED code**	**ICD-10 Equivalent[2]**	**Analysis**	**Substance type**
Poisoning by other synthetic narcotics	295169003	T40.4	Main analysis	Drug unspecified or multiple
Poisoning by other synthetic narcotics	295190006	T40.4	Main analysis	Drug unspecified or multiple
Poisoning by other synthetic narcotics	295193008	T40.4	Main analysis	Drug unspecified or multiple
Poisoning by other synthetic narcotics	295194002	T40.4	Main analysis	Drug unspecified or multiple
Poisoning by other and unspecified narcotics	295213004	T40.6	Main analysis	Drug unspecified or multiple
Poisoning by other and unspecified narcotics	297199006	T40.6	Main analysis	Drug unspecified or multiple
Mental and behavioural disorders due to multiple drug use and use of other psychoactive substances	363101005	F19	Main analysis	Drug unspecified or multiple
Mental and behavioural disorders due to multiple drug use and use of other psychoactive substances	363314000	F19	Main analysis	Drug unspecified or multiple
Mental and behavioural disorders due to multiple drug use and use of other psychoactive substances	365984004	F19	Main analysis	Drug unspecified or multiple
Mental and behavioural disorders due to multiple drug use and use of other psychoactive substances	396344000	F19	Main analysis	Drug unspecified or multiple
Mental and behavioural disorders due to multiple drug use and use of other psychoactive substances	416119007	F19	Main analysis	Drug unspecified or multiple
Mental and behavioural disorders due to multiple drug use and use of other psychoactive substances	429299000	F19	Main analysis	Drug unspecified or multiple
Mental and behavioural disorders due to multiple drug use and use of other psychoactive substances	429672007	F19	Main analysis	Drug unspecified or multiple
Mental and behavioural disorders due to multiple drug use and use of other psychoactive substances	442351006	F19	Main analysis	Drug unspecified or multiple
Combined alcohol and drug rehabilitation	62213004		Main analysis	Drug unspecified or multiple
**Opioids**				
Opioid use disorder	5602001	F11	Main analysis	Opioids
Opioid intoxication delirium	52866005		Main analysis	Opioids
Opioid dependence	75544000		Main analysis	Opioids
Episodic opioid dependence	191820008		Main analysis	Opioids
Nondependent opioid abuse	191909007		Main analysis	Opioids
Nondependent opioid abuse, continuous	191912005		Main analysis	Opioids
Nondependent opioid abuse, episodic	191913000		Main analysis	Opioids
Heroin dependence	231477003		Main analysis	Opioids
Methadone dependence	231478008		Main analysis	Opioids
Morphine dependence	231479000		Main analysis	Opioids
Opium dependence	231480002		Main analysis	Opioids
Drug addiction therapy using methadone	310653000		Main analysis	Opioids
Opiate misuse	428819003		Main analysis	Opioids
Methadone misuse	429512006		Main analysis	Opioids
Drug addiction therapy using buprenorphine	792901003		Main analysis	Opioids
Drug addiction therapy using buprenorphine and naloxone	792902005		Main analysis	Opioids
Combined opioid with non-opioid substance dependence, episodic	1255015004		Main analysis	Opioids
Opioid dependence, on agonist therapy	1081000119105		Main analysis	Opioids
Intravenous nondependent opioid abuse	145121000119106		Main analysis	Opioids

SNOMED CT-Codes				
**Definition**	**SNOMED code**	**ICD-10 Equivalent[2]**	**Analysis**	**Substance type**
Opioid agonist treatment	1515351000168100		Main analysis	Opioids
Maintenance of opioid agonist treatment	1640611000168100		Main analysis	Opioids
Initiation of opioid agonist treatment	1640661000168100		Main analysis	Opioids
Poisoning by heroin	13187008	T40.1	Main analysis	Opioids
Mental and behavioural disorders due to use of opioids	14784000	F11	Main analysis	Opioids
Poisoning by methadone	60199004	T40.3	Main analysis	Opioids
Mental and behavioural disorders due to use of opioids	77721001	F11	Main analysis	Opioids
Mental and behavioural disorders due to use of opioids	87132004	F11	Main analysis	Opioids
Mental and behavioural disorders due to use of opioids	191819002	F11	Main analysis	Opioids
Poisoning by heroin	216463005	T40.1	Main analysis	Opioids
Poisoning by methadone	216464004	T40.4	Main analysis	Opioids
Poisoning by other opioids	242828004	T40.2	Main analysis	Opioids
Poisoning by heroin	242829007	T40.1	Main analysis	Opioids
Poisoning by methadone	242831003	T40.5	Main analysis	Opioids
Poisoning by heroin	290182008	T40.1	Main analysis	Opioids
Poisoning by heroin	290183003	T40.1	Main analysis	Opioids
Poisoning by methadone	295161000	T40.6	Main analysis	Opioids
Poisoning by methadone	295163002	T40.6	Main analysis	Opioids
Poisoning by methadone	295164008	T40.6	Main analysis	Opioids
Poisoning by other opioids	295165009	T40.3	Main analysis	Opioids
Poisoning by other opioids	295170002	T40.2	Main analysis	Opioids
Poisoning by other opioids	295171003	T40.2	Main analysis	Opioids
Poisoning by other opioids	295172005	T40.2	Main analysis	Opioids
Poisoning by other opioids	295173000	T40.2	Main analysis	Opioids
Poisoning by heroin	295174006	T40.1	Main analysis	Opioids
Poisoning by heroin	295175007	T40.1	Main analysis	Opioids
Poisoning by heroin	295176008	T40.1	Main analysis	Opioids
Poisoning by other opioids	295184007	T40.2	Main analysis	Opioids
Poisoning by other opioids	295186009	T40.2	Main analysis	Opioids
Mental and behavioural disorders due to use of opioids	426001001	F11	Main analysis	Opioids
Overdose of opiate	242253008		Main analysis	Opioids
**Sedatives and others**				
Sedative, hypnotic AND/OR anxiolytic intoxication delirium	5444000		Sensitivity analysis	Sedatives and others
PCP abuse	7071007		Main analysis	Sedatives and others
Hallucinogen dependence	38247002		Main analysis	Sedatives and others

SNOMED CT-Codes				
**Definition**	**SNOMED code**	**ICD-10 Equivalent[2]**	**Analysis**	**Substance type**
Hallucinogen intoxication	50320000		Main analysis	Sedatives and others
Inhalant intoxication	60901005		Main analysis	Sedatives and others
Sedative abuse	64386003		Main analysis	Sedatives and others
Inhalant abuse	70340006		Main analysis	Sedatives and others
Hallucinogen abuse	74851005		Main analysis	Sedatives and others
Abuses volatile solvents	105549004		Main analysis	Sedatives and others
Hallucinogen dependence, continuous	191849000		Main analysis	Sedatives and others
Glue sniffing dependence, episodic	191856006		Main analysis	Sedatives and others
Nondependent hallucinogen abuse, continuous	191899001		Main analysis	Sedatives and others
Nondependent hallucinogen abuse, episodic	191900006		Main analysis	Sedatives and others
Barbiturate abuse	231462006		Main analysis	Sedatives and others
Lysergic acid diethylamide dependence	231468005		Main analysis	Sedatives and others
Nondependent hallucinogen abuse	268646008		Main analysis	Sedatives and others
Psychostimulant dependence	275471001		Main analysis	Sedatives and others
Continuous inhalant abuse	426095000		Main analysis	Sedatives and others
Drug abuse, continuous	426590003		Main analysis	Sedatives and others
Episodic inhalant abuse	427229002		Main analysis	Sedatives and others
Solvent misuse	428495004		Main analysis	Sedatives and others
Barbiturate misuse	428623008		Main analysis	Sedatives and others
Ketamine abuse	724713006		Main analysis	Sedatives and others
Dependence due to ketamine	724715004		Main analysis	Sedatives and others
Harmful use of hypnotic	772999000		Main analysis	Sedatives and others
Nondependent hypnotic abuse, episodic	1230074002		Main analysis	Sedatives and others
Nondependent hypnotic abuse, continuous	1230077009		Main analysis	Sedatives and others
Nondependent hypnotic abuse	1230081009		Main analysis	Sedatives and others
Hypnotic dependence, episodic	1231160001		Main analysis	Sedatives and others
Inhalant dependence, continuous	1382581000168100		Main analysis	Sedatives and others
Continuous sedative abuse	145841000119107		Main analysis	Sedatives and others
Episodic phencyclidine abuse	1382351000168100		Main analysis	Sedatives and others
Aerosol inhalation dependence	1382391000168100		Main analysis	Sedatives and others
Petrol sniffing dependence	1382421000168100		Main analysis	Sedatives and others
Paint sniffing dependence	1382521000168100		Main analysis	Sedatives and others
Aerosol inhalation abuse	1382531000168100		Main analysis	Sedatives and others
Petrol sniffing abuse	1382541000168100		Main analysis	Sedatives and others
Paint sniffing abuse	1382551000168100		Main analysis	Sedatives and others

SNOMED CT-Codes				
**Definition**	**SNOMED code**	**ICD-10 Equivalent[2]**	**Analysis**	**Substance type**
Butane inhalation abuse	1382561000168100		Main analysis	Sedatives and others
Nitrite inhalation abuse	1382571000168100		Main analysis	Sedatives and others
Nitrous oxide inhalation abuse	1382581000168100		Main analysis	Sedatives and others
Glue sniffing abuse	145841000119107		Main analysis	Sedatives and others
Mental and behavioural disorders due to multiple drug use and use of other psychoactive substances	88320008	F18	Main analysis	Sedatives and others
Mental and behavioural disorders due to use of volatile solvents	191853003	F18	Main analysis	Sedatives and others
Mental and behavioural disorders due to use of sedatives	231473004	F13	Main analysis	Sedatives and others
Continuous phencyclidine abuse	425885002		Main analysis	Sedatives and others
**Stimulants**				
Amphetamine dependence	21647008		Main analysis	Stimulants
Cocaine intoxication	27956007		Main analysis	Stimulants
Cocaine dependence	31956009		Main analysis	Stimulants
Cocaine use disorder	78267003		Main analysis	Stimulants
Cocaine dependence, continuous	191831000		Main analysis	Stimulants
Nondependent cocaine abuse	191916008		Main analysis	Stimulants
Nondependent cocaine abuse, continuous	191918009		Main analysis	Stimulants
Nondependent cocaine abuse, episodic	191919001		Main analysis	Stimulants
Overdose of cocaine	296321004		Main analysis	Stimulants
Crack cocaine misuse	428493006		Main analysis	Stimulants
Amphetamine misuse	428659002		Main analysis	Stimulants
Cocaine misuse	429782000		Main analysis	Stimulants
Stimulant abuse	441527004		Main analysis	Stimulants
Stimulant dependence	442406005		Main analysis	Stimulants
Methamphetamine abuse	699449003		Main analysis	Stimulants
Abuse of synthetic cathinone	762504005		Main analysis	Stimulants
Synthetic cathinone dependence	762505006		Main analysis	Stimulants
Intoxication due to synthetic cathinone	762671008		Main analysis	Stimulants
Amphetamine and/or amphetamine derivative use disorder	785277001		Main analysis	Stimulants
Intoxication caused by central stimulant	1155772001		Main analysis	Stimulants
Amfetamine and/or amfetamine derivative abuse, episodic	1230071005		Main analysis	Stimulants
Amfetamine and/or amfetamine derivative abuse, continuous	1230072003		Main analysis	Stimulants
Nondependent psychostimulant abuse, continuous	1230084001		Main analysis	Stimulants
Nondependent amfetamine and/or amfetamine derivative abuse, continuous	1230085000		Main analysis	Stimulants
Nondependent psychostimulant abuse, episodic	1230086004		Main analysis	Stimulants
Nondependent amfetamine and/or amfetamine derivative abuse, episodic	1230087008		Main analysis	Stimulants

SNOMED CT-Codes				
**Definition**	**SNOMED code**	**ICD-10 Equivalent[2]**	**Analysis**	**Substance type**
Nondependent amfetamine and/or amfetamine derivative abuse	1230088003		Main analysis	Stimulants
Nondependent psychostimulant abuse	1230089006		Main analysis	Stimulants
Methylenedioxymethamphetamine dependence	1231319004		Main analysis	Stimulants
Intravenous cocaine abuse	145101000119102		Main analysis	Stimulants
Catha edulis abuse	429001000124103		Main analysis	Stimulants
Methamphetamine intoxication	12398571000119100		Main analysis	Stimulants
Mental and behavioural disorders due to use of other stimulants, including caffeine	8837000	F15	Main analysis	Stimulants
Mental and behavioural disorders due to use of other stimulants, including caffeine	45421006	F15	Main analysis	Stimulants
Mental and behavioural disorders due to use of cocaine	46975003	F14	Main analysis	Stimulants
Mental and behavioural disorders due to use of cocaine	51493001	F14	Main analysis	Stimulants
Mental and behavioural disorders due to use of cocaine	80868005	F14	Main analysis	Stimulants
Mental and behavioural disorders due to use of other stimulants, including caffeine	84758004	F15	Main analysis	Stimulants
Poisoning by cocaine	290544006	T40.5	Main analysis	Stimulants
Poisoning by cocaine	290545007	T40.5	Main analysis	Stimulants
Poisoning by cocaine	296322006	T40.5	Main analysis	Stimulants
Poisoning by cocaine	296323001	T40.5	Main analysis	Stimulants
Mental and behavioural disorders due to use of other stimulants, including caffeine	428370001	F15	Main analysis	Stimulants
Amfetamine and/or amfetamine derivative dependence continuous	1231328003		Main analysis	Stimulants

Footnotes: 1. SNOMED Clinical Terms® (SNOMED CT®) which is used by permission of the SNOMED International. All rights reserved. SNOMED CT® was originally created by the College of American Pathologists. “SNOMED”, “SNOMED CT” and “SNOMED Clinical Terms” are registered trademarks of the SNOMED International (www.snomed.org); 2. ICD-10-AM equivalent code based on mapping by Yuen 2022
